# Co-Exposure of Cardiomyocytes to IFN-γ and TNF-α Induces Mitochondrial Dysfunction and Nitro-Oxidative Stress: Implications for the Pathogenesis of Chronic Chagas Disease Cardiomyopathy

**DOI:** 10.3389/fimmu.2021.755862

**Published:** 2021-11-11

**Authors:** João Paulo Silva Nunes, Pauline Andrieux, Pauline Brochet, Rafael Ribeiro Almeida, Eduardo Kitano, André Kenji Honda, Leo Kei Iwai, Débora Andrade-Silva, David Goudenège, Karla Deysiree Alcântara Silva, Raquel de Souza Vieira, Débora Levy, Sergio Paulo Bydlowski, Frédéric Gallardo, Magali Torres, Edimar Alcides Bocchi, Miguel Mano, Ronaldo Honorato Barros Santos, Fernando Bacal, Pablo Pomerantzeff, Francisco Rafael Martins Laurindo, Priscila Camillo Teixeira, Helder I. Nakaya, Jorge Kalil, Vincent Procaccio, Christophe Chevillard, Edecio Cunha-Neto

**Affiliations:** ^1^ Laboratory of Immunology, Heart Institute (Incor), Hospital das Clínicas da Faculdade de Medicina da Universidade de São Paulo, São Paulo, Brazil; ^2^ Division of Clinical Immunology and Allergy, Faculdade de Medicina da Universidade de São Paulo, São Paulo, Brazil; ^3^ iii-Institute for Investigation in Immunology, Instituto Nacional de Ciência e Tecnologia (INCT), São Paulo, Brazil; ^4^ INSERM, UMR_1090, Aix Marseille Université, TAGC Theories and Approaches of Genomic Complexity, Institut MarMaRa, Marseille, France; ^5^ Laboratório Especial de Toxinologia Aplicada, Center of Toxins, Immune-Response and Cell Signaling (CeTICS), Instituto Butantan, São Paulo, Brazil; ^6^ Department of Biochemistry and Genetics, University Hospital of Angers, Angers, France; ^7^ Heart Failure Team, Heart Institute (Incor) Hospital das Clínicas da Faculdade de Medicina da Universidade de São Paulo, São Paulo, Brazil; ^8^ Functional Genomics and RNA-based Therapeutics Laboratory, Center for Neuroscience and Cell Biology (CNC), University of Coimbra, Coimbra, Portugal; ^9^ Division of Surgery, Heart Institute, School of Medicine, University of São Paulo, São Paulo, Brazil; ^10^ Laboratory of Vascular Biology, Heart Institute of the School of Medicine, University of São Paulo, São Paulo, Brazil; ^11^ Translational Research Sciences, Pharma Research and Early Development F. Hoffmann-La Roche, Basel, Switzerland; ^12^ Hospital Israelita Albert Einstein, São Paulo, Brazil; ^13^ MitoLab, UMR CNRS 6015-INSERM U1083, Université d’Angers, Angers, France

**Keywords:** mitochondrial dysfunction, chronic Chagas disease cardiomyopathy, interferon gamma, energy metabolism, mitochondria

## Abstract

Infection by the protozoan *Trypanosoma cruzi* causes Chagas disease cardiomyopathy (CCC) and can lead to arrhythmia, heart failure and death. Chagas disease affects 8 million people worldwide, and chronic production of the cytokines IFN-γ and TNF-α by T cells together with mitochondrial dysfunction are important players for the poor prognosis of the disease. Mitochondria occupy 40% of the cardiomyocytes volume and produce 95% of cellular ATP that sustain the life-long cycles of heart contraction. As IFN-γ and TNF-α have been described to affect mitochondrial function, we hypothesized that IFN-γ and TNF-α are involved in the myocardial mitochondrial dysfunction observed in CCC patients. In this study, we quantified markers of mitochondrial dysfunction and nitro-oxidative stress in CCC heart tissue and in IFN-γ/TNF-α-stimulated AC-16 human cardiomyocytes. We found that CCC myocardium displayed increased levels of nitro-oxidative stress and reduced mitochondrial DNA as compared with myocardial tissue from patients with dilated cardiomyopathy (DCM). IFN-γ/TNF-α treatment of AC-16 cardiomyocytes induced increased nitro-oxidative stress and decreased the mitochondrial membrane potential (ΔΨm). We found that the STAT1/NF-κB/NOS2 axis is involved in the IFN-γ/TNF-α-induced decrease of ΔΨm in AC-16 cardiomyocytes. Furthermore, treatment with mitochondria-sparing agonists of AMPK, NRF2 and SIRT1 rescues ΔΨm in IFN-γ/TNF-α-stimulated cells. Proteomic and gene expression analyses revealed that IFN-γ/TNF-α-treated cells corroborate mitochondrial dysfunction, transmembrane potential of mitochondria, altered fatty acid metabolism and cardiac necrosis/cell death. Functional assays conducted on Seahorse respirometer showed that cytokine-stimulated cells display decreased glycolytic and mitochondrial ATP production, dependency of fatty acid oxidation as well as increased proton leak and non-mitochondrial oxygen consumption. Together, our results suggest that IFN-γ and TNF-α cause direct damage to cardiomyocytes’ mitochondria by promoting oxidative and nitrosative stress and impairing energy production pathways. We hypothesize that treatment with agonists of AMPK, NRF2 and SIRT1 might be an approach to ameliorate the progression of Chagas disease cardiomyopathy.

## Introduction

Heart failure (HF) is an important worldwide public health problem. Available therapies are insufficient and do not fully address molecular abnormalities that occur in cardiomyocytes (1). Chagas disease cardiomyopathy (CCC) accounts for 25% of HF cases and is a major cause of death in Latin America (2, 3). CCC is a severe inflammatory dilated cardiomyopathy caused by persistent infection by the protozoan *Trypanosoma cruzi.* While 60% of Chagas Disease (CD) patients are mostly asymptomatic in the so-called “indeterminate” form (IF) and do not develop heart disease, CCC patients (30%, roughly 8 million people) display HF, arrhythmia, and disability (4, 5). CCC patients have 50% shorter survival rate and worse prognosis compared to patients with cardiomyopathies of non-inflammatory etiologies, such as ischemic, idiopathic and hypertensive cardiomyopathies (2).

The pathogenesis of CCC is still to be completely understood (6). Low-grade chronic *T. cruzi* persistence, which drives continued production of IFN-γ and TNF-α by Th1 T cells immune cells, plays a central pathogenic role in CCC (2, 4, 5). Indeed, omics and immunohistochemistry studies revealed higher levels of IFN-γ and TNF-α in heart tissue from CCC patients compared to other cardiomyopathies (7–11). Activation of the NF-κB/NOS2 axis by the continued production of IFN-γ/TNF-α can promote increased levels of intracellular reactive nitrogen species (RNS) which are important for parasite control (12, 13). However, long-term sustained activation of this axis may promote damaging accumulation of reactive oxygen species (ROS) through induction of NADP oxidases and overproduction of mitochondrial reactive oxygen species (ROS), such as hydrogen peroxide (H_2_O_2_) and superoxide anion ( ${\text{O}}_{2}^{-}$ ) and RNS, molecules widely known to induce mitochondrial dysfunction and disturbances of heart function (14, 15).

The heart is the most metabolically active organ in the body and has the highest content of mitochondria of any tissue (16). This is needed to meet the ever-demanding energy requirement for contraction and relaxation and about 95% of cellular ATP is utilized to support the contraction–relaxation cycle within the myocardium (16). Indeed, mitochondria contribute to the proper function of cardiomyocytes by multiple mechanisms (17). Beyond contraction, mitochondria also fine-tune cellular calcium homeostasis, apoptosis and oxidative stress and it has become increasingly clear that mitochondrial dysfunction is the source of heart energy deprivation in cardiomyopathies and heart failure of diverse etiologies (16).

Several myocardial mitochondrial enzymes were found to be selectively depressed in CCC as compared to other cardio-myopathies, including ATP synthase α, multiple fatty acid β-oxidation enzymes and creatine kinase activity [(18–20) and our unpublished observations] additionally, *in vivo* myocardial ATP production was shown to be reduced in CCC as determined by ^31^P-NMR spectroscopy (21). Taken together, results suggest that mitochondrial dysfunction may contribute to the worse prognosis of CCC. Since inflammatory cytokines IFN-γ and TNF-α are abundantly produced and are top upstream regulators of gene expression changes in in CCC myocardium (9), and the sustained ROS and RNS production are known inducers of cardiomyocyte damage and mitochondrial dysfunction (22, 23), we hypothesized that such cytokines contribute to mitochondrial dysfunction observed in CCC (5).

Thus, this study aimed to investigate 1) whether CCC myocardial tissue display increased levels of mitochondrial dysfunction and oxidative/nitrosative stress markers compared to non-chagasic dilated cardiomyopathy biopsies (DCM); 2) whether IFN-γ and TNF-α promote mitochondrial dysfunction and nitro-oxidative stress in the human cardiomyocyte cell line AC-16; 3) whether the mitochondrial dysfunction in cardiomyocytes is triggered by the cytokines through the STAT1/NF-κB/NOS2 signaling pathway; 4) whether agonists of mitochondrial protection pathways, such as AMPK, NRF2 and SIRT1 ameliorate mitochondrial dysfunction driven by the IFN-γ and TNF-α.

## Methods

### Ethics Statement

Tissue collection was approved by the Institutional Review Board of the University of São Paulo, School of Medicine (CAPPesq approval number 852/05) and written informed consent was obtained from the patients. All experimental methods comply with the Helsinki declaration.

### Patients and Sample Collection

Human left ventricular free wall heart tissue was collected from end-stage heart failure CCC patients (n=40) and non-chagasic dilated cardiomyopathy (DCM, n=31) patients (NYHA class 3 and 4) at the moment of heart transplantation. CCC patients presented positive *T. cruzi* serology and typical heart conduction abnormalities (right bundle branch block and/or left anterior division hemiblock) and had a histopathological diagnosis of myocarditis, fibrosis and hypertrophy. All heart tissue samples were cleared from pericardium and fat and quickly frozen in liquid nitrogen and stored at -80°C. The list of patients is described in **Table 1**.

**Table 1:** List of the patients included in this study.

Code	Etiology	Sex	Age	EF (%)
CCC-S01	Severe CCC	F	61	27
CCC-S02	Severe CCC	M	60	35
CCC-S03	Severe CCC	F	64	30
CCC-S04	Severe CCC	M	61	21
CCC-S05	Severe CCC	F	60	20
CCC-S06	Severe CCC	M	41	19
CCC-S07	Severe CCC	M	15	17
CCC-S08	Severe CCC	M	28	29
CCC-S09	Severe CCC	M	32	12
CCC-S10	Severe CCC	F	32	19
CCC-S11	Severe CCC	M	41	23
CCC-S12	Severe CCC	M	58	20
CCC-S13	Severe CCC	M	36	25
CCC-S14	Severe CCC	F	47	27
CCC-S15	Severe CCC	F	63	37
CCC-S16	Severe CCC	F	44	25
CCC-S17	Severe CCC	M	39	20
CCC-S18	Severe CCC	M	58	25
CCC-S19	Severe CCC	M	66	21
CCC-S20	Severe CCC	M	50	25
CCC-S21	Severe CCC	M	51	23
CCC-S22	Severe CCC	M	58	29
CCC-S23	Severe CCC	M	58	28
CCC-S24	Severe CCC	F	66	25
CCC-S25	Severe CCC	F	45	20
CCC-S26	Severe CCC	F	60	24
CCC-S27	Severe CCC	F	39	20
CCC-S28	Severe CCC	M	51	35
CCC-S29	Severe CCC	F	61	15
CCC-S30	Severe CCC	F	47	35
CCC-S31	Severe CCC	F	46	20
CCC-S32	Severe CCC	F	61	27
CCC-S33	Severe CCC	F	58	30
CCC-S34	Severe CCC	F	49	15
CCC-S35	Severe CCC	M	49	21
CCC-S36	Severe CCC	M	62	21
CCC-S37	Severe CCC	M	57	29
CCC-S38	Severe CCC	M	59	17
CCC-S39	Severe CCC	M	48	19
CCC-S40	Severe CCC	F	54	36
DCM-01	DCM	M	52	30
DCM-02	DCM	F	32	20
DCM-03	DCM	F	24	29
DCM-04	DCM	M	46	25
DCM-05	DCM	M	15	29
DCM-06	DCM	M	55	25
DCM-07	DCM	F	29	25
DCM-08	DCM	M	36	14
DCM-09	DCM	M	26	25
DCM-10	DCM	M	42	9
DCM-11	DCM			
DCM-12	DCM	F	57	27
DCM-13	DCM	M	48	39
DCM-14	DCM	F	66	20
DCM-15	DCM	M	43	26
DCM-16	DCM	M	53	19
DCM-17	DCM	M	39	28
DCM-18	DCM	M	58	21
DCM-19	DCM	F	12	22
DCM-20	DCM	F	56	18
DCM-21	DCM	M	29	26
DCM-22	DCM	M	61	27
DCM-23	DCM	M	15	20
DCM-24	DCM	F	58	17
DCM-25	DCM	M	28	16
DCM-26	DCM	M	56	16
DCM-27	DCM	M	27	25
DCM-28	DCM	F	53	27
DCM-29	DCM	M	51	15
DCM-30	DCM	M	56	35
DCM-31	DCM	M	37	16

### Reagents

Interferon-gamma (IFN-γ, 300-02) and tumor necrosis factor alpha (TNF-α, 300-01A) were purchased from Peprotech. AMPK agonists Metformin hydrochloride (PHR1084), AICAR (A9978), Resveratrol (R5010); SIRT1 agonist SRT1720 (SRT1720) and NRF-2/HO-1 agonist Protoporphyrin IX cobalt chloride (C1900) were purchased from Sigma-Aldrich. Inhibitors of NF-κB Emodin (E7881) and JSH23 (J4455); IKKβ inhibitor IKK16 (SML1138); STAT1 inhibitor Fludarabine (F2773) and NOS2 inhibitor 1400W (W4262) were purchased from Sigma-Aldrich. Inhibitors of MEK1 and MEK2 PD98059 (ab120234), JNK SP600125 (ab120065) and MAPK SB202190 (ab120638) were purchased from Abcam. Reagents were dissolved in DMSO or water (Metformin and AICAR) at 100x stock concentrations and maximum DMSO concentration used in cells was 1%. Monoclonal antibody against 3-nitrotyrosine (ab61392) was purchased from Abcam. Monoclonal antibody against NF-κB p65 (sc-8008) was purchased from Santa Cruz Biotechnology.

### Cell Culture

Human adult ventricular cardiomyocytes cell line AC-16 was kindly provided by Dr. Mercy Davidson (Columbia University, USA) (24). The cell line was propagated using Dulbecco’s modified Eagle’s medium/F-12 medium with 12.5% fetal bovine serum (FBS) without antibiotics for no longer than 8 passages. AC-16 cells were screened monthly for mycoplasma contamination (MycoAlert Mycoplasma Detection Kit, Lonza) and were authenticated by PCR-based DNA Profiling (Eurofins Genomics, France).

### Cell Stimuli and Investigation of Molecular Pathways

AC-16 cells were seeded (10^3^ cells per 0.34 cm²) for 24 hours (h) prior to stimulation with 10 ng/ml IFN-γ, or with 5 ng/ml TNF-α or with IFN-γ plus TNF-α for 48h. In certain experiments, cells were treated with serially diluted, by a factor of two, with agonists of AMPK (metformin, AICAR and resveratrol), NRF2 (Protoporphyrin IX cobalt chloride), SIRT1 (SRT1720) or inhibitors of NF-κB (Emodin, JSH23), IKKβ (IKK16), STAT1 (Fludarabine), NOS2 (1400W), MEK1 and MEK2 (PD98059), JNK (SP600125) and MAPK (SB202190) in combination or not with IFN-γ, TNF-α or both. Compound doses that provided the highest effect on mitochondrial ΔΨm and less than 10% loss on cell number were selected for further analysis.

### LDH Release Assay

The release of lactate dehydrogenase (LDH) in the conditioned media is an indicator of cell cytotoxicity. AC-16 cardiomyocytes were stimulated with the cytokines for 48h and the LDH content was quantified using a LDH-Cytotoxicity Assay Kit II (Abcam) using SpectraMax^®^ Paradigm^®^.

### Mitochondrial Membrane Potential and Cellular ROS

AC-16 cardiomyocytes were seeded in 96 well black plate (Corning 3603) at a density of 10^3^ cells per 0.34 cm² and incubated in a humidified incubator at 37°C and 5% CO_2_ for 24h. Then, cells were stimulated with the cytokines with or without the agonists/inhibitors for 48h. For mitochondrial membrane potential, cells were multi-labeled with 1.0 µM of tetramethylrhodamine, methyl ester, perchlorate (TMRM, ThermoFisher Scientific), 400 nM of MitoTracker DeepRed (ThermoFisher Scientific), NucGreen dead (ReadyProbes) and 1.0 µM of Hoechst 33342 (ThermoFisher Scientific) at 37°C 5% CO_2_ for 30 min. Cells were washed once with Hanks’ Balanced Salt solution containing calcium and magnesium (HBSS++). Micrographs were captured using ImageXpress Micro XLS Widefield High-Content Analysis system (Molecular Devices) at 100x magnification and the mitochondrial membrane potential was evaluated in live cells (NucGreen negative) using the web-based software Columbus 2.7.1.133403 (PerkinElmer Inc.). Overlapped TMRM and Mitotracker DeepRed fluorescence were considered for the measurement of mitochondrial ΔΨm. For cellular ROS, stimulated cells were washed once with HBSS++ and then labeled in the dark with 10 µM of H_2_DCFDA (Abcam) and 1.0 µM of Hoechst 33342 (ThermoFisher Scientific) at 37°C 5% CO_2_ for 30 min and fluorescence were measured at Ex/Em 485/535 nm and 360/461 nm respectively using SpectraMax^®^ Paradigm^®^.

### NF-κB Translocation

Stimulated AC-16 cells were fixed with 4% paraformaldehyde, pH=7.4 for 15 min at room temperature (RT), then incubated with ice-cold 0.5% Triton-X for 15 min and blocked with 3% BSA in PBS for 1h RT. Cells were incubated with 1:50 monoclonal mouse anti-NF-κB p65 (SC-8008, Santa Cruz Biotechnology) in PBS + 1% BSA overnight at 4°C, then washed and kept in the dark with goat anti-mouse IgG1 Cross-Adsorbed Secondary Antibody (P-21129, ThermoFisher Scientific) 1:1000 in PBS + 1% BSA for 1h RT. Nuclei were stained with 2.5 µg/ml of DAPI (ThermoFisher Scientific) for 1h RT and images were acquired using ImageXpress Micro XLS Widefield High-Content Analysis system (Molecular Devices) at 40x magnification. Nuclear NF-κB translocation was measured using the software Columbus 2.7.1.133403 (PerkinElmer Inc.).

### Assessment of 3-Nitrotyrosine

Relative quantification of nitrated protein was performed by assessing 3-nitrotyrosine (3-NT) as a mean to detect nitrosative stress. Cardiac fragments and stimulated AC-16 cells were lysed in aqueous lysis solution containing 10 mM HEPES, 0.32 M sucrose, 0.1 mM Na_2_EDTA, 1.0 mM dithiothreitol, 125 µg/ml PMSF and 1.0 µl/ml of proteinase inhibitor cocktail (Sigma), pH=7.4. Cardiac fragments were lysed in a tissue homogenizer (Precellys 24) pre-chilled 4°C using 2.8 mm ceramic beads for 3 cycles of 10s (seconds) with 15s intervals at 5500 rpm. Cells were lysed with 1.4 mm ceramic beads for 2 cycles of 30s with 10s intervals at 5000 rpm. Cardiac and cell lysates were clarified by centrifugation at 10,000 rcf for 30 min at 4°C. A total of 5 µg of proteins was added to a nitrocellulose membrane, dried at 60°C for 15 min and then blocked with 3% Blotting Grade Blocker (BioRad) + TBS-T, for 1h at RT, under stirring. Membrane was washed with TBS-T and incubated with 1:1000 primary monoclonal antibody 3-nitrotyrosine (Abcam) in 3% BSA + TBS-T overnight 4°C. Then, membrane was washed twice and incubated with 1:1000 secondary antibody for 2h at RT, under agitation and protected from light. Fluorescence was captured using a scanner (LI-COR, Odyssey) or ECL revelation. Ponceau S images were captured using ImageQuant LAS-400 (GE Healthcare) and used for protein normalization.

### ATP and Nitrite Production

Nitrite ( ${\text{NO}}_{2}^{-}$ ) was measured in the conditioned media (phenol-free) of stimulated AC-16 cells using Griess Reagent Kit for Nitrite Determination (Molecular Probes) according to manufacturer’s instructions. The cells were collected and lysed with TE buffer (100 mM Tris, 4 mM EDTA, pH=7.5) and ATP measured with a luciferase-based assay kit (ATP Determination Kit, ThermoFisher Scientific) according to manufacturer’s instructions. Nitrite absorbance and ATP-derived luminescence were measured in AC-16 cardiomyocytes using SpectraMax^®^ Paradigm^®^ (Molecular Devices). Measurement of nitrite in myocardial homogenates was performed by chemiluminescence with Nitric Oxide Analyzer (NOA 280, Zysense, United Kingdom).

### Bioenergetic Function Analysis

A Seahorse XFe24 Analyzer (Agilent, Les Ulis, France) was used to survey bioenergetic function by measuring the oxygen consumption rate (OCR) and extracellular acidification rate (ECAR) of live cells. A quantity of 3,500 cells/0.32cm^2^ were seeded in 150 µl of specific media, incubated for 1h at room temperature for adhesion and stimulated with 350 µl of specific media containing IFN-γ (10 ng/ml final) and TNF-α (5 ng/ml final) for 48h. OCR and ECAR were obtained from 90% confluent monolayer culture. All the experiments were done according to the protocol provided by the manufacturer. Briefly, the Agilent Seahorse Cell Mito Stress Test kit assesses mitochondrial function. Multiple parameters are obtained such as, basal respiration, ATP-linked respiration, maximal and reserve capacities, and non-mitochondrial respiration. The ATP Real-Time rate kit is the only assay that quantifies the ATP production from glycolysis and mitochondria simultaneously. The Agilent Seahorse Mito Fuel Flex Test kit measures the basal state of mitochondrial fuel oxidation in live cells by providing information on the relative contributions of glucose, glutamine and long-chain fatty acid oxidation to basal respiration. All analyses were performed using the software Wave 2.6.1 (Agilent, Les Ulis, France).

### mtDNA Quantification

Total DNA from cardiac fragments and AC-16 cells was extracted using the DNA extraction kit FlexiGene DNA kit (QIAgen) according to the manufacturer’s instructions. PCR reaction was conducted using MAXIMA SYBR Green/ROX qPCR Master Mix (2x) (Thermo Scientific), 1 ng of template DNA and primers for mitochondrial encoded genes NADH:ubiquinone oxidoreductase subunit 1 (MT-ND1, 5 pmol/µl), cytochrome b (MT-CYTB, 5 pmol/µl), cytochrome c oxidase subunit 3 (MT-COXIII, 5 pmol/µl). Nuclear gene Telomerase (TERT, 15 pmol/µl) was used as the endogenous control. The PCR reactions were done in QuantStudio 12K (Applied Biosystem) with the following cycling program, 1 cycle of 50°C for 2 min, 95°C for 10 min, and 40 cycles of 50°C for 2 min, 95°C for 15s, and 60°C for 1 min. The ratio of mtDNA/nuDNA was calculated using the formula 2x2^ΔCT^ (25).

### RNA Extraction and Sequencing

AC-16 cells were crushed with ceramic beads (VK05, diameter 1.4 mm) in 350 µl of RLT lysis buffer supplemented with 3.5 µl of β-mercapto-ethanol. Total RNA was extracted by using the RNeasy Mini Kit adapted with Trizol. RNA quantity was measured with a 2100 Bioanalyzer. Total RNA Sequencing was provided by Genewiz. Ribosomal RNAs have been depleted, and samples were prepared for sequencing according to the Illumina TruSeq RNA Preparation Kit and subjected to pairwise sequencing (2x150 bp) with the Illumina Novaseq sequencer. After sequencing, the raw data files (fastq) were generated.

### Differential Expression Analysis

Raw data quality has been verified with FastQC (v0.11.5). Low-quality reads, Illumina adapters and reads smaller than 20 nucleotides were removed with Trimmomatic (v0.39) (26), using default values for other options. Reads were aligned on GRCh37 (hg19) human reference genome from Ensembl using STAR (v2.5.4b) (27), specifying that the reads are 2x150 nucleotides (paired-end). Alignment quality has been checked by BAMQC (qualimap v2.2.1) (28). Gene quantification was done with featureCounts (v2.0.0) (29), using the exons as features and the genes as attributes. The DESeq2 package (v1.26.0) has been used to normalize data and to perform the differential expression test (30). To improve log2 fold change accuracy, DESeq2 shrinkage function has been applied to the test results, and p-value was corrected with Benjamini-Hochberg method. Genes having an adjusted p-value ≤ 0.05 and a |log2(FC)| ≥ 1.5 were considered to be differentially expressed genes (DEG). Then, results were analyzed by graphical methods with EnhancedVolcano (v1.4.0) and ggplot2 packages. Moreover, hierarchical clustering (HCA) based on spearman correlation was computed using gplots (v3.0.4) package, and principal component analysis (PCA) with factoextra (v1.0.7) and FactoMineR (v2.3) packages. We compared the AC-16 gene expression profile with the CCC gene expression profile (GSE84796) (31).

### Proteomic Analysis of AC-16 Cardiomyocytes

The proteomic analysis of AC-16 cells under IFN-γ (10 ng/ml) and TNF-α (5 ng/ml) treatment was assessed by high-resolution mass spectrometric analysis. After 48h incubation with the combination of IFN-γ and TNF-α, proteins from AC-16 were extracted using lysis buffer (12 mM sodium deoxycholate, 12 mM *N*-lauroylsarcosine sodium salt, 100 mM Tris-HCl, pH=9.0) supplemented with protease inhibitor cocktail (Sigma-Aldrich). Total cell lysates from control and IFN-γ+TNF-α-treated cells were subject to in-solution trypsin digestion according to Phase Transfer Surfactant (PTS)-aided trypsin digestion protocol (32). Digested proteins were desalted using StageTip protocol (33) and dried by centrifugation under vacuum. Peptide samples were analyzed by RP-LC-MS/MS. For MS analysis, peptide mixtures were dissolved in 0.1% formic acid (solution A). Aliquots of 3 μl were automatically injected by a nano chromatography EASY-nLCII system (Thermo Scientific) on a 40 mm x 100 μm ID C-18 pre-column packed with Jupiter 10 μm resin (Phenomenex) and submitted to a chromatographic separation in a 100 mm x 75 μm ID C-18 column packed with Reprosil-Pur 3 μm C-18 beads (Dr. Maisch) coupled to an LTQ Orbitrap Velos (Thermo Scientific). Peptides were eluted with a linear gradient of 5-30% solution B (0.1% formic acid in acetonitrile) at 200 nl/min for 55 min. Spray voltage was set at 2.5kV and the mass spectrometer was operated in data dependent mode, in which one full MS scan was acquired in the m/z range of 400-1,800 at 60,000 resolution (at 400m/z) followed by collisional induced fragmentation (CID) and MS/MS acquisition of the ten most intense ions from MS scan. Unassigned and +1 charge states were not subjected to fragmentation. The maximum injection time and AGC target were set to 100ms (milliseconds) and 1E6 for full MS, and 10ms and 1E4 for MS/MS. The minimum signal threshold to trigger fragmentation event, isolation window and normalized collision energy (NCE) were set to, respectively, 1000 cps, 2m/z and 35%. Dynamic peak exclusion (list size of 500) was applied to avoid the same m/z of being selected for the next 30s. Mass spectrometric raw data were processed using the software MaxQuant [version 1.6.2.10 (34)] and searched against a target human protein database (*Homo sapiens*, UniprotKB reviewed, 26,526 sequences) for protein identification and quantification. A False Discovery Rate (FDR) of 1% was required for both protein and peptide identifications. Label Free Quantification [MaxLFQ (35)] method was enabled. T-test was used for relative protein quantification and p-values were adjusted with the Benjamini-Hochberg method. Proteins with adjusted p-values ≤ 0.05 were considered to be differentially expressed.

### Statistical Analysis

Cell data are reported as the ratio to non-treated cells. Cell viability was calculated as the ratio of the number of live cells (NucGreen-negative) and total cells (NucGreen-negative plus NucGreen-positive cells) x100. The results were expressed as mean ± standard deviation (SD) or standard error of the mean (SEM) when noted. Statistical analysis was performed using the GraphPad Prism 9.2.0 software (GraphPad Software, Inc., CA). Data were tested for normality using the Shapiro-Wilk test before statistical tests. A nonparametric Mann-Whitney test was applied if data were not normally distributed. In all other cases, one-way ANOVA followed by Dunn’s *post hoc* test was applied for multiple comparisons. A p-value < 0.05 was considered statistically significant.

## Results

### Increased RNS and Decreased mtDNA Content in CCC Myocardial Tissue

**Figure 1:**
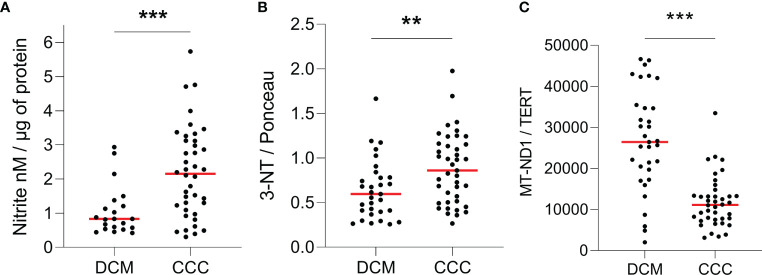
Quantification of ROS, RNS and mitochondrial DNA in heart tissue. Lysates of left ventricular heart tissue were obtained from CCC (n = 40) and DCM (n = 31) patients and used for the quantification of **(A)** nitrite (nM) by chemiluminescence assay; **(B)** 3-nitrotyrosine by dot-blot and **(C)** copy number of the mitochondrial gene MT-ND1 by real time PCR. Red line: median; **p < 0.01; ***p < 0.001; Mann-Whitney test.

We compared the nitro-oxidative profile of CCC and DCM myocardium. We first measured nitrite, a reactive nitrogen species (RNS) marker, by chemiluminescence with a NO Analyzer. We observed that CCC biopsies possess higher content of nitrite (132%; p < 0.001) in comparison to DCM lysates (**Figure 1A**). Detection of nitrotyrosine, an additional nitro-oxidative stress marker, was performed by dot-blot. We observed a significantly higher nitrotyrosine immunoreactivity in CCC (27%; p < 0.01) than DCM myocardial lysates (**Figure 1B**), indicating previous exposure to peroxynitrite. Also, mitochondrial DNA (MT-ND1) was lower in CCC (44%; p < 0.001) than DCM myocardial samples (**Figure 1C**) indicating a reduction of mitochondrial mass.

### IFN-γ and TNF-α Impair AC-16 Cardiomyocyte Mitochondrial Membrane Potential (ΔΨm)

**Figure 2:**
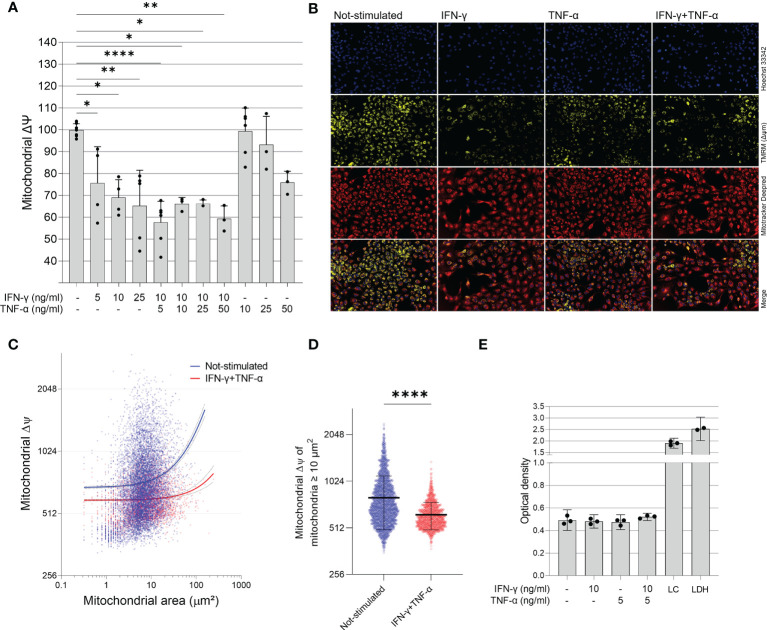
Effect of IFN-γ and TNF-α on mitochondrial membrane potential in human cardiomyocytes AC-16 cells. Stimulated cells were multi-labelled with TMRM, Mitotracker DeepRed, NucGreen and DAPI. TMRM fluorescence was measured when colocalized with the fluorescence of Mitotracker Deep Red in live cells (NucGreen-negative). **(A)** IFN-γ and IFN-γ plus TNF-α downmodulate ΔΨm 48h after stimulation. Data shown as percentage to not-stimulated cells. *p < 0.05; **p < 0.01; ****p < 0.0001; One-way Anova, Dunn’s post test. **(B)** Representative micrographs of the effect of IFN-γ (10 ng/ml), TNF-α (5 ng/ml) and both on the fluorescence of TMRM. Magnification 100x. **(C)** Correlation between size (µm^2^) and TMRM fluorescence intensity of segmented mitochondria of cells stimulated with 10 ng/ml of IFN-γ and 5 ng/ml of TNF-α. **(D)** ΔΨm of mitochondria larger ≥ 10 µm^2^ of cells stimulated with 10 ng/ml of IFN-γ and 5 ng/ml of TNF-α. ****p < 0.0001 Mann-Whitney test. **(E)** Supernatant quantification of lactate dehydrogenase (LDH assay) on stimulated cells. Each dot in bar graphs represents an independent experimental replicate n≥3. LC: lysed AC-16 cells; LDH: lactate dehydrogenase positive control.

To investigate the role of IFN-γ and TNF-α on AC-16 cardiomyocytes, we stimulated the cells with several concentrations of IFN-γ and TNF-α and we measured the mitochondrial ΔΨm in a high content screening platform. This was performed to stablish the concentrations of the cytokines for subsequent analyses. We observed that IFN-γ impaired the ΔΨm of AC-16 48h after stimulation and this impairment was enhanced when IFN-γ was combined with TNF-α; TNF-α alone failed to cause statistically significant reductions in ΔΨm (**Figures 2A, B**). We then selected the concentrations of 10 ng/ml of IFN-γ and 5 ng/ml of TNF-α and we detected that IFN-γ and TNF-α decrease ΔΨm of mitochondria larger than 10 µm^2^ (**Figures 2C, D**) and the impact is higher on larger mitochondria (**Supplementary Figure 1**). We performed the LDH assay of the selected doses of IFN-γ (10 ng/ml) and TNF-α (5 ng/ml) and we observed no cytotoxicity (**Figure 2E**).

### Cytokine-Stimulated AC-16 Cells Display Increased RNS and Decreased ATP and mtDNA

**Figure 3:**
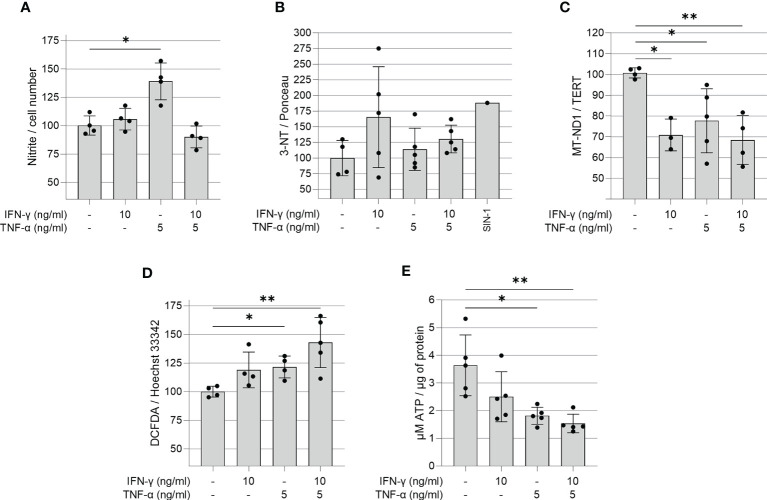
Quantification of ROS, RNS, ATP and mitochondrial DNA in IFN-γ and TNF-α stimulated AC-16 cells. Human cardiomyocytes AC-16 cells were stimulated with 10 ng/ml of IFN-γ, 5 ng/ml of TNF-α or both for 48h. Then, cell lysates were used for the quantification of **(A)** nitrite (µM) by Griess Reaction; **(B)** 3-nitrotyrosine by dot blot; **(C)** copy number of the mitochondrial gene MT-ND1 by real time PCR; **(D)** cellular reactive oxygen species (ROS) by fluorescence assay using the probe DCFDA and **(E)** ATP by luciferase-based assay. Data are shown as percentage to not-stimulated cells in **(A–D)** *p < 0.05; **p < 0.01; one-way Anova Dunn’s post test. Each dot represents an independent experiment n≥3.

We investigated whether IFN-γ and TNF-α prompt nitrosative and oxidative stress in AC-16 cardiomyocytes. We used the Griess reaction to measure nitrite in the supernatant of 48h IFN-γ and TNF-α-stimulated AC-16 cells. Nitrite accumulation in the supernatant of AC-16 was increased by TNF-α alone and not by IFN-γ or their combination after 48h of incubation (**Figure 3A**). Detection of 3-NT was performed by dot-blot. We observed that although IFN-γ or the cytokine combination induced a median increase of 25-72% in protein nitration, this was not statistically significant, most likely due to the high dispersion (**Figure 3B**). Cytokine treatment of AC-16 cardiomyocytes with IFN-γ, TNF-α and combined for 48h reduced the amount of MT-ND1 as compared to non-stimulated cells (**Figure 3C**). We also measured reactive oxygen species (ROS) production in AC-16 stimulated cells stained with the fluorogenic dye H_2_DCFDA and we found a 43% increase in ROS after stimulation with IFN-γ and TNF-α and 23% with TNF-α (**Figure 3D**). In addition, luminescence assay also showed that the TNF-α alone or combined with IFN-γ decreased 50% and 58%, respectively, the ATP content in AC-16 cardiomyocytes (**Figure 3E**).

### Molecular Pathways Analysis of Cytokine-Induced Δψm Reduction

**Figure 4:**
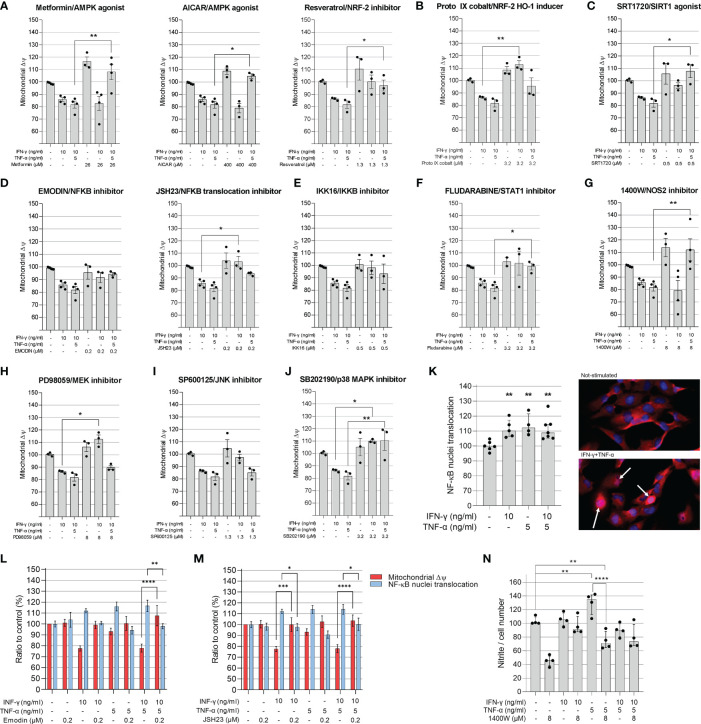
Evaluation of compounds in the mitochondrial membrane potential of AC-16 cells. Selected doses of agonists of AMPK **(A)**, NRF2 **(B)**, SIRT1 **(C)** or inhibitors of NF-κB **(D)**, IKKβ **(E)**, STAT1 **(F)**, NOS2 **(G)**, MEK1 and MEK2 **(H)**, JNK **(I)** and MAPK **(J)** were used alone or in combination with 10 ng/ml of IFN-γ or IFN-γ plus 5 ng/ml of TNF-α. Specific doses were selected based on the highest effect on mitochondrial ΔΨm and less than 10% loss on cell number. **(K)** The nuclear translocation of NF-κB in AC-16 cells was quantified by immunocytochemistry. Simultaneous measurement of ΔΨm and NF-κB translocation of cells stimulated with 10 ng/ml of IFN-γ or IFN-γ plus 5 ng/ml of TNF-α in combination with the NF-κB inhibitors **(L)** emodin and **(M)** JSH23. Statistics shown only when both ΔΨm and NF-κB translocation were significant. **(N)** the amount of nitrite (µM) in conditioned medium was measured by Griess reaction of cells stimulated with 10 ng/ml of IFN-γ or IFN-γ plus 5 ng/ml of TNF-α in combination with NOS2 inhibitor 1400W. Micrographs of NF-κB stains in the nucleus (white arrow). All data are shown as percentage to not-stimulated cells. Standard deviation is from ≥3 independent experiments. *p < 0.05; **p < 0.01; ****p < 0.0001 one-way Anova Dunn’s post test.

In order to better understand the mechanisms by which IFN-γ and TNF-α diminish AC-16 ΔΨm, cells were treated with several compounds that activate or inhibit specific pathways. Each drug was titrated and the concentration of each compound was selected based on the highest restorative effect on ΔΨm and no more than 10% loss on cell number (**Supplementary Figures 2**, **3**). Concurrent treatment showed that agonists of signaling pathways related to response to stress and mitochondrial protection, such as AMPK (metformin, AICAR, resveratrol), NRF2 (Resveratrol and Protoporphyrin XI) and SIRT1 (SRT1720) restore ΔΨm (**Figures 4A–C**). Additionally, inhibition of IFN-γ downstream molecules with selected antagonists such as fludarabine (STAT1 inhibitor), emodin (NF-κB activation) JSH23 (NF-κB nuclear translocation), IKK16 (IκB kinase) and NOS2 (1400W) also restores AC-16 ΔΨm (**Figures 4D–G**). Finally, inhibition of TNF-α-downstream molecules such as MEK (PD98059), JNK (SP600125) and MAPK (SB202190) also restores AC-16 ΔΨm (**Figures 4H–J** respectively). The effect of NF-κB inhibitors on NF-κB nuclear translocation was validated by additional experiments. Immunocytochemistry shows that IFN-γ and TNF-α promote NF-κB nuclear translocation (**Figure 4K**), which was antagonized with emodin and JSH23 and simultaneously restored ΔΨm in AC-16 cardiomyocytes (**Figures 4L–M**). In addition, **Figure 4N** shows that NOS inhibitor 1400W significantly decreased nitrite accumulation caused by TNF-α (p < 0.001). A schematic representation of the signaling pathways and the targets of the compounds are detailed in **Supplementary Figure 4**.

### IFN-γ+TNF-α Costimulation on the AC-16 Cell Line Induces a Significant Decrease of Total, Glycolysis and Mitochondrial ATP Production

**Figure 5:**
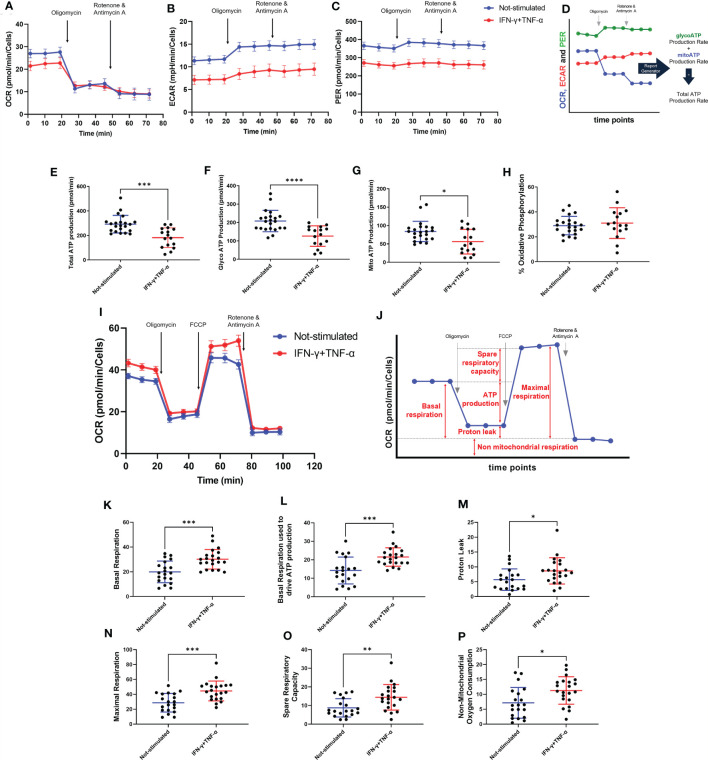
Assessment of AC-16 respiration and ATP production in response to IFN-γ and TNF-α stimulation. Oxygen consumption rate (OCR), extracellular acidification rate (ECAR) and proton Efflux Rate (PER) of 48h IFN-γ (10 ng/ml) and TNF-α (5 ng/ml) -stimulated AC-16 cells were obtained in Seahorse XFe24 Analyzer. Arrows show injection of inhibitors of ATP synthase (oligomycin), mitochondrial oxidative phosphorylation uncoupler (FCCP) and OXPHOS complex I and III inhibitors (Rotenone and Antimycin A). **(A–C)** OCR, ECAR and PER measurement respectively obtained from ATP rate assay. **(D)** Calculation of mitochondrial and glycolytic ATP. **(E–G)** Quantification of total, glycolytic and mitochondrial ATP. **(H)** Percentage of oxidative phosphorylation. **(I)** OCR measurement of not-stimulated and cytokines-stimulated cells in the Mitostress test. **(J)** Representative graph showing the different parameters evaluated, such as **(K)** basal respiration, **(L)** basal respiration used to drive ATP production, **(M)** proton leak, **(N)** maximal respiration, **(O)** spare respiratory capacity and **(P)** non-mitochondrial oxygen consumption. Standard error of the mean in line graphs. Standard deviation is from ≥16 independent measurements. *p < 0.05; **p < 0.01; ***p < 0.001; ****p < 0.0001 Mann-Whitney test.

The effect of IFN-γ+TNF-α stimulation on AC-16 was investigated by measuring the two major production pathways in mammalian cells (glycolysis and oxidative phosphorylation). Twenty-two independent measurements were done on non-stimulated AC-16 cells and 16 independent measurements were done on IFN-γ+TNF-α stimulated AC-16 cells. The OCR, ECAR and PER values were used to calculate glycolytic and mitochondrial ATP (**Figures 5A–D**). IFN-γ+TNF-α induced a significant decrease of total ATP production (33%; p=0.0001) (**Figure 5E**), glycolysis ATP production (30%; p<0.0001) (**Figure 5F**), and the mitochondrial ATP production (41%; p=0.02) (**Figure 5G**) was in line with the previous finding (**Figure 3E**). The percentage of oxidative phosphorylation was unchanged (**Figure 5H**).

### IFN-γ+TNF-α Costimulation on the AC-16 Cell Line Induces an Increase of the Basal Respiration, Maximal Respiration, and Spare Respiratory Capacity

Metabolic flux analyses were performed on AC-16 cells with or without cytokine stimulation during 48h (n=21) (**Figure 5I**). **Figure 5J** illustrates the parameters analyzed. Cytokine-stimulated AC-16 cardiomyocytes increased basal respiration (53%; p=0.0007) compared to unstimulated cells (n=20) (**Figure 5K**). Also, the cytokines increased 54% (p=0.0009) the basal respiration used to drive ATP production (**Figure 5L**). Cytokine stimulation for 48h induces a proton leak (51%) indicating alteration of the mitochondria integrity (p=0.021) (**Figure 5M**). We also identified that cytokine stimulation increases the maximal respiration (44% higher) compared with unstimulated cells (p=0.0005) (**Figure 5N**). Similar results are obtained for the spare respiratory capacity, which is the difference between maximal respiration and basal respiration (p=0.005) (**Figure 5O**). Cytokine stimulation also induced an increase in non-mitochondrial respiration (74%) compared to the non-mitochondrial respiration detected on unstimulated cells (p=0.014) (**Figure 5P**).

### IFN-γ+TNF-α Costimulation Decreases AC-16 Cells’ Dependency of Fatty Acid and Glutamine Oxidation

We investigated the effect of IFN-γ+TNF-α on AC-16 cell’s dependency and capacity of fuel oxidation using Seahorse. We measured oxygen consumption after injection of different inhibitors (combined or not), such as: UK5099, inhibitor of the mitochondrial pyruvate carrier (MPC, glucose oxidation), BPTES, inhibitor of glutaminase GLS1 (KGA, glutamine oxidation) and etomoxir, an inhibitor of carnitine palmitoyltransferase-1 (CPT-1, fatty acid oxidation). We then calculated the dependency, which is the cell’s reliance on a particular fuel pathway to maintain basal respiration, and capacity, i.e. the ability of mitochondria to oxidize a fuel when other fuel pathways are inhibited.

**Figure 6:**
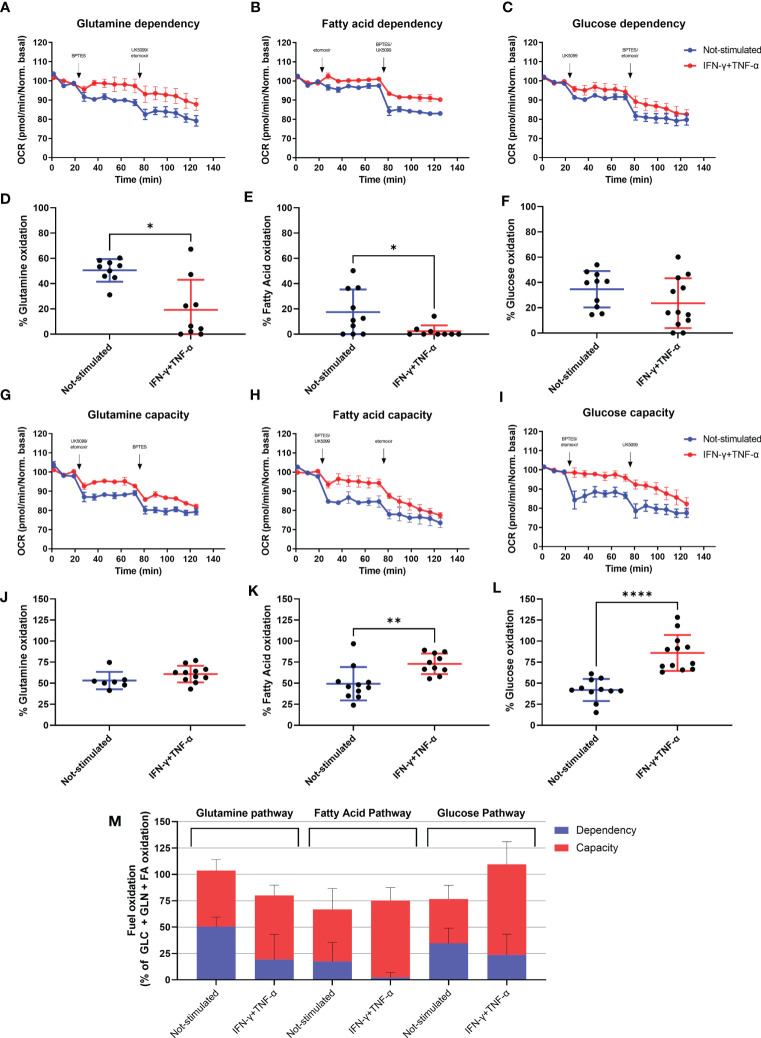
Assessment of AC-16 fuel oxidation in response to IFN-γ and TNF-α stimulation. Oxygen consumption rate of 48h IFN-γ (10 ng/ml) and TNF-α (5 ng/ml)-stimulated AC-16 cells was obtained in Seahorse XFe24 Analyzer. Arrows show injection of inhibitors (combined or not) of molecules specific to different fuel oxidation pathways, such as; BPTES, inhibitor of glutaminase GLS1 (KGA, glutamine pathway), etomoxir, inhibitor of carnitine palmitoyltransferase-1 (CPT-1, fatty acid pathway) and UK5099, inhibitor of the mitochondrial pyruvate carrier (MPC, glucose pathway). **(A–F)** OCR dependency of glutamine, fatty acid oxidation and glucose. **(G–L)** OCR capacity of glutamine, fatty acid oxidation and glucose. **(M)** Global fuel oxidation. Standard error of the mean in line graphs. Standard deviation is from ≥9 independent measurements. *p < 0.05; **p < 0.01; ****p < 0.0001 Mann-Whitney test.

We found that 48h stimulation with IFN-γ+TNF-α decreased 62% (p=0.0104 n=9) the reliance of AC-16 cells on glutamine (**Figures 6A, D**) and 87% dependency of fatty acid oxidation (p=0.0411 n≥9) (**Figures 6B, E**), with no effect on glucose dependency (**Figures 6C, F**). In addition, cells’ capacity to oxidize fatty acid and glucose increased 46% (p=0.0037 n≥10 **Figures 6H, K**) and 105% (p<0.0001 n≥11 **Figures 6I, L**) respectively after stimulation with the cytokines. No changes were detected in glutamine capacity (**Figures 6G, J**). A summary of the global fuel oxidation of not-stimulated and cytokine-stimulated cells is shown in **Figure 6M**.

### Mitochondrial Proteins Are Differentially Expressed Both in CCC and Cytokine-Treated AC-16 Cardiomyocytes

Extracted proteins from AC-16 were submitted to LC-MS/MS and comparative quantitative analysis of 1,211 identified proteins resulted in 275 differentially expressed proteins (DEP) between IFN-γ+TNF-α-treated AC-16 and non-treated AC-16; 37 DEPs were mitochondrial proteins, 25 being down-modulated and 12 up-regulated (**Supplementary Table 1**). Among downregulated proteins, 6 belonged to the TCA cycle and OXPHOS (ACO2, DLAT, FH, CS, NDUFV2, NDUFS8). We found reduced expression of two mitochondrial protein import enzymes (TIMM23, TOMM22) two linked to protein synthesis (RARS, GRSF1) and 2 to maintenance of mitochondrial membrane function and polarization (TRAP1, IMMT) and reduced levels of the ion carriers SLC25A40, SLC25A11, SLC25A3. We found downregulated proteins belonging to the lipid beta oxidation (ACOT13, HADH, FASN, ACAT1) and involved in ATP metabolism, ATP synthase regulation and ATP transport to the cytoplasm (ATPIF1, USMG5, PPIF and SLC25A6). PRDX3 and PRDX5 were downregulated and SOD2 was upregulated, consistent with oxidative stress. Reactome analysis of differentially expressed mitochondrial proteins disclosed repressed mitochondrial beta-oxidation and TCA cycle, increased glycolysis, increased mitochondrial protein import, ketone body metabolism and detoxification of reactive oxygen species. IPA canonical pathways analysis indicated mitochondrial dysfunction, reduced sirtuin-1 signaling, reduced TCA cycle, downregulated ketolysis, upregulation of superoxide radical degradation. A comparison of DEPs both in the cytokine-stimulated AC-16 cardiomyocytes and CCC myocardium (data not shown; submitted for publication) identified 19 matching proteins. Twelve out of the 19 were mitochondrial or related to energy metabolism, and 8 of them were similarly modulated in both proteome datasets. Among matching and differentially expressed mitochondrial proteins, we observed reduced expression of proteins including HADH and ACAT1 (fatty acid beta-oxidation), NDUFV2 (OXPHOS), and FH (TCA), with increased expression of PKM (glycolysis) and LAP3 (apoptosis).

### Mitochondrial Genes Are Differentially Expressed in Cytokine-Treated AC-16 Cardiomyocytes

**Figure 7:**
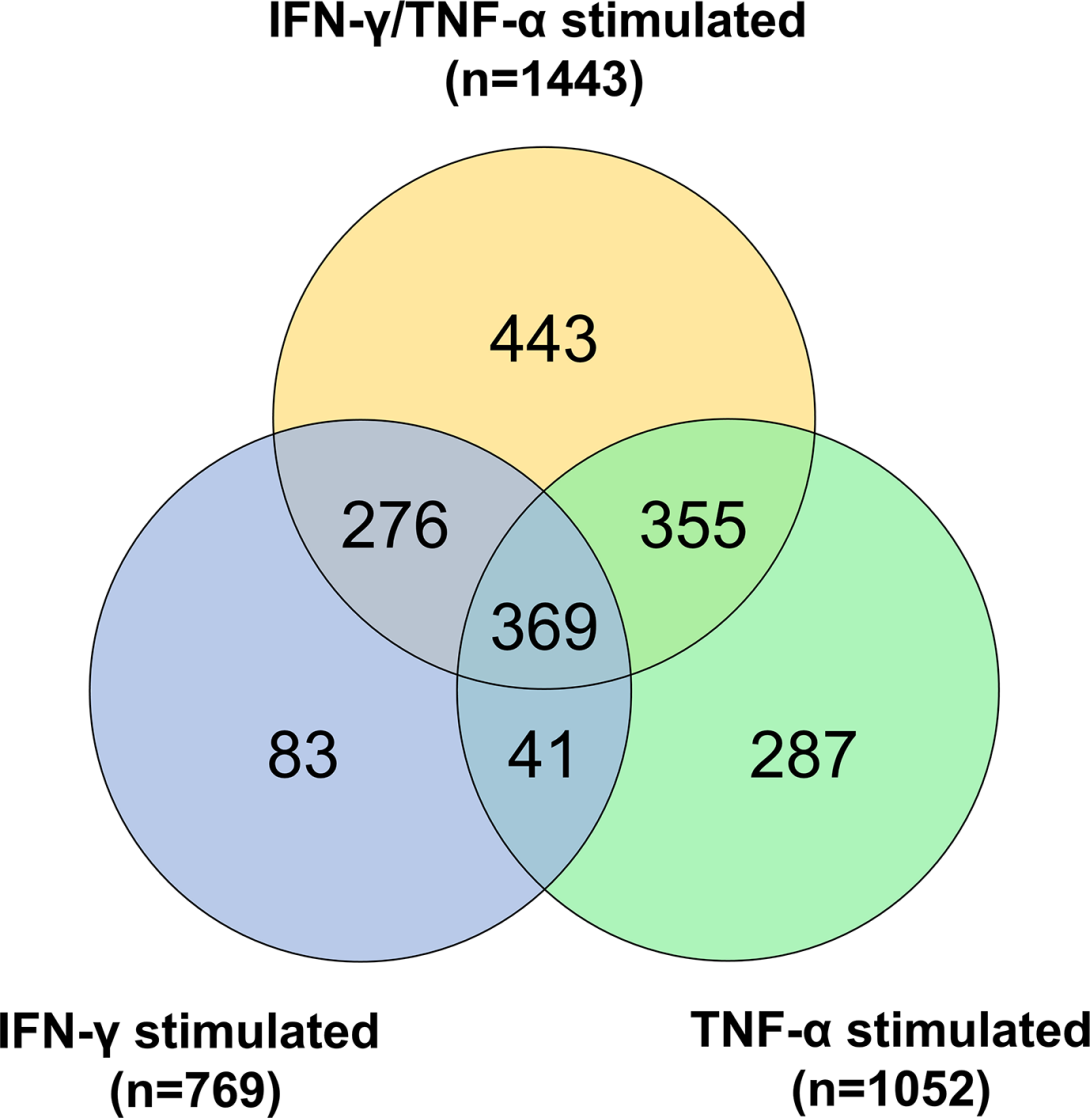
Gene expression analysis on AC-16 cardiomyocytes. Cells were stimulated with IFN-γ (10 ng/ml) or TNF-α (5 ng/ml) or IFN-γ + TNF-α during 1 or 6 or 12 or 24 or 48h. On cardiomyocytes stimulated with TNF-α 1052 DEGs were detected whereas on cardiomyocytes stimulated with IFN-γ 769 DEGs were detected. Finally, on cardiomyocytes stimulated 48h with IFN-γ + TNF-α 1443 DEGs were detected. Venn diagram describes the DEGs shared by the various stimulations. Each stimulation was performed 4 times (4n).

The AC-16 cell line was stimulated with IFN-γ and TNF-α during 1h to 48h. Gene expression analysis was performed in the various time points taking as reference the T=0h time point. We considered differentially expressed genes, genes characterized by a corrected p-value<0.05 (Benjamini Hochberg) and absolute log2 Fold Change>1.5. A total of 1443 DEGs were detected in all these comparisons (**Supplementary Table 2**). These DEGs include mainly protein coding genes (n=1149, 79.6%), as well as pseudogenes (n=68, 4.7%), antisense (n=89, 6.2%) and lnRNAs (n=94, 6.5%). The largest number of DEGs were detected at 6h or 12h (T=1h: n=214; T=6h: n=858; T=12h: n=847; T=24h: n=588; T=48h: n=234). Volcano plots and heatmaps are described in **Supplementary Figure 5** and **Supplementary Table 3**. The main pathways are linked to the immune response, to inflammation, to intracellular defense mechanisms, to fibrosis and cardiac diseases. Just after 1h of cytokine stimulation AC-16 cells increased the expression of cytokines and chemokines such as CXCL1, CCL2, CXCL2 and IL8, IRF1 and IRF9, indicating an interferon and pro-inflammatory signaling cascade with an overexpression of TNFAIP3 and NFKBIA which act as NFKB inhibitors. After 6h of stimulation, a large number of cytokine and chemokine genes and their receptors was upregulated, including CCL2, CCL7, CCL8, CCL13, CX3CL1, CXCL1, CXCL2, CXCL3, CXCL5, CXCL9, CXCL10, CXCL11, IFNAR2, IFNGR2, STAT1, TNF, IL1A, IL1B, IL4R, IL6, IL7, IL7R, IL12A, IL15, IL15RA, IL20RB, IL21R, IL32, IL33 genes). We also performed the same experiments with IFN-γ or TNF-α alone (**Figure 7** and **Supplementary Table 4**). Overall, we found 67 mitochondrial genes to be differentially expressed across at least one of the time points in response to combined cytokine stimulation. Differentially expressed mitochondrial genes peaked at 12h (46 genes) of which 25 were upregulated and 21 down-regulated (**Supplementary Table 5**). Among the top 10 canonical pathways of mitochondrial DEGs at 12h stimulus, we observed inhibition of the sirtuin signaling pathway, activation of aryl hydrocarbon receptor signaling, interferon signaling, and mitochondrial dysfunction. Toxicity-function pathways analysis at 12h indicated decreased transmembrane potential of mitochondria, fatty acid metabolism, mitochondrial dysfunction, pro-apoptosis, cardiac necrosis/cell death, cardiac hypertrophy among others (**Supplementary Table 6**). Mitochondrial dysfunction and decreased transmembrane potential were enriched toxicity functions in all time points starting at 6h (data not shown).

## Discussion

In this study, we investigated the mitochondrial function, nitro-oxidative profile, and gene and protein expression of myocardial samples from CCC patients. We also surveyed the effect of IFN-γ and TNF-α in AC-16 cardiomyocytes’ mitochondria. These cytokines are known to be selectively produced by the myocardium of CCC patients and not DCM (7–11, 36, 37). We have identified that CCC myocardium displays an increased nitro-oxidative stress profile, as well as reduced mtDNA content in comparison to DCM samples and that these phenomena were also observed by IFN-γ/TNF-α treatment of AC-16 cardiomyocyte cell line. We performed mechanistic studies to better understand the role of the multiple signaling pathways in the mitochondrial function of IFN-γ/TNF-α-treated AC-16 cardiomyocytes. We have found that the inhibition of STAT1/NF-κB/NOS2 axis and activation of AMPK, NRF2 and SIRT1 signaling pathways promoted protective effects in the IFN-γ/TNF-α-induced impairment of mitochondrial ΔΨ. Pathways analysis of gene and protein expression involved mitochondrial dysfunction and decreased ΔΨm were found in cytokine-treated AC-16 cells. In addition, we observed a cytokine-induced reduction in ATP production at the expense of mitochondrial energy metabolism in AC-16 cardiomyocytes that paralleled that observed in CCC myocardial tissue.

IFN-γ, but not TNF-α alone, was shown to impair the mitochondrial ΔΨm of AC-16 cells. We also observed that concurrent treatment with IFN-γ and TNF-α results in a further decrease in ΔΨm, and this reduction is higher in larger mitochondria. This finding is especially crucial since the mitochondrial ΔΨm is the OXPHOS proton motive force that drives ATP production through the ATP synthesis complex and thus it is an essential mechanism for contraction and survival of cardiac cells (38). Several *in vitro* studies have found the suppressing effect of TNF-α (39–42) and IFN-γ plus TNF-α (43) in mitochondrial ΔΨm in different cell types.

IFN-γ exerts its deleterious effects in the mitochondria at least partially by potentiating TNF-α-mediated NF-κB signaling (44). Activation of NF-κB triggers transcriptional activity of NOS2, which in turn produces nitric oxide (NO) and in the presence of reactive oxygen species (ROS) produces peroxynitrite (ONOO^-^) one on the most toxic and highly oxidative reactive species with substantial effects in mitochondria (45, 46). In our observations, IFN-γ and/or TNF-α can increase NF-κB nuclear translocation, nitrite and ROS production, and 3-nitrotyrosine accumulation in AC-16 cardiomyocytes. Peroxynitrite and protein nitration cause inactivation of enzymes, poly(ADP-ribosylation), mitochondrial dysfunction, impaired stress signaling and also potently inhibits myofibrillar creatine kinase (MM-CK), an important controller of heart contractility (47, 48) whose activity has been shown to be decreased in CCC (18). Increases in ROS by IFN-γ and/or TNF-α was also reported in several cell lines (39, 41, 49, 50), including cardiomyocytes (42, 51–53). Enhanced ROS production is associated with increased levels of lipid peroxidation, decreased mtDNA copy number and impaired OXPHOS capacity, affecting cardiomyocyte structure and function which triggers signaling pathways involved in myocardial remodeling and failure (54, 55). Enhanced oxidative stress has been observed in CCC heart tissue as measured by the accumulation of malondialdehyde (submitted for publication). We here reported reduction of mtDNA content in CCC myocardium and IFN-γ/TNF-α-stimulated cardiomyocytes. The mtDNA is a circular double stranded DNA located in the mitochondrial matrix and codes for 37 genes (56) and the 13 polypeptides are the essential subunits of the OXPHOS complexes I, III, IV, and V (56, 57). Deficiency in mtDNA replication was shown to cause ROS accumulation and oxidative stress in murine cardiomyocytes (58, 59), which is also indicative of a reduced mitochondrial mass. Thus, our data suggest that mtDNA reduction observed in the CCC myocardium might be linked to the oxidative stress observed in IFN-γ and TNF-α-treated human cardiomyocytes. This is corroborated by the gene and protein expression analysis in cytokine-treated AC-16 cardiomyocytes, where decreased levels of proteins involved in ATP generation mitochondrial protein and ion import and mitochondrial structural maintenance proteins and upregulated expression of proteins involved in ATP catabolism and mitochondrial transition pore. In addition, we observed pathways analysis of CCC heart tissue indicative of mitochondrial dysfunction, increased oxidative stress, and cardiac necrosis, all pointing towards mitochondrial stress and reduced functional capacity. In line with these, stimulation with IFN-γ and TNF-α depleted ATP production in AC-16 cells. Previous studies described the dampening of energy metabolism enzymes in CCC heart tissue, such as ATP synthase α and creatine kinase activity in patients (18–20, 60) and studies with animal models correlated with this outcome (61). Decreased *in vivo* ATP production was also observed in the CCC myocardium (21). In addition, our group identified an accumulation of heterozygous pathogenic variations including loss-of-function and stopgain/truncation of key mitochondrial genes in CCC patients (62). The loss of function mutation in one of the studied families was dihydroorotate dehydrogenase (DHODH) R135C. DHODH is involved in the oxidative phosphorylation by donating electrons to complex III and treatment with DHODH inhibitor Brequinar in IFN-γ+TNF-α-treated AC-16 cardiomyocytes caused additive damage to mitochondrial ΔΨm (62).

The inhibition of IFN-γ and TNF-α downstream molecules and pathways - STAT1/NF-κB, NOS2 - was important for the restoration of AC-16 ΔΨm and reduction of nitrite levels in AC-16 cardiomyocytes. The inflammatory milieu (IFN-γ, TNF-α, and IL-1b) enhanced ROS production in *T. cruzi* infected cardiomyocytes (51). Also, ROS production directly signaled the nuclear translocation of RelA (p65), NF-κB activation in AC-16 cells (63), indicating a positive feedback loop of stimulation between oxidative stress and NF-κB signaling. Studies reported that long-term sustained increase in ROS and RNS promotes cardiomyocyte dysfunction and apoptosis (64) resulting in reduction of mitochondrial ΔΨm, lipid beta-oxidation (65) and ATP generation (66, 67).

We found that the treatment of AC-16 cells with agonists of AMPK (resveratrol, AICAR and metformin) and NRF2 (protoporphyrin IX cobalt and resveratrol) rescued ΔΨm. AMPK and NRF2 are involved in the cellular response to oxidative stress by countering the damaging effects of NF-κB and by promoting ATP production and regulation of important physiological processes to restore heart function, such as autophagy (68–72). Our data showed that these agonists ameliorate IFN-γ/TNF-α-damaging effect to cardiomyocytes ΔΨm. Similarly, activation of AMPK (metformin) was shown to inhibit the enhancing effect of IFN-γ on the DOX‐induced cardiotoxicity and prolonged the survival time in DOX‐treated mice (65). Indeed, a recent study showed that antioxidants such as resveratrol and mitochondria-targeted antioxidants have potential benefits for the control of oxidative stress in the myocardium of mice with experimental Chagas disease cardiomyopathy (73). Our work potentially found the mechanistic link for the findings that treatment of chronically *T. cruzi*-infected mice with SIRT1 and/or AMPK agonists SRT1720, resveratrol and metformin or antioxidants reduced myocardial NF-κB transcriptional activity, inflammation and oxidative stress, resulting in beneficial results for restoration of cardiac function (73, 74).

Our transcriptomic profiling on cytokine-stimulated AC-16 cardiomyocytes over time (0 to 48h) showed that a cardiomyocyte can respond to inflammatory stimuli by producing inflammatory cell-attracting chemokines and inflammatory cytokines on its own as early as 1h after stimulus, perpetuating inflammation. Previous studies by our group in a subset of 10 out of the 30 CCC samples studied here observed increased mRNA expression of multiple chemokines and cytokines (9, 36, 75) in CCC heart tissue. At each time point, several genes associated with the mitogen-activated protein kinase (MAPK) signaling pathway were differentially expressed. This pathway is also one of the main inducers of the NF-κB pathway that is activated after inflammatory stimulus, ischemia/reperfusion, in congestive heart failure, dilated cardiomyopathy, after ischemic and pharmacological preconditioning, and in hypertrophy of isolated cardiomyocytes (76). Pathway analyses of gene and protein expression in the IFN-γ and TNF-α stimulated AC-16 cardiomyocytes profile were consistent with disturbances of ATP production and increased levels of reactive oxygen species. These metabolic changes affect cardiac ion channel gating, electrical conduction, intracellular calcium handling, and fibrosis formation.

As shown in the functional energy metabolism experiments, the integrity of the mitochondria is affected (proton leak). Therefore, cytokine-treated cardiomyocytes significantly increased their respiration (basal respiration, maximal respiration, spare respiratory capacity, as well as the percentage of basal respiration used to produce ATP. However, as mitochondria are less numerous (as seen with the reduced mitochondrial DNA) and their integrity is affected, the production of mitochondrial and glycolytic ATP is decreased in association with a significant decrease of the glutamine and fatty acid oxidation dependency – a reduction in metabolic flexibility that is found in failing hearts (77). This is in line with the IFN-γ and TNF-α induced NF-κB activation and activation of expression of NADP oxidases, and inducible nitric oxide synthase (NOS2), leading to the production of large amounts of NO and reactive nitrogen species (12, 78, 79) leading to synthesis of the peroxynitrite anion (ONOO^−^) production, a strong oxidant arising from the reaction of NO with superoxide radical ( ${\text{O}}_{2}^{-}$ ) (46).

This study has limitations, since we studied frozen human heart tissue samples and cytokine-treated human cardiomyocytes *in vitro*. Although some of the changes observed in IFN-γ and TNF-α-treated AC-16 cardiomyocytes closely parallel those observed in CCC heart tissue, this convergence is not proof that the findings in tissue are induced by the same cytokines, since several other mechanisms can induce nitro-oxidative stress and mitochondrial damage in CCC myocardium and as a consequence of heart failure. Conversely, our results with cytokine-treated cardiomyocytes can bring insight not only about Chagas disease, but also in other cardiac disorders where IFN-γ and TNF-α play a role, such as inflammatory cardiomyopathies of other etiology.

**Figure 8:**
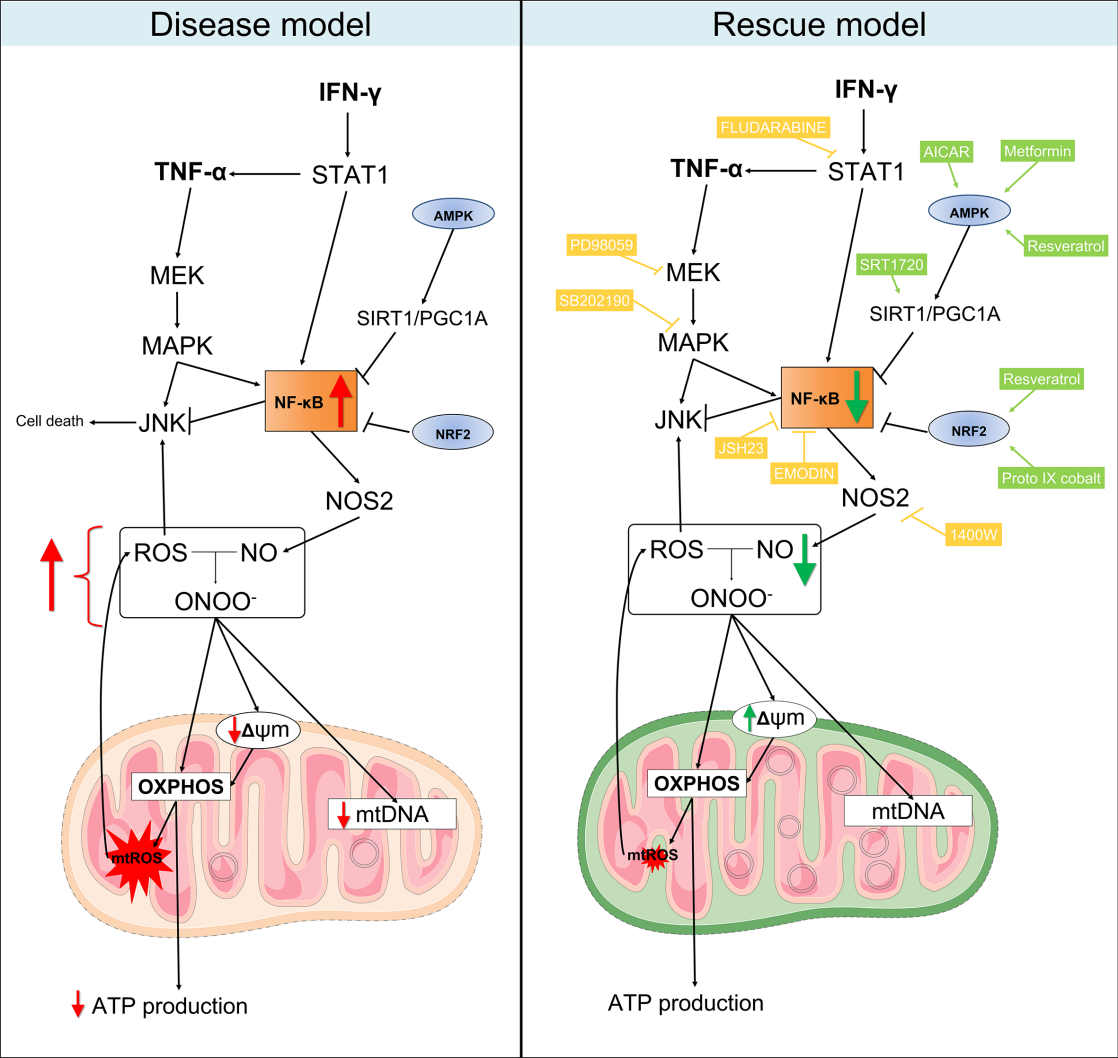
Proposed model for the mitochondrial dysfunction in Chagas disease cardiomyopathy. Left panel: in summary, our results showed that heart tissues from CCC patients have increased production of RNS and reduced content of mitochondrial DNA in comparison with patients with DCM. Similarly, stimulation of AC-16 cardiomyocytes with IFN-γ/TNF-α increased ROS, RNS and proton leak, impaired ΔΨm, depleted ATP production and changed the metabolic profile of the cells. Right panel: in our rescue model, we showed that pharmacological inhibition of molecules involved in the IFN-γ/TNF-α/NF-κB/NOS2 pathway ameliorated the ΔΨm and NO production. Additionally, activation of AMPK/SIRT1 and NRF2 had beneficial impact on the ΔΨm. Thus, we hypothesize that mitochondrial dysfunction is driven by the excessive production of IFN-γ/TNF-α in CCC myocardium and is an essential component for the poor prognosis of Chagas disease cardiomyopathy. Mitochondria-targeted therapies might improve CCC disease progression. Compounds in yellow are inhibitors; Compounds in light green are agonists. Connecting arrows indicate activation and flat line means inhibitory interaction. Red and green arrows indicate the changes observed before and after treatment with the compounds.

This study demonstrated that stimulation with IFN-γ and TNF-α in human cardiomyocytes causes mitochondrial damage, oxidative and nitrosative stress, paralleling events observed in the cytokine-rich CCC heart tissue. It is important to notice that the part of the CCC samples analyzed here have been previously assessed for cytokine expression and IFN-γ was among the most highly expressed cytokine, while IFN-γ and TNF-α were the top upstream regulators (9, 36). Both CCC myocardium and stimulated cells exhibited damaging profiles in markers of cellular stress and increased ROS and RNS, and decreased ATP. Several mitochondrial-related pathways important for mitochondrial integrity, function and ATP production were dysregulated in cytokine-stimulated cells. Also, cytokine-stimulated cells exhibited impaired ΔΨm and increased ROS and RNS and higher amounts of ROS. It is important to point out the direct involvement of the STAT1/NF-κB/NOS2 signaling pathway in the damaging effects of IFN-γ and TNF-α in cardiomyocytes’ ΔΨm, as well as the restorative effects of stimulating the AMPK, NRF2 and SIRT1 pathways (**Figure 8**). Our results suggest that cytokine-induced disturbances in mitochondrial function and energy metabolism might play a role in the worse prognosis of Chagas disease cardiomyopathy. Therapy targeting mitochondrial function and energy imbalance should thus in principle be beneficial to restore cardiac function in CCC and other IFN-γ-dependent inflammatory heart diseases, like viral myocarditis and inflammatory cardiomyopathy of other etiologies, age-related myocardial inflammation and functional decline (80), myocardial infarction (81), and anthracycline antitumoral agent cardiotoxicity (65).

Further studies with induced pluripotent stem cells derived cardiomyocytes (iPS-CM) can be employed to investigate, in a personalized, patient-specific manner, the effect of the cytokines in mitochondrial function.

## Data Availability Statement

The original contributions presented in the study are included in the article/**Supplementary Material**. Further inquiries can be directed to the corresponding authors.

## Ethics Statement

The protocol was also approved by the INSERM Internal Review Board and the Brazilian National Ethics in Research Commission (CONEP). The patients/participants provided their written informed consent to participate in this study.

## Author Contributions

Study design: EC-N, CC, JN, FL, and JK. Phenotype characterization: EB, RS, FB, and PP. Experimental analysis: JN, PA, RA, EK, AH, LI, DA-S, KS, RV, DL, SB, FG, MT, and PT. Statistical analysis: HN, JN, PB, DG, and MM. Manuscript preparation: EC-N, CC, JN, and VP. All authors contributed to the article and approved the submitted version.

## Funding

This work was supported by the Institut National de la Santé et de la Recherche Médicale (INSERM); the Aix-Marseille University (grant number: AMIDEX “International_2018” MITOMUTCHAGAS); the French Agency for Research (Agence Nationale de la Recherche-ANR (grant numbers: “Br-Fr-Chagas”, “landscardio”); the CNPq (Brazilian Council for Scientific and Technological Development); and the FAPESP (São Paulo State Research Funding Agency Brazil (grant numbers: 2013/50302-3, 2014/50890-5); the National Institutes of Health/USA (grant numbers: 2P50AI098461-02 and 2U19AI098461-06). This work was founded by the Inserm Cross-Cutting Project GOLD. This project has received funding from the Excellence Initiative of Aix-Marseille University - A*Midex a French “Investissements d’Avenir programme”- Institute MarMaRa AMX-19-IET-007. JN was a recipient of a MarMaRa fellowship. ECN and JK are recipients of productivity awards by CNPq. The funders did not play any role in the study design, data collection and analysis, decision to publish, or preparation of the manuscript.

## Conflict of Interest

The authors declare that the research was conducted in the absence of any commercial or financial relationships that could be construed as a potential conflict of interest.

## Publisher’s Note

All claims expressed in this article are solely those of the authors and do not necessarily represent those of their affiliated organizations, or those of the publisher, the editors and the reviewers. Any product that may be evaluated in this article, or claim that may be made by its manufacturer, is not guaranteed or endorsed by the publisher.

## Supplementary Material

Supplementary Figure 1 Stratification of mitochondria area and membrane potential after stimulation with IFN-γ and TNF-α in human cardiomyocytes AC-16 cells. Cells were stimulated with 10 ng/ml of IFN-γ + 5 ng/ml of TNF-α for 48 hours and then multi-labelled with TMRM, Mitotracker DeepRed, NucGreen and DAPI. TMRM fluorescence was measured when colocalized with the fluorescence of Mitotracker Deep Red in live cells (NucGreen-negative). Mitochondria were stratified based on their area in: lower than 5 **(A)**, between 5-10 **(B)**, between 10-25 **(C)**, between 25-50 **(D)** and **(E)** higher than 50 µm^2^. **(F)** average (± SEM) value obtained from the mitochondrial membrane potential of each stratification of mitochondrial size. ****p < 0.0001; Mann Whitney test.

Supplementary Figure 2 Compound screening in AC-16 mitochondrial membrane potential. AC-16 cells were treated with serially diluted agonists of AMPK **(A)**, NRF2 **(B)**, SIRT1 **(C)** or inhibitors of NF-κB **(D)**, IKKβ **(E)**, STAT1 **(F)**, NOS2 **(G)**, MEK1 and MEK2 **(H)**, JNK **(I)** and MAPK **(J)** alone (blue line) or in combination with 10 ng/ml IFN-γ (red line) or 10 ng/ml of IFN-γ plus 5 ng/ml TNF-α (green line) for 48 hours. Specific doses were selected based on the highest effect on mitochondrial ΔΨm and less than 10% loss on cell number. The first dot of the titration (to the left) is without compounds. Grey arrows indicate concentrations selected for . All data are shown as percentage to not-stimulated cells. Standard error of the mean is from 3 independent experiments.

Supplementary Figure 3 Compound screening in the cell number of AC-16. AC-16 cells were treated with serially diluted agonists of AMPK **(A)**, NRF2 **(B)**, SIRT1 **(C)** or inhibitors of NF-κB **(D)**, IKKβ **(E)**, STAT1 **(F)**, NOS2 **(G)**, MEK1 and MEK2 **(H)**, JNK **(I)**, MAPK **(J)** and DMSO **(K)** alone (blue line) or in combination with 10 ng/ml of IFN-γ (red line) or 10 ng/ml of IFN-γ plus 5 ng/ml of TNF-α (green line) for 48 hours. The first dot of each titration (to the left) is without compounds. Grey arrows indicate concentrations selected for. Standard error of the mean is from 3 independent experiments.

Supplementary Figure 4 Schematic representation of the inhibitors and agonists and their targets in the IFN-γ and TNF-α signaling pathways. The pro-inflammatory cytokine IFN-γ interacts with its transmembrane receptor IFNGR1 and signals mainly through the signal transducer and activator of transcription 1 (STAT1) and Interferon Regulatory Factor 1 (IRF1) intracellular transduction pathway to achieve transcriptional activation of IFN-γ-inducible genes, such as TNF-α and Nuclear Factor Kappa B (NF-κB). Activation of NF-κB leads to an upregulation of Nitric oxide synthase 2 (NOS2) gene, which in turn increases the amount of intracellular nitric oxide (NO), reacting to reactive oxygen species (ROS) to generate peroxynitrite (ONOO^-^). Arrows indicate activation and flat line means inhibitory interaction. While ONOO^-^ is important for pathogenic response, it causes damages to mitochondria, leading to reduction of mitochondria membrane potential and fragmentation. Proteins such AMPK and NRF2 are involved in the cellular response to oxidative stress countering the damaging effects of NF-κB. Compounds highlighted in yellow are inhibitors and green are agonists.

Supplementary Figure 5 Transcriptomic analysis on AC-16 cardiomyocyte cell line stimulated with IFN-γ and TNF-α. AC-16 cells were stimulated with IFN-γ and TNF-α during 1h to 48h. Gene expression analysis was done between the various time points taking as reference the T=0h time point. At each time is provided the volcano plot and the heatmap. Each stimulation was performed 4 times.
