# Supplementary figures and images for: Co-Exposure of Cardiomyocytes to IFN-γ and TNF-α Induces Mitochondrial Dysfunction and Nitro-Oxidative Stress: Implications for the Pathogenesis of Chronic Chagas Disease Cardiomyopathy
Source: Front Immunol. 2021 Nov 11;12:755862. doi: 10.3389/fimmu.2021.755862 (PMC8632642; doi:10.3389/fimmu.2021.755862)

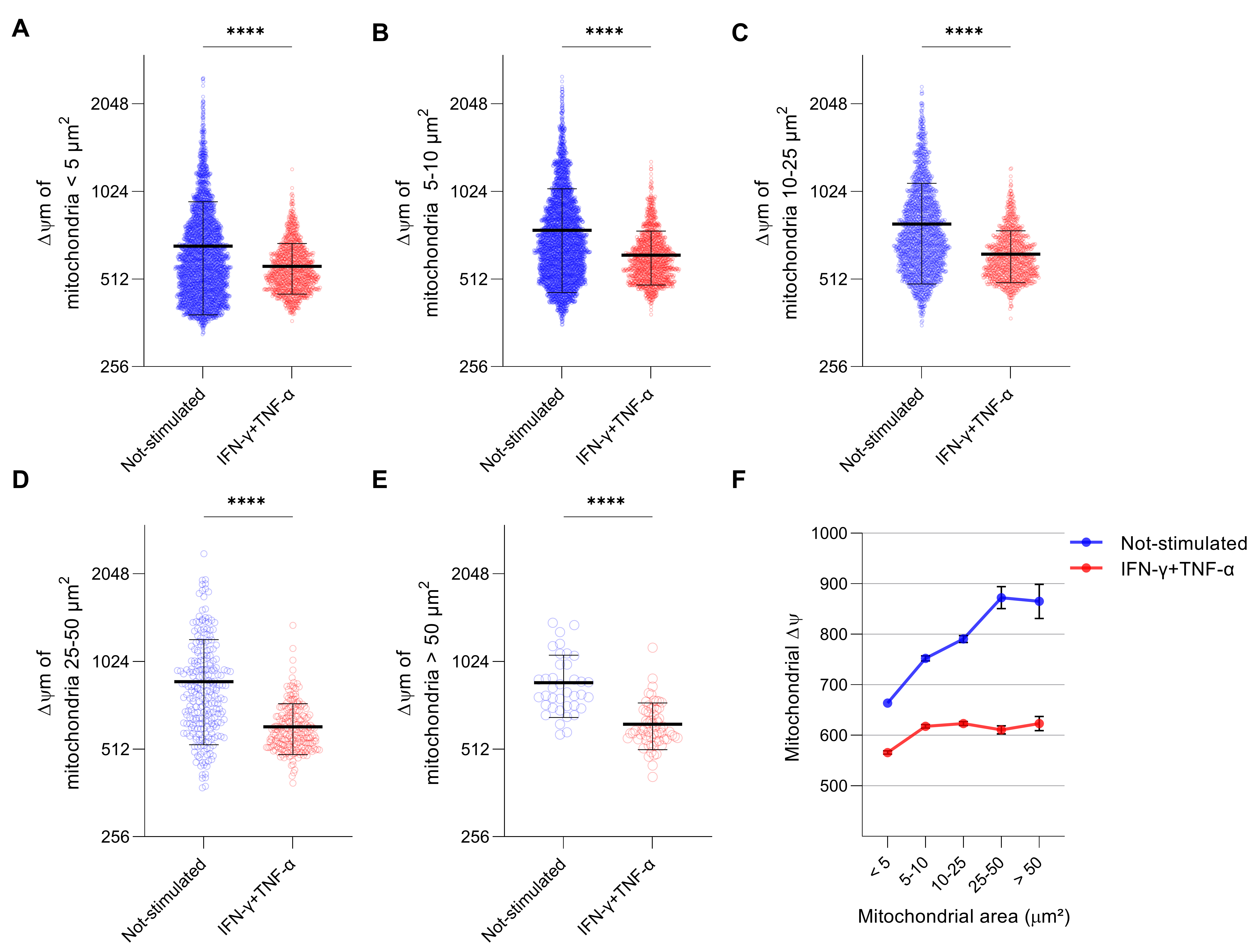

Supplement: Supplementary Figure 1 — Stratification of mitochondria area and membrane potential after stimulation with IFN-γ and TNF-α in human cardiomyocytes AC-16 cells. Cells were stimulated with 10 ng/ml of IFN-γ + 5 ng/ml of TNF-α for 48 hours and then multi-labelled with TMRM, Mitotracker DeepRed, NucGreen and DAPI. TMRM fluorescence was measured when colocalized with the fluorescence of Mitotracker Deep Red in live cells (NucGreen-negative). Mitochondria were stratified based on their area in: lower than 5 (A), between 5-10 (B), between 10-25 (C), between 25-50 (D) and (E) higher than 50 µm2. (F) average (± SEM) value obtained from the mitochondrial membrane potential of each stratification of mitochondrial size. ****p < 0.0001; Mann Whitney test. [file Image_1.tif]

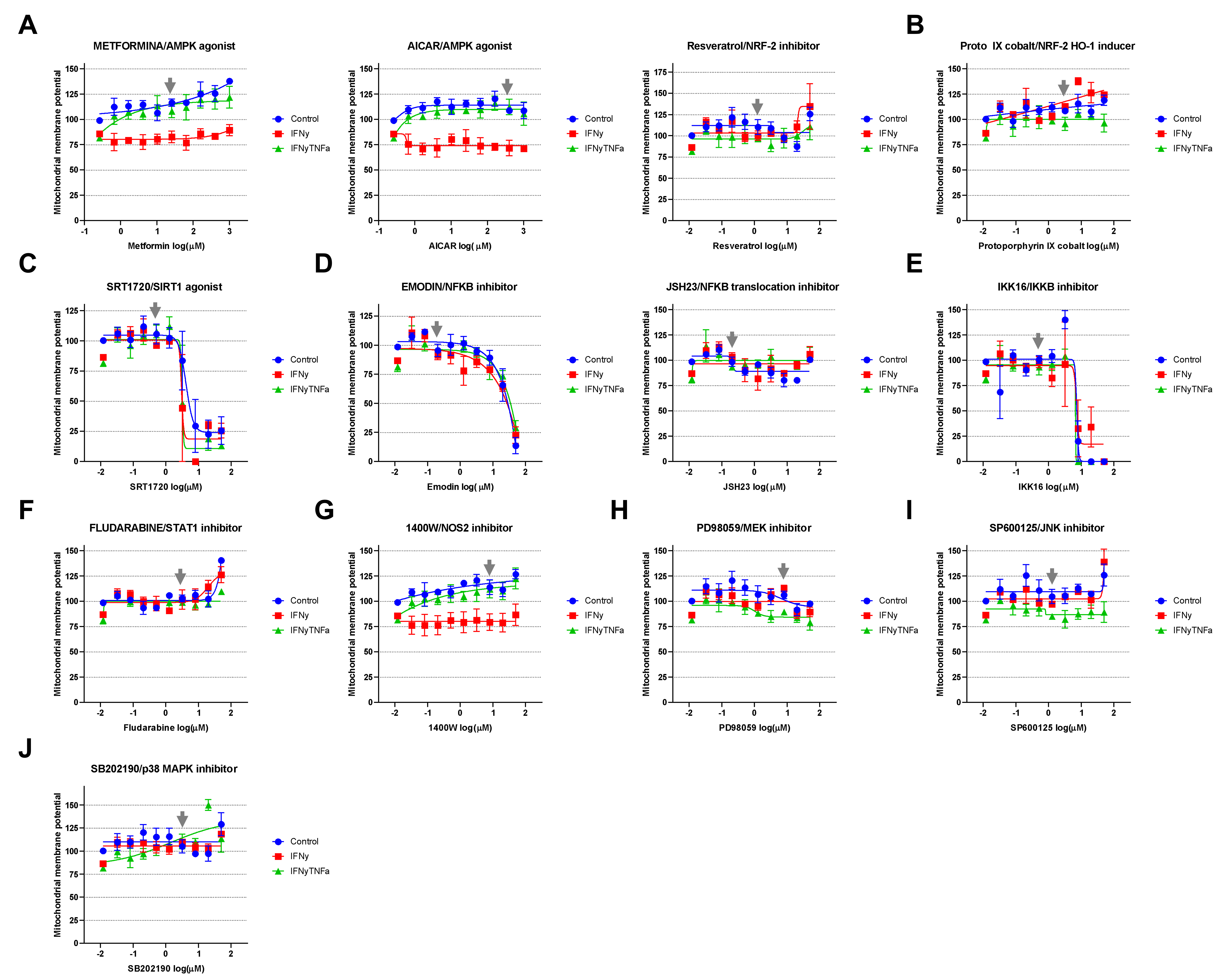

Supplement: Supplementary Figure 2 — Compound screening in AC-16 mitochondrial membrane potential. AC-16 cells were treated with serially diluted agonists of AMPK (A), NRF2 (B), SIRT1 (C) or inhibitors of NF-κB (D), IKKβ (E), STAT1 (F), NOS2 (G), MEK1 and MEK2 (H), JNK (I) and MAPK (J) alone (blue line) or in combination with 10 ng/ml IFN-γ (red line) or 10 ng/ml of IFN-γ plus 5 ng/ml TNF-α (green line) for 48 hours. Specific doses were selected based on the highest effect on mitochondrial ΔΨm and less than 10% loss on cell number. The first dot of the titration (to the left) is without compounds. Grey arrows indicate concentrations selected for . All data are shown as percentage to not-stimulated cells. Standard error of the mean is from 3 independent experiments. [file Image_2.tif]

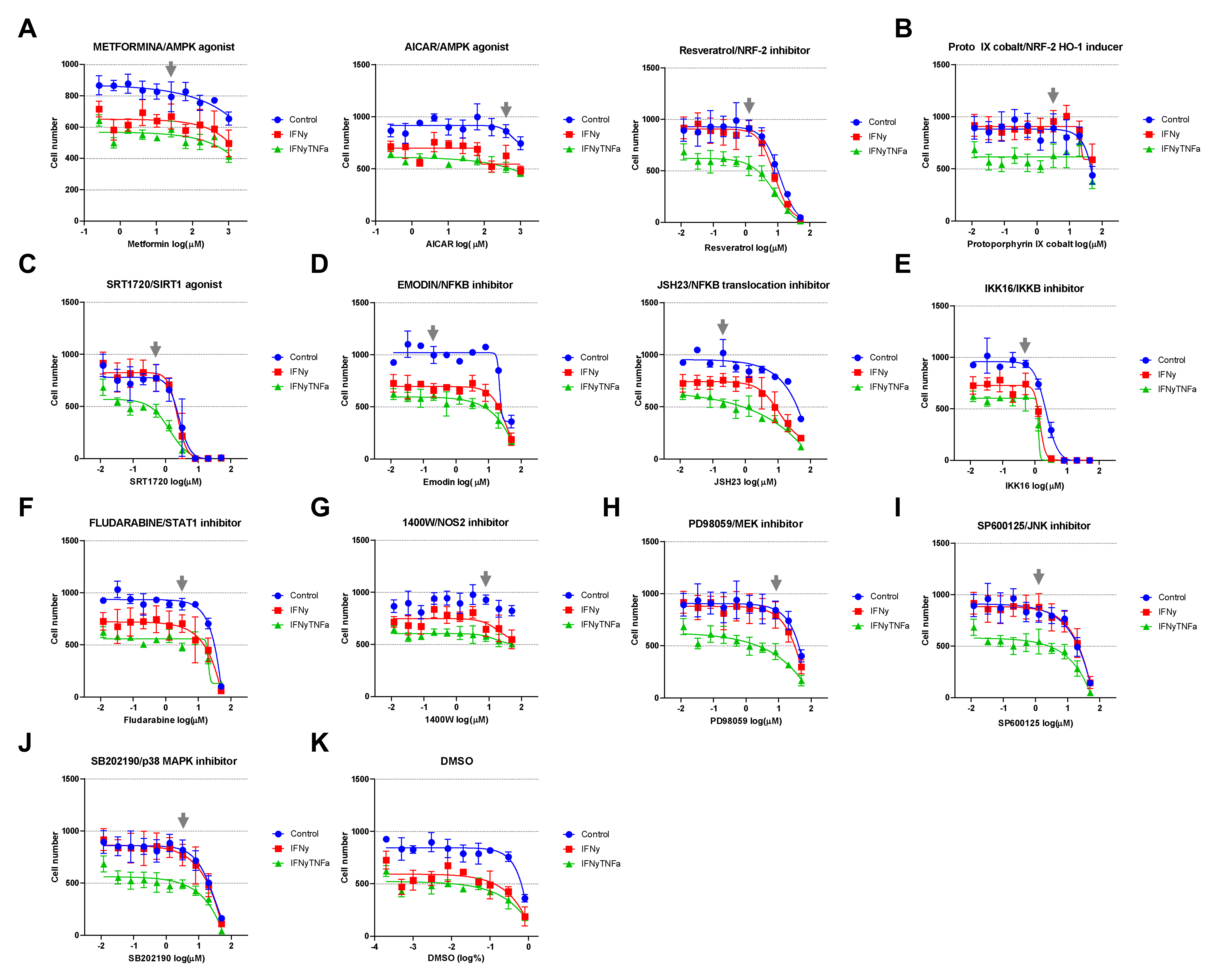

Supplement: Supplementary Figure 3 — Compound screening in the cell number of AC-16. AC-16 cells were treated with serially diluted agonists of AMPK (A), NRF2 (B), SIRT1 (C) or inhibitors of NF-κB (D), IKKβ (E), STAT1 (F), NOS2 (G), MEK1 and MEK2 (H), JNK (I), MAPK (J) and DMSO (K) alone (blue line) or in combination with 10 ng/ml of IFN-γ (red line) or 10 ng/ml of IFN-γ plus 5 ng/ml of TNF-α (green line) for 48 hours. The first dot of each titration (to the left) is without compounds. Grey arrows indicate concentrations selected for. Standard error of the mean is from 3 independent experiments. [file Image_3.tif]

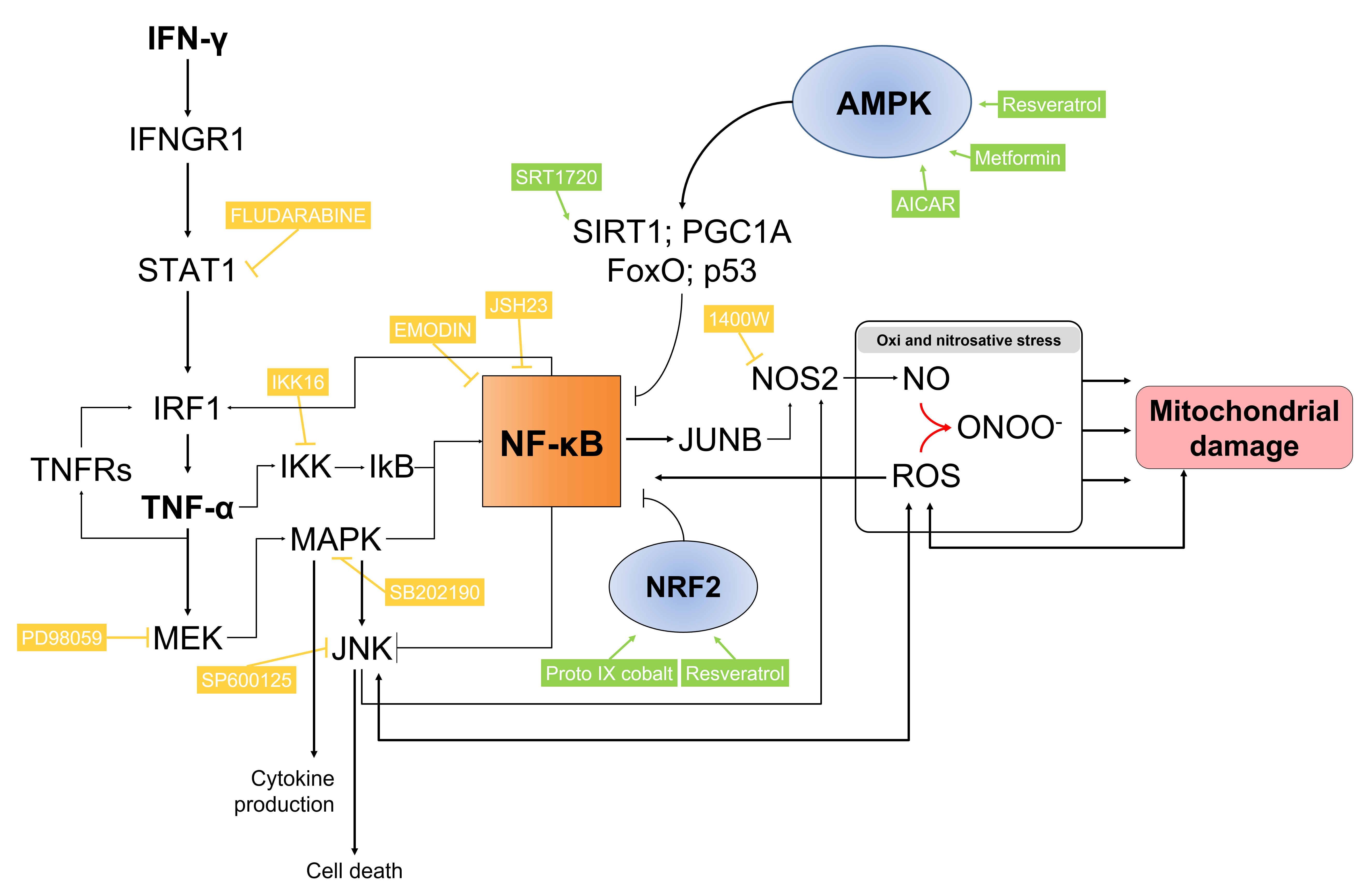

Supplement: Supplementary Figure 4 — Schematic representation of the inhibitors and agonists and their targets in the IFN-γ and TNF-α signaling pathways. The pro-inflammatory cytokine IFN-γ interacts with its transmembrane receptor IFNGR1 and signals mainly through the signal transducer and activator of transcription 1 (STAT1) and Interferon Regulatory Factor 1 (IRF1) intracellular transduction pathway to achieve transcriptional activation of IFN-γ-inducible genes, such as TNF-α and Nuclear Factor Kappa B (NF-κB). Activation of NF-κB leads to an upregulation of Nitric oxide synthase 2 (NOS2) gene, which in turn increases the amount of intracellular nitric oxide (NO), reacting to reactive oxygen species (ROS) to generate peroxynitrite (ONOO-). Arrows indicate activation and flat line means inhibitory interaction. While ONOO- is important for pathogenic response, it causes damages to mitochondria, leading to reduction of mitochondria membrane potential and fragmentation. Proteins such AMPK and NRF2 are involved in the cellular response to oxidative stress countering the damaging effects of NF-κB. Compounds highlighted in yellow are inhibitors and green are agonists. [file Image_4.tif]

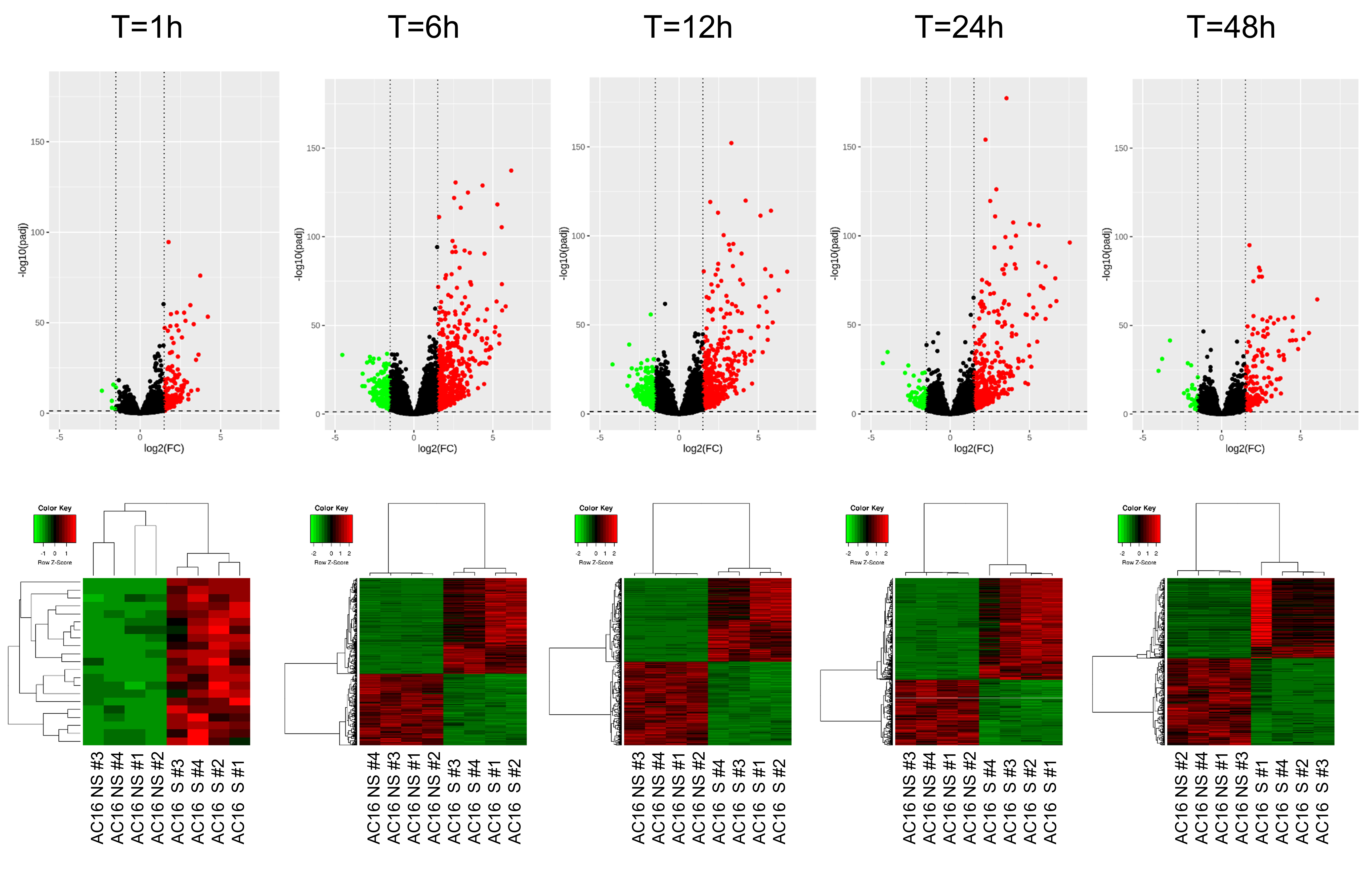

Supplement: Supplementary Figure 5 — Transcriptomic analysis on AC-16 cardiomyocyte cell line stimulated with IFN-γ and TNF-α. AC-16 cells were stimulated with IFN-γ and TNF-α during 1h to 48h. Gene expression analysis was done between the various time points taking as reference the T=0h time point. At each time is provided the volcano plot and the heatmap. Each stimulation was performed 4 times. [file Image_5.tif]
