# Supplementary material for: Co-Exposure of Cardiomyocytes to IFN-γ and TNF-α Induces Mitochondrial Dysfunction and Nitro-Oxidative Stress: Implications for the Pathogenesis of Chronic Chagas Disease Cardiomyopathy
Source: Front Immunol. 2021 Nov 11;12:755862. doi: 10.3389/fimmu.2021.755862 (PMC8632642; doi:10.3389/fimmu.2021.755862)
Supplement: Supplementary file 6 [file Table_1.docx]

**Supplementary Table 1:** List of differentially expressed proteins in AC16 stimulated with IFN-γ and TNF-α

**Mitochondrial proteins**

| **Protein ID** | **Protein name** | **Gene** | **FC** | **p-value** |
| --- | --- | --- | --- | --- |
| P21399 | Cytoplasmic aconitate hydratase | ACO1 | 15,61 | 1,1E-02 |
| P30405-2 | Peptidyl-prolyl cis-trans isomerase F, mitochondrial | PPIF | 9,64 | 2,7E-02 |
| P04179-3 | Superoxide dismutase [Mn], mitochondrial | SOD2 | 9,10 | 3,2E-02 |
| Q9Y305-3 | Acyl-coenzyme A thioesterase 9, mitochondrial | ACOT9 | 8,55 | 3,0E-03 |
| P28838-2 | Cytosol aminopeptidase | LAP3 | 7,95 | 1,1E-03 |
| Q9NQR4 | Omega-amidase NIT2 | NIT2 | 4,54 | 2,5E-02 |
| Q92552 | 28S ribosomal protein S27, mitochondrial | MRPS27 | 2,34 | 4,7E-02 |
| P00387-2 | NADH-cytochrome b5 reductase 3 | CYB5R3 | 1,65 | 4,6E-02 |
| P12236 | ADP/ATP translocase 3 | SLC25A6 | 1,39 | 2,7E-02 |
| Q9UII2 | ATPase inhibitor, mitochondrial | ATPIF1 | 1,29 | 8,3E-03 |
| Q96IX5 | Up-regulated during skeletal muscle growth protein 5 | USMG5 | 1,21 | 2,5E-02 |
| P10809 | 60 kDa heat shock protein, mitochondrial | HSPD1 | 1,16 | 1,2E-02 |
| P30044-2 | Peroxiredoxin-5, mitochondrial | PRDX5 | 0,93 | 2,4E-02 |
| O00217 | NADH dehydrogenase [ubiquinone] iron-sulfur protein 8, mitochondrial | NDUFS8 | 0,84 | 5,0E-02 |
| P30048-2 | Thioredoxin-dependent peroxide reductase, mitochondrial | PRDX3 | 0,78 | 8,9E-03 |
| P49327 | Fatty acid synthase | FASN | 0,77 | 1,4E-02 |
| Q16836 | Hydroxyacyl-coenzyme A dehydrogenase, mitochondrial | HADH | 0,76 | 2,4E-02 |
| P54136 | Arginine--tRNA ligase, cytoplasmic | RARS | 0,74 | 5,0E-03 |
| Q9NPJ3-2 | Acyl-coenzyme A thioesterase 13 | ACOT13 | 0,72 | 4,9E-04 |
| Q02978-2 | Mitochondrial 2-oxoglutarate/malate carrier protein | SLC25A11 | 0,71 | 1,4E-02 |
| Q16891-2 | MICOS complex subunit MIC60 | IMMT | 0,71 | 1,4E-02 |
| O75390 | Citrate synthase, mitochondrial | CS | 0,71 | 1,4E-02 |
| P24752 | Acetyl-CoA acetyltransferase, mitochondrial | ACAT1 | 0,68 | 2,3E-05 |
| P19404 | NADH dehydrogenase [ubiquinone] flavoprotein 2, mitochondrial | NDUFV2 | 0,66 | 2,4E-02 |
| Q12931-2 | Heat shock protein 75 kDa, mitochondrial | TRAP1 | 0,66 | 9,5E-03 |
| P07954-2 | Fumarate hydratase, mitochondrial | FH | 0,61 | 7,5E-03 |
| O95831-3 | Apoptosis-inducing factor 1, mitochondrial | AIFM1 | 0,60 | 6,3E-03 |
| Q00325-2 | Phosphate carrier protein, mitochondrial | SLC25A3 | 0,59 | 9,7E-03 |
| Q8TBP6 | Solute carrier family 25 member 40 | SLC25A40 | 0,58 | 1,6E-02 |
| Q9Y696 | Chloride intracellular channel protein 4 | CLIC4 | 0,55 | 4,6E-02 |
| Q9Y5L4 | Mitochondrial import inner membrane translocase subunit Tim13 | TIMM13 | 0,48 | 2,6E-03 |
| P10515 | Dihydrolipoyllysine-residue acetyltransferase component of pyruvate dehydrogenase complex, mitochondrial | DLAT | 0,45 | 3,5E-02 |
| Q07812-5 | Apoptosis regulator BAX | BAX | 0,38 | 4,7E-02 |
| Q9NS69 | Mitochondrial import receptor subunit TOM22 homolog | TOMM22 | 0,37 | 3,6E-02 |
| P55809 | Succinyl-CoA:3-ketoacid coenzyme A transferase 1, mitochondrial | OXCT1 | 0,24 | 2,2E-02 |
| Q99798 | Aconitate hydratase, mitochondrial | ACO2 | 0,11 | 6,0E-03 |
| Q12849-5 | G-rich sequence factor 1 | GRSF1 | 0,10 | 6,0E-03 |

**Non-mitochondrial proteins**

| **Protein ID** | **Protein name** | **Gene name** | **FC** | **p-value** |
| --- | --- | --- | --- | --- |
| P32455 | Interferon-induced guanylate-binding protein 1 | GBP1 | 274,05 | 4,2E-03 |
| P23381 | Tryptophan--tRNA ligase, cytoplasmic | WARS | 237,56 | 2,4E-02 |
| P05362 | Intercellular adhesion molecule 1 | ICAM1 | 105,18 | 8,9E-03 |
| Q03518 | Antigen peptide transporter 1 | TAP1 | 98,43 | 2,0E-03 |
| O14879 | Interferon-induced protein with tetratricopeptide repeats 3 | IFIT3 | 75,70 | 2,0E-03 |
| P10321-2 | HLA class I histocompatibility antigen, Cw-7 alpha chain | HLA-C | 56,01 | 1,2E-02 |
| P20591-2 | Interferon-induced GTP-binding protein Mx1 | MX1 | 41,02 | 1,2E-04 |
| P42224 | Signal transducer and activator of transcription 1-alpha/beta | STAT1 | 37,19 | 1,2E-04 |
| P09913 | Interferon-induced protein with tetratricopeptide repeats 2 | IFIT2 | 25,46 | 1,6E-02 |
| Q9UL46 | Proteasome activator complex subunit 2 | PSME2 | 24,32 | 3,0E-03 |
| Q03519 | Antigen peptide transporter 2 | TAP2 | 24,18 | 2,7E-02 |
| Q9NZ08 | Endoplasmic reticulum aminopeptidase 1 | ERAP1 | 16,91 | 1,5E-04 |
| P04439 | HLA class I histocompatibility antigen, A-3 alpha chain | HLA-A | 16,90 | 7,2E-03 |
| Q9BQE5 | Apolipoprotein L2 | APOL2 | 16,70 | 1,4E-03 |
| O43396 | Thioredoxin-like protein 1 | TXNL1 | 16,69 | 1,5E-04 |
| O14933-2 | Ubiquitin/ISG15-conjugating enzyme E2 L6 | UBE2L6 | 15,77 | 4,8E-03 |
| P01889 | HLA class I histocompatibility antigen, B-7 alpha chain | HLA-B | 14,44 | 1,9E-03 |
| Q9Y6K5 | 2-5-oligoadenylate synthase 3 | OAS3 | 13,82 | 2,1E-02 |
| P29590-14 | Protein PML | PML | 13,36 | 6,0E-03 |
| P14902 | Indoleamine 2,3-dioxygenase 1 | IDO1 | 12,52 | 1,8E-02 |
| Q7Z2W4-2 | Zinc finger CCCH-type antiviral protein 1 | ZC3HAV1 | 12,10 | 2,5E-04 |
| Q96CV9-2 | Optineurin | OPTN | 10,48 | 2,5E-02 |
| Q9Y3Z3-4 | Deoxynucleoside triphosphate triphosphohydrolase SAMHD1 | SAMHD1 | 10,36 | 6,4E-03 |
| Q16666-3 | Gamma-interferon-inducible protein 16 | IFI16 | 10,10 | 9,7E-03 |
| P61769 | Beta-2-microglobulin | B2M | 9,47 | 1,2E-03 |
| Q9BQ51-3 | Programmed cell death 1 ligand 2 | PDCD1LG2 | 9,34 | 1,8E-02 |
| P49662-4 | Caspase-4 | CASP4 | 9,17 | 1,8E-02 |
| Q9ULX9-2 | Transcription factor MafF | MAFF | 7,05 | 8,9E-03 |
| Q08170 | Serine/arginine-rich splicing factor 4 | SRSF4 | 6,50 | 1,2E-04 |
| P28062 | Proteasome subunit beta type-8 | PSMB8 | 6,46 | 4,9E-03 |
| Q08380 | Galectin-3-binding protein | LGALS3BP | 5,93 | 1,9E-02 |
| Q06323-3 | Proteasome activator complex subunit 1 | PSME1 | 4,87 | 4,9E-03 |
| Q96PP8 | Guanylate-binding protein 5 | GBP5 | 4,55 | 2,1E-02 |
| P21589-2 | 5-nucleotidase | NT5E | 4,30 | 1,7E-02 |
| P60028 | Rab GDP dissociation inhibitor alpha | GDI1 | 3,97 | 2,4E-02 |
| Q86UP2-2 | Kinectin | KTN1 | 3,81 | 1,7E-02 |
| P28065-2 | Proteasome subunit beta type-9 | PSMB9 | 3,80 | 1,3E-02 |
| P17096 | High mobility group protein HMG-I/HMG-Y | HMGA1 | 3,72 | 4,5E-02 |
| P30740-2 | Leukocyte elastase inhibitor | SERPINB1 | 3,58 | 1,5E-03 |
| P23497-7 | Nuclear autoantigen Sp-100 | SP100 | 2,76 | 9,7E-04 |
| Q96KP4 | Cytosolic non-specific dipeptidase | CNDP2 | 2,73 | 2,8E-02 |
| P05976-2 | Myosin light chain 1/3, skeletal muscle isoform | MYL1 | 2,58 | 4,2E-03 |
| Q8WVM8-2 | Sec1 family domain-containing protein 1 | SCFD1 | 2,46 | 4,0E-02 |
| Q14764 | Major vault protein | MVP | 2,36 | 4,1E-03 |
| Q9NXG0 | Centlein | CNTLN | 2,33 | 2,7E-02 |
| Q5VUR7 | Ankyrin repeat domain-containing protein 20A3 | ANKRD20A3 | 2,27 | 1,1E-03 |
| Q96FQ6 | Protein S100-A16 | S100A16 | 2,25 | 2,1E-03 |
| Q9HD42 | Charged multivesicular body protein 1a | CHMP1A | 2,16 | 2,4E-02 |
| P27635 | 60S ribosomal protein L10 | RPL10 | 2,08 | 4,7E-02 |
| Q9UNL2 | Translocon-associated protein subunit gamma | SSR3 | 2,03 | 3,7E-02 |
| P25787 | Proteasome subunit alpha type-2 | PSMA2 | 2,00 | 4,7E-02 |
| Q9UMR2-2 | ATP-dependent RNA helicase DDX19B | DDX19B | 1,96 | 1,5E-02 |
| Q9UI30-2 | Multifunctional methyltransferase subunit TRM112-like protein | TRMT112 | 1,95 | 4,9E-03 |
| P49720 | Proteasome subunit beta type-3 | PSMB3 | 1,78 | 4,7E-02 |
| P0DMV9 | Heat shock 70 kDa protein 1B | HSPA1B | 1,77 | 1,6E-02 |
| P16070-18 | CD44 antigen | CD44 | 1,75 | 2,3E-02 |
| P07437 | Tubulin beta chain | TUBB | 1,75 | 1,4E-02 |
| P61165 | Transmembrane protein 258 | TMEM258 | 1,73 | 4,5E-02 |
| P08670 | Vimentin | VIM | 1,73 | 3,2E-03 |
| P18669 | Phosphoglycerate mutase 1 | PGAM1 | 1,71 | 1,2E-02 |
| P55265-5 | Double-stranded RNA-specific adenosine deaminase | ADAR | 1,71 | 2,4E-02 |
| P15153 | Ras-related C3 botulinum toxin substrate 2 | RAC2 | 1,70 | 3,0E-03 |
| P62937 | Peptidyl-prolyl cis-trans isomerase A | PPIA | 1,70 | 1,6E-02 |
| Q05519-2 | Serine/arginine-rich splicing factor 11 | SRSF11 | 1,69 | 8,5E-03 |
| Q9BUF5 | Tubulin beta-6 chain | TUBB6 | 1,68 | 6,9E-03 |
| Q71U36-2 | Tubulin alpha-1A chain | TUBA1A | 1,67 | 2,7E-02 |
| Q9H4M9 | EH domain-containing protein 1 | EHD1 | 1,66 | 2,5E-02 |
| Q14694 | Ubiquitin carboxyl-terminal hydrolase 10 | USP10 | 1,63 | 2,6E-02 |
| Q9UKM9-2 | RNA-binding protein Raly | RALY | 1,60 | 1,5E-03 |
| P08133-2 | Annexin A6 | ANXA6 | 1,60 | 1,6E-02 |
| O00151 | PDZ and LIM domain protein 1 | PDLIM1 | 1,53 | 2,0E-03 |
| Q14697 | Neutral alpha-glucosidase AB | GANAB | 1,52 | 1,2E-02 |
| Q15599-3 | Na(+)/H(+) exchange regulatory cofactor NHE-RF2 | SLC9A3R2 | 1,52 | 1,8E-02 |
| P54920 | Alpha-soluble NSF attachment protein | NAPA | 1,50 | 3,7E-02 |
| P14618 | Pyruvate kinase PKM | PKM | 1,50 | 4,5E-03 |
| Q14157-4 | Ubiquitin-associated protein 2-like | UBAP2L | 1,46 | 1,4E-02 |
| Q9Y266 | Nuclear migration protein nudC | NUDC | 1,44 | 3,8E-03 |
| O75396 | Vesicle-trafficking protein SEC22b | SEC22B | 1,44 | 2,3E-05 |
| P62942 | Peptidyl-prolyl cis-trans isomerase FKBP1A | FKBP1A | 1,43 | 1,9E-02 |
| Q9H3K6-2 | BolA-like protein 2 | BOLA2 | 1,43 | 2,9E-02 |
| P17655 | Calpain-2 catalytic subunit | CAPN2 | 1,41 | 3,7E-02 |
| O00231 | 26S proteasome non-ATPase regulatory subunit 11 | PSMD11 | 1,40 | 2,0E-02 |
| Q9Y3I0 | tRNA-splicing ligase RtcB homolog | RTCB | 1,40 | 2,4E-04 |
| Q9Y5K6 | CD2-associated protein | CD2AP | 1,40 | 3,2E-02 |
| P62306 | Small nuclear ribonucleoprotein F | SNRPF | 1,39 | 3,0E-02 |
| Q8IYB3-2 | Serine/arginine repetitive matrix protein 1 | SRRM1 | 1,39 | 9,7E-03 |
| P11021 | 78 kDa glucose-regulated protein | HSPA5 | 1,39 | 2,5E-02 |
| Q00535-2 | Cyclin-dependent-like kinase 5 | CDK5 | 1,39 | 1,2E-02 |
| O60884 | DnaJ homolog subfamily A member 2 | DNAJA2 | 1,37 | 1,3E-02 |
| P53814-5 | Smoothelin | SMTN | 1,37 | 5,0E-02 |
| P26006 | Integrin alpha-3 | ITGA3 | 1,37 | 1,9E-02 |
| P62491-2 | Ras-related protein Rab-11A | RAB11A | 1,35 | 3,4E-02 |
| P62195-2 | 26S protease regulatory subunit 8 | PSMC5 | 1,33 | 4,0E-02 |
| P30101 | Protein disulfide-isomerase A3 | PDIA3 | 1,32 | 4,5E-02 |
| Q5SW79-2 | Centrosomal protein of 170 kDa | CEP170 | 1,31 | 4,7E-02 |
| Q14195-2 | Dihydropyrimidinase-related protein 3 | DPYSL3 | 1,31 | 7,5E-03 |
| P17096-2 | High mobility group protein HMG-I/HMG-Y | HMGA1 | 1,31 | 4,8E-02 |
| P00558 | Phosphoglycerate kinase 1 | PGK1 | 1,29 | 4,6E-02 |
| O95782-2 | AP-2 complex subunit alpha-1 | AP2A1 | 1,28 | 4,2E-03 |
| Q16543 | Hsp90 co-chaperone Cdc37 | CDC37 | 1,26 | 1,9E-02 |
| P38919 | Eukaryotic initiation factor 4A-III | EIF4A3 | 1,25 | 6,0E-03 |
| P68371 | Tubulin beta-4B chain | TUBB4B | 1,25 | 3,4E-04 |
| Q16778 | Histone H2B type 2-E | HIST2H2BE | 1,25 | 4,7E-02 |
| O14979-3 | Heterogeneous nuclear ribonucleoprotein D-like | HNRNPDL | 1,20 | 2,6E-02 |
| P14625 | Endoplasmin | HSP90B1 | 1,19 | 1,5E-02 |
| P62280 | 40S ribosomal protein S11 | RPS11 | 1,19 | 5,2E-04 |
| Q5VTE0 | Putative elongation factor 1-alpha-like 3 | EEF1A1P5 | 1,19 | 3,8E-02 |
| P02545 | Prelamin-A/C | LMNA | 1,19 | 1,8E-02 |
| Q9BTT0-3 | Acidic leucine-rich nuclear phosphoprotein 32 family member E | ANP32E | 1,18 | 3,9E-02 |
| P28370-2 | Probable global transcription activator SNF2L1 | SMARCA1 | 1,18 | 7,5E-03 |
| P14174 | Macrophage migration inhibitory factor | MIF | 1,17 | 3,7E-02 |
| Q06830 | Peroxiredoxin-1 | PRDX1 | 1,17 | 1,3E-02 |
| O94905 | Erlin-2 | ERLIN2 | 1,17 | 4,0E-02 |
| P08134 | Rho-related GTP-binding protein RhoC | RHOC | 1,15 | 1,6E-02 |
| Q92598-2 | Heat shock protein 105 kDa | HSPH1 | 1,15 | 3,8E-02 |
| P49755 | Transmembrane emp24 domain-containing protein 10 | TMED10 | 1,14 | 3,5E-02 |
| Q15149-7 | Plectin | PLEC | 1,11 | 1,8E-02 |
| P35606-2 | Coatomer subunit beta | COPB2 | 1,11 | 2,7E-02 |
| P61160 | Actin-related protein 2 | ACTR2 | 1,08 | 3,5E-02 |
| Q03135 | Caveolin-1 | CAV1 | 0,95 | 6,6E-03 |
| Q6P1J9 | Parafibromin | CDC73 | 0,90 | 2,2E-02 |
| P07900 | Heat shock protein HSP 90-alpha | HSP90AA1 | 0,90 | 8,8E-03 |
| Q6P2Q9 | Pre-mRNA-processing-splicing factor 8 | PRPF8 | 0,87 | 1,2E-02 |
| Q8TD08-2 | Mitogen-activated protein kinase 15 | MAPK15 | 0,87 | 3,8E-02 |
| P82979 | SAP domain-containing ribonucleoprotein | SARNP | 0,86 | 5,0E-03 |
| P60842 | Eukaryotic initiation factor 4A-I | EIF4A1 | 0,85 | 4,7E-02 |
| P40123-2 | Adenylyl cyclase-associated protein 2 | CAP2 | 0,85 | 5,6E-03 |
| P78371 | T-complex protein 1 subunit beta | CCT2 | 0,85 | 6,0E-04 |
| Q04323 | UBX domain-containing protein 1 | UBXN1 | 0,84 | 3,9E-02 |
| P62826 | GTP-binding nuclear protein Ran | RAN | 0,83 | 2,6E-02 |
| P50990-2 | T-complex protein 1 subunit theta | CCT8 | 0,83 | 2,4E-02 |
| P62266 | 40S ribosomal protein S23 | RPS23 | 0,83 | 1,8E-02 |
| P49588 | Alanine--tRNA ligase, cytoplasmic | AARS | 0,83 | 3,0E-02 |
| Q99439-2 | Calponin-2 | CNN2 | 0,83 | 4,6E-02 |
| O95373 | Importin-7 | IPO7 | 0,82 | 4,0E-02 |
| P62820 | Ras-related protein Rab-1A | RAB1A | 0,82 | 9,5E-03 |
| P78527 | DNA-dependent protein kinase catalytic subunit | PRKDC | 0,82 | 1,9E-02 |
| Q05682-6 | Caldesmon | CALD1 | 0,81 | 4,6E-02 |
| Q08211 | ATP-dependent RNA helicase A | DHX9 | 0,80 | 2,7E-02 |
| P09651-3 | Heterogeneous nuclear ribonucleoprotein A1 | HNRNPA1 | 0,79 | 4,3E-02 |
| P33176 | Kinesin-1 heavy chain | KIF5B | 0,79 | 1,3E-02 |
| P34932 | Heat shock 70 kDa protein 4 | HSPA4 | 0,78 | 3,1E-02 |
| Q14566 | DNA replication licensing factor MCM6 | MCM6 | 0,78 | 1,6E-02 |
| P62424 | 60S ribosomal protein L7a | RPL7A | 0,77 | 1,3E-02 |
| Q32P28-4 | Prolyl 3-hydroxylase 1 | LEPRE1 | 0,77 | 4,0E-02 |
| Q9H0D6-2 | 5-3 exoribonuclease 2 | XRN2 | 0,77 | 4,6E-02 |
| P14209-3 | CD99 antigen | CD99 | 0,75 | 4,9E-03 |
| O00303 | Eukaryotic translation initiation factor 3 subunit F | EIF3F | 0,75 | 4,6E-02 |
| Q01082 | Spectrin beta chain, non-erythrocytic 1 | SPTBN1 | 0,75 | 1,5E-03 |
| P11388 | DNA topoisomerase 2-alpha | TOP2A | 0,74 | 2,6E-02 |
| O75821 | Eukaryotic translation initiation factor 3 subunit G | EIF3G | 0,74 | 1,8E-02 |
| P53992-2 | Protein transport protein Sec24C | SEC24C | 0,73 | 4,6E-02 |
| P58546 | Myotrophin | MTPN | 0,73 | 1,3E-02 |
| P55884 | Eukaryotic translation initiation factor 3 subunit B | EIF3B | 0,72 | 1,1E-02 |
| P17066 | Heat shock 70 kDa protein 6 | HSPA6 | 0,72 | 2,4E-02 |
| P46013-2 | Antigen KI-67 | MKI67 | 0,71 | 2,4E-02 |
| P08865 | 40S ribosomal protein SA | RPSA | 0,71 | 1,5E-03 |
| Q8WUM4 | Programmed cell death 6-interacting protein | PDCD6IP | 0,71 | 1,2E-04 |
| O43324-2 | Eukaryotic translation elongation factor 1 epsilon-1 | EEF1E1 | 0,70 | 5,0E-02 |
| P31948 | Stress-induced-phosphoprotein 1 | STIP1 | 0,70 | 1,4E-02 |
| Q9UHD1-2 | Cysteine and histidine-rich domain-containing protein 1 | CHORDC1 | 0,70 | 1,4E-02 |
| Q07955 | Serine/arginine-rich splicing factor 1 | SRSF1 | 0,70 | 4,7E-02 |
| Q13596-2 | Sorting nexin-1 | SNX1 | 0,70 | 4,5E-02 |
| P62258 | 14-3-3 protein epsilon | YWHAE | 0,69 | 6,0E-03 |
| P29966 | Myristoylated alanine-rich C-kinase substrate | MARCKS | 0,69 | 1,9E-02 |
| P55786-2 | Puromycin-sensitive aminopeptidase | NPEPPS | 0,68 | 2,6E-02 |
| Q15942 | Zyxin | ZYX | 0,68 | 1,9E-02 |
| Q14315-2 | Filamin-C | FLNC | 0,68 | 2,0E-02 |
| P55060-4 | Exportin-2 | CSE1L | 0,68 | 1,4E-03 |
| Q9NP79 | Vacuolar protein sorting-associated protein VTA1 homolog | VTA1 | 0,68 | 4,6E-02 |
| P12268 | Inosine-5-monophosphate dehydrogenase 2 | IMPDH2 | 0,67 | 1,4E-02 |
| Q13310-2 | Polyadenylate-binding protein 4 | PABPC4 | 0,66 | 4,2E-03 |
| Q15631 | Translin | TSN | 0,66 | 2,5E-02 |
| P51148 | Ras-related protein Rab-5C | RAB5C | 0,66 | 6,0E-03 |
| P62913-2 | 60S ribosomal protein L11 | RPL11 | 0,66 | 2,2E-03 |
| O43175 | D-3-phosphoglycerate dehydrogenase | PHGDH | 0,65 | 2,4E-02 |
| Q15637-4 | Splicing factor 1 | SF1 | 0,65 | 2,2E-02 |
| O00762-3 | Ubiquitin-conjugating enzyme E2 C | UBE2C | 0,65 | 9,3E-03 |
| P50479 | PDZ and LIM domain protein 4 | PDLIM4 | 0,64 | 8,3E-03 |
| Q14103-3 | Heterogeneous nuclear ribonucleoprotein D0 | HNRNPD | 0,63 | 1,5E-02 |
| Q14498-3 | RNA-binding protein 39 | RBM39 | 0,63 | 1,8E-02 |
| P20700 | Lamin-B1 | LMNB1 | 0,63 | 9,9E-03 |
| O75153 | Clustered mitochondria protein homolog | CLUH | 0,63 | 2,9E-02 |
| P26641 | Elongation factor 1-gamma | EEF1G | 0,62 | 7,1E-04 |
| Q9NP72-3 | Ras-related protein Rab-18 | RAB18 | 0,62 | 2,2E-02 |
| P07339 | Cathepsin D | CTSD | 0,61 | 2,5E-02 |
| P16402 | Histone H1.3 | HIST1H1D | 0,60 | 4,9E-03 |
| P19623 | Spermidine synthase | SRM | 0,60 | 4,7E-02 |
| Q8NDC0 | MAPK-interacting and spindle-stabilizing protein-like | MAPK1IP1L | 0,59 | 3,2E-03 |
| Q14019 | Coactosin-like protein | COTL1 | 0,59 | 7,0E-03 |
| Q9NQT5-2 | Exosome complex component RRP40 | EXOSC3 | 0,58 | 6,4E-04 |
| Q15366-7 | Poly(rC)-binding protein 2 | PCBP2 | 0,58 | 4,7E-02 |
| Q02790 | Peptidyl-prolyl cis-trans isomerase FKBP4 | FKBP4 | 0,58 | 3,5E-02 |
| P35080-2 | Profilin-2 | PFN2 | 0,57 | 2,8E-02 |
| Q9P2J5-3 | Leucine--tRNA ligase, cytoplasmic | LARS | 0,57 | 7,1E-03 |
| P55735-2 | Protein SEC13 homolog | SEC13 | 0,56 | 9,7E-04 |
| Q5IFJ8 | 26S proteasome non-ATPase regulatory subunit 5 | PSMD5 | 0,56 | 1,0E-02 |
| Q9Y3F4 | Serine-threonine kinase receptor-associated protein | STRAP | 0,56 | 2,1E-02 |
| P39023 | 60S ribosomal protein L3 | RPL3 | 0,56 | 2,1E-02 |
| O43399-6 | Tumor protein D54 | TPD52L2 | 0,56 | 4,5E-03 |
| Q13765 | Nascent polypeptide-associated complex subunit alpha | NACA | 0,55 | 2,0E-02 |
| P40429 | 60S ribosomal protein L13a | RPL13A | 0,55 | 2,0E-02 |
| Q9NW13-2 | RNA-binding protein 28 | RBM28 | 0,55 | 3,1E-03 |
| P46940 | Ras GTPase-activating-like protein IQGAP1 | IQGAP1 | 0,55 | 2,5E-02 |
| P07741-2 | Adenine phosphoribosyltransferase | APRT | 0,54 | 3,4E-02 |
| P46976-3 | Glycogenin-1 | GYG1 | 0,52 | 2,9E-02 |
| O43765 | Small glutamine-rich tetratricopeptide repeat-containing protein alpha | SGTA | 0,49 | 4,7E-02 |
| P35611-2 | Alpha-adducin | ADD1 | 0,49 | 1,4E-02 |
| Q6NZI2 | Polymerase I and transcript release factor | PTRF | 0,48 | 2,4E-02 |
| P63244 | Guanine nucleotide-binding protein subunit beta-2-like 1 | GNB2L1 | 0,47 | 1,8E-02 |
| Q9UHX1-4 | Poly(U)-binding-splicing factor PUF60 | PUF60 | 0,46 | 4,3E-03 |
| P11216 | Glycogen phosphorylase, brain form | PYGB | 0,46 | 4,2E-03 |
| Q9Y4B5-3 | Microtubule cross-linking factor 1 | MTCL1 | 0,44 | 1,5E-07 |
| P29144 | Tripeptidyl-peptidase 2 | TPP2 | 0,43 | 2,9E-02 |
| Q96H79-2 | Zinc finger CCCH-type antiviral protein 1-like | ZC3HAV1L | 0,42 | 6,6E-03 |
| Q15758-3 | Neutral amino acid transporter B(0) | SLC1A5 | 0,42 | 1,8E-02 |
| Q5VW32-2 | BRO1 domain-containing protein BROX | BROX | 0,41 | 3,5E-02 |
| P08123 | Collagen alpha-2(I) chain | COL1A2 | 0,39 | 1,5E-02 |
| Q15003-2 | Condensin complex subunit 2 | NCAPH | 0,39 | 8,5E-03 |
| Q96CS3 | FAS-associated factor 2 | FAF2 | 0,39 | 1,0E-02 |
| P55010 | Eukaryotic translation initiation factor 5 | EIF5 | 0,39 | 2,6E-02 |
| P02751-6 | Fibronectin | FN1 | 0,38 | 4,2E-04 |
| P84090 | Enhancer of rudimentary homolog | ERH | 0,37 | 4,6E-02 |
| Q9UNS2 | COP9 signalosome complex subunit 3 | COPS3 | 0,36 | 2,3E-02 |
| Q86WR7 | Proline and serine-rich protein 2 | PROSER2 | 0,35 | 2,0E-03 |
| P61221 | ATP-binding cassette sub-family E member 1 | ABCE1 | 0,33 | 5,0E-03 |
| Q9BPX3 | Condensin complex subunit 3 | NCAPG | 0,24 | 1,4E-03 |
| P49721 | Proteasome subunit beta type-2 | PSMB2 | 0,23 | 3,3E-02 |
| P61626 | Lysozyme C | LYZ | 0,20 | 1,8E-02 |
| Q9UBW8 | COP9 signalosome complex subunit 7a | COPS7A | 0,19 | 3,9E-02 |
| P28072 | Proteasome subunit beta type-6 | PSMB6 | 0,19 | 3,0E-03 |
| O14545 | TRAF-type zinc finger domain-containing protein 1 | TRAFD1 | 0,17 | 1,6E-02 |
| Q86Y82 | Syntaxin-12 | STX12 | 0,16 | 3,1E-02 |
| Q5JTV8-3 | Torsin-1A-interacting protein 1 | TOR1AIP1 | 0,15 | 4,9E-03 |
| P02452 | Collagen alpha-1(I) chain | COL1A1 | 0,15 | 6,7E-03 |
| P28074 | Proteasome subunit beta type-5 | PSMB5 | 0,09 | 1,7E-02 |
| O60888-3 | Protein CutA | CUTA | 0,04 | 4,9E-03 |
| Q12923-2 | Tyrosine-protein phosphatase non-receptor type 13 | PTPN13 | 0,02 | 4,5E-03 |
