# Supplementary material for: Co-Exposure of Cardiomyocytes to IFN-γ and TNF-α Induces Mitochondrial Dysfunction and Nitro-Oxidative Stress: Implications for the Pathogenesis of Chronic Chagas Disease Cardiomyopathy
Source: Front Immunol. 2021 Nov 11;12:755862. doi: 10.3389/fimmu.2021.755862 (PMC8632642; doi:10.3389/fimmu.2021.755862)
Supplement: Supplementary file 7 [file Table_2.docx]

**Supplementary Table 2:** List of DEGs (non stimulated versus IFNγ+TFNα stimulated AC16 cell line) at t=1h, 6h, 12h, 24h, 48h.

| **Time points** | **Types of sequence** | **DEGs (Name (FC; adjusted p value))** |
| --- | --- | --- |
| **T=1h**  **214 DEGs** | protein_coding | **Down:** CHAC1 (FC: -2,94; Adj pval: 5,01E-03), TMEM177 (-2,83; 5,54E-15), ZNF572 (-5,17; 4,00E-13), ZNF627 (-3,17; 1,67E-16)  **Up:** ABL2 (5,23; 1,99E-46), ACAN (5,58; 3,92E-07), ACPT (3,26; 3,17E-06), ADAMTS1 (3,44; 1,78E-14), ADRB2 (4,07; 1,54E-06), AIM2 (3,21; 9,81E-05), AL353583,1 (5,69; 1,95E-07), AL590452,1 (3,02; 1,31E-04), APOL4 (5,92; 8,74E-16), APOL6 (3,24; 3,67E-25), ATF3 (3,99; 1,05E-26), BATF2 (6,45; 5,16E-15), BCL6 (3,93; 2,77E-42), BDKRB1 (4,68; 5,80E-11), BHLHE40 (3,47; 2,20E-09), C11orf96 (5,77; 4,92E-11), C20orf141 (5,98; 2,86E-09), C5orf56 (4,54; 3,14E-17), C8orf46 (3,21; 1,02E-09), C9orf131 (4,00; 1,88E-06), CAPN12 (4,86; 2,22E-09), CCDC64 (2,86; 7,79E-04), CCDC64B (9,01; 4,70E-13), CCDC85B (4,02; 2,58E-08), CCL2 (5,15; 2,98E-13), CCNL1 (3,30; 2,87E-46), CCRN4L (3,61; 5,33E-34), CLDN5 (2,92; 1,77E-03), CPEB2 (2,89; 8,22E-48), CRB2 (3,19; 2,01E-03), CSRNP1 (5,07; 6,72E-25), CXCL1 (3,43; 3,20E-06), CXCL2 (4,92; 1,49E-20), CYP21A2 (3,01; 5,97E-03), CYP26B1 (3,68; 6,68E-04), CYP2A6 (2,85; 9,63E-03), DDC8 (3,44; 4,49E-07), DLX2 (3,94; 1,81E-06), DUSP5 (4,97; 1,43E-20), EFCAB12 (3,27; 7,39E-04), EGR2 (6,08; 3,61E-09), EGR3 (12,37; 4,19E-33), ERRFI1 (3,81; 8,47E-19), FAM222A (2,83; 6,48E-04), FAM46A (3,40; 2,80E-95), FAM46C (11,11; 3,33E-30), FAM65C (3,56; 1,42E-07), FBXO32 (3,14; 1,79E-09), FOSB (4,48; 6,04E-10), FOXC1 (3,00; 8,59E-18), GBP1 (4,30; 4,21E-18), GBP2 (3,28; 1,41E-09), GEM (7,06; 9,05E-52), GNAT2 (4,33; 2,52E-10), HBEGF (2,91; 2,63E-09), HES1 (2,92; 3,98E-03), ICAM1 (2,84; 8,40E-06), ID2 (3,93; 1,62E-34), IFIT3 (4,11; 1,32E-23), IL16 (3,78; 9,20E-07), IL8 (2,83; 1,05E-05), IRF1 (10,07; 6,37E-50), IRF9 (3,19; 1,46E-08), IRS2 (3,70; 1,60E-24), ITK (3,22; 2,71E-05), KDM6B (3,67; 8,21E-49), KLF10 (4,86; 2,60E-56), KLF4 (8,70; 1,93E-60), KLF5 (3,41; 4,76E-26), KLF9 (3,29; 9,10E-20), KLHL15 (3,77; 1,70E-55), LAG3 (3,16; 1,10E-07), LIF (3,23; 6,51E-10), LMOD2 (2,94; 4,03E-02), LRRTM2 (3,13; 2,62E-05), LSAMP (5,15; 2,63E-07), LSMEM2 (2,97; 3,28E-04), MAFB (3,45; 8,52E-04), MAFF (5,36; 3,40E-22), MAP3K8 (4,83; 3,27E-49), MISP (3,91; 4,58E-06), MMP25 (4,18; 1,21E-05), MSANTD1 (4,04; 5,51E-05), MUC17 (2,90; 7,96E-03), MX1 (3,40; 1,27E-07), MYC (3,00; 2,15E-11), MYLK2 (3,74; 5,56E-07), NABP1 (3,40; 7,62E-26), NEDD9 (3,08; 2,07E-17), NFKBIA (5,09; 5,70E-32), NR4A1 (2,90; 4,80E-10), NR4A2 (7,54; 8,03E-18), NR4A3 (18,42; 5,15E-54), OSR2 (3,15; 7,43E-09), PAQR5 (2,94; 1,74E-05), PER2 (3,32; 2,01E-10), PILRA (3,06; 2,15E-04), PIM1 (3,20; 2,02E-102), PLEKHG6 (3,96; 1,77E-10), PRDM1 (3,27; 2,00E-13), PRKCG (3,24; 2,82E-03), PROZ (3,66; 5,85E-05), PTGER4 (6,65; 2,97E-56), PYGM (5,13; 1,79E-09), REC8 (3,14; 3,73E-09), RGCC (4,00; 2,60E-05), RND1 (4,45; 2,02E-08), RND3 (4,52; 7,58E-22), RP11-468E2,4 (3,41; 6,70E-07), RP11-766F14,2 (6,58; 2,25E-16), SCHIP1 (5,35; 1,02E-13), SELPLG (3,13; 5,04E-05), SEMA4A (4,88; 5,59E-14), SERTAD1 (2,99; 3,69E-08), SGK1 (4,51; 1,30E-10), SIK1 (2,95; 2,60E-19), SLC25A25 (4,23; 1,66E-39), SMTNL1 (4,89; 1,14E-08), SOCS1 (3,84; 1,24E-08), SOCS3 (6,06; 1,43E-42), SPRY4 (2,91; 2,02E-07), ST7-OT4 (2,84; 1,86E-04), STAB1 (4,89; 6,98E-07), TACR2 (3,32; 2,34E-07), TAP1 (2,88; 3,16E-15), TCF7 (2,91; 1,08E-09), TLDC2 (2,84; 6,87E-06), TMEM239 (7,95; 4,92E-11), TMEM88 (5,24; 8,63E-14), TNFAIP3 (3,31; 9,82E-06), TRIB1 (13,34; 9,96E-77), WNT4 (3,29; 1,95E-04), XIRP1 (6,11; 1,41E-13), ZC3H12A (3,25; 2,25E-06) |
|  | lincRNA | **Up:** AC017002,2 (3,40; 7,27E-10), CTD-2105E13,13 (2,93; 3,62E-04), KB-1732A1,1 (5,54; 1,34E-13), LINC00312 (4,43; 9,53E-11), LUCAT1 (3,12; 4,98E-04), MIR145 (3,85; 8,87E-08), MIR194-2 (4,03; 5,92E-05), MIR22HG (2,98; 5,60E-13), RP11-131L23,2 (3,14; 2,34E-03), RP11-154H12,2 (4,03; 5,10E-07), RP11-290F5,1 (3,54; 8,60E-04), RP11-333O1,1 (3,10; 9,76E-04), RP11-375I20,6 (6,20; 1,21E-12), RP11-383J24,1 (2,86; 1,14E-03), RP11-47I22,2 (3,04; 2,25E-05), RP11-557H15,3 (3,23; 9,62E-06), RP4-564F22,5 (3,32; 9,30E-05) |
|  | snRNA | **Down:** RNVU1-6 (-3,38; 1,58E-07)  **Up:** RNU7-40P (2,93; 2,45E-03) |
|  | pseudogene | **Down:** AC079781,5 (-3,35; 1,03E-03)  **Up:** AC005682,6 (3,26; 5,03E-06), AC010492,2 (2,96; 1,93E-03), AC068491,3 (4,78; 7,16E-08), AC133644,3 (3,81; 1,55E-05), AP000593,6 (2,83; 9,32E-03), BLZF2P (4,73; 7,37E-07), ETF1P2 (3,05; 2,20E-05), FTH1P4 (3,05; 5,09E-04), KRT8P42 (4,34; 1,94E-04), RP11-16E23,4 (3,64; 1,14E-04), RP11-28B23,1 (3,51; 4,54E-04), RP11-486B10,4 (3,85; 1,03E-05), RP11-638I2,10 (3,17; 1,14E-04), RP11-670E13,3 (4,27; 2,60E-05), RP11-739N20,3 (3,48; 7,16E-04), RP11-745A24,1 (3,37; 3,40E-10), RP11-807E13,2 (3,22; 1,92E-03), RP5-916O11,3 (2,86; 3,84E-03), RPL3P6 (3,84; 1,42E-04), TNXA (3,62; 4,06E-04), UBE2FP1 (2,92; 2,26E-05) |
|  | antisense | **Up:** AC006159,5 (5,70; 9,22E-11), AC006160,5 (4,58; 2,55E-08), AC006460,2 (3,09; 1,14E-03), AC007362,3 (4,44; 7,15E-08), AC007750,5 (4,41; 5,59E-09), AC012360,6 (3,37; 4,49E-10), AC116366,6 (6,69; 9,17E-19), COL5A1-AS1 (3,09; 7,09E-04), CTC-308K20,1 (2,87; 2,61E-04), CTD-2031P19,3 (3,60; 2,06E-06), IDI2-AS1 (2,92; 3,88E-03), IQCJ-SCHIP1-AS1 (3,31; 6,55E-05), RP11-114F10,3 (3,28; 1,15E-04), RP11-131L23,1 (3,30; 3,20E-04), RP11-1399P15,1 (3,80; 7,81E-06), RP11-286E11,1 (3,44; 7,77E-04), RP11-473M20,5 (11,94; 1,21E-13), RP11-506B6,6 (3,71; 1,41E-04), RP11-758P17,3 (3,51; 3,07E-04), RP11-78A19,4 (4,11; 2,17E-04), RP1-50J22,4 (3,92; 1,43E-05), RP1-68D18,4 (4,87; 1,20E-09), RP3-416H24,1 (2,84; 1,21E-03), RP4-657D16,3 (4,03; 3,67E-15), ZRANB2-AS1 (3,18; 1,06E-04) |
|  | processed_transcript | **Up:** MIR17HG (4,13; 1,92E-09) |
|  | sense_overlapping | **Up:** CTD-3247F14,2 (8,00; 2,10E-08), RP11-448G15,3 (4,34; 8,07E-20), RP11-552M11,4 (3,18; 1,56E-04) |
|  | sense_intronic | **Up:** AP000240,9 (3,88; 5,27E-07), RP11-16E23,3 (3,01; 1,38E-03), RP11-359K18,4 (2,91; 4,51E-04), RP11-421E14,2 (3,09; 8,48E-05), SIK3-IT1 (3,11; 3,00E-05) |
|  | misc_RNA | **Up:** Y_RNA (3,39; 1,46E-05) |
|  |  |  |
| **T=6h**  **858 DEGs** | protein_coding | **Down:** AARS (-2,9; 9,80E-11), ABCG4 (-4,02; 3,00E-09), ACACB (-3,1; 2,42E-13), ACBD4 (-3,19; 1,99E-07), ACBD7 (-2,89; 7,60E-08), ADAM22 (-2,85; 5,00E-15), ADM2 (-5,78; 4,25E-10), ADORA1 (-3,19; 9,40E-07), AJUBA (-4,14; 1,30E-16), AKNA (-3,17; 3,20E-08), AMIGO1 (-4,93; 3,00E-12), ANKRD34A (-3,52; 3,61E-19), ANXA9 (-3,3; 5,70E-05), APBA1 (-2,92; 6,85E-04), ARHGAP9 (-2,99; 8,80E-05), ARVCF (-3,99; 7,58E-10), BMP4 (-4,3; 2,23E-16), C11orf87 (-8,66; 1,88E-16), C15orf65 (-4,13; 3,20E-08), C1orf134 (-3,34; 2,00E-06), CALCRL (-3,08; 2,23E-07), CARF (-2,9; 7,43E-10), CBX7 (-2,97; 2,14E-07), CD200 (-3,39; 2,31E-07), CELSR2 (-2,86; 7,19E-13), CHAC1 (-5,72; 2,00E-09), CITED2 (-2,84; 5,93E-10), CLCN4 (-2,88; 1,00E-21), CORO6 (-2,84; 8,00E-06), CSRNP3 (-5,69; 4,63E-19), CTGF (-4,06; 4,31E-13), CTH (-5,76; 1,83E-13), CXADR (-3; 5,00E-09), CYP2S1 (-2,89; 3,90E-11), CYS1 (-3,37; 9,73E-07), DAAM2 (-3,25; 1,50E-11), DBP (-7,84; 9,00E-30), DDIT4 (-2,86; 2,46E-04), DENND2A (-3,11; 9,30E-17), DMGDH (-3,08; 1,10E-05), DNM3 (-2,98; 6,00E-11), DUSP1 (-4; 1,70E-11), ECM2 (-5,07; 5,94E-10), EGR1 (-5,96; 3,00E-15), EHHADH (-4,23; 1,00E-24), EPOR (-3,21; 1,73E-10), ERBB3 (-6,41; 3,30E-20), ESRRG (-4,8; 2,00E-06), EXPH5 (-4,57; 8,30E-20), F2RL2 (-3,74; 2,00E-24), FAM117A (-3,52; 5,65E-16), FAM124A (-6,28; 4,81E-13), FAM13C (-3,35; 4,00E-05), FAM182B (-3,36; 4,93E-07), FAM198B (-3,74; 1,00E-18), FAM57B (-3,56; 1,00E-05), FAM63A (-3,09; 2,65E-16), FAXDC2 (-3,99; 5,00E-12), FBXL20 (-2,86; 2,00E-15), FILIP1 (-3,26; 1,47E-07), FLRT1 (-3,86; 8,00E-06), FOS (-23,19; 4,04E-34), FOSB (-9,5; 1,80E-23), FOXO6 (-2,98; 2,00E-06), GAB2 (-3,36; 1,60E-17), GDF15 (-3,65; 2,00E-06), GDPD1 (-4,42; 2,80E-17), GLRB (-4,22; 2,00E-18), GOLGA6L20 (-3,38; 1,40E-05), GPR155 (-3,69; 1,70E-17), GPR162 (-3,01; 8,10E-20), GPT2 (-3,21; 4,00E-09), GRAMD2 (-2,86; 2,30E-03), GYG2 (-4,12; 3,34E-13), HOXA6 (-3,42; 7,20E-05), HOXB9 (-3,17; 6,00E-14), HPSE (-3,27; 2,00E-15), ID1 (-4,67; 3,00E-12), IL20RB (-3,83; 1,00E-06), IL21R (-2,86; 3,34E-04), IMPA2 (-3; 4,23E-10), INHBE (-9,58; 1,54E-16), IP6K3 (-3,52; 4,00E-06), IRAK1BP1 (-3,05; 1,00E-12), JAG2 (-3,32; 6,00E-06), JDP2 (-3,42; 1,62E-16), JMY (-4,65; 6,50E-32), KCNH7 (-3,28; 2,23E-04), KCTD16 (-3,62; 6,00E-06), KIAA0319 (-3,02; 1,06E-04), KIAA1211 (-2,87; 9,00E-06), KIAA1467 (-3,18; 6,54E-22), KIT (-7,33; 1,19E-19), KLF2 (-5,62; 7,00E-21), KLHL24 (-4,21; 2,00E-18), LCA5 (-2,91; 5,09E-19), LIN7B (-3; 1,30E-05), LMOD1 (-5,19; 1,65E-13), LOH12CR2 (-4,48; 1,30E-05), LRRC63 (-3,69; 2,03E-07), LRRC7 (-2,93; 2,70E-08), LURAP1L (-3,65; 1,53E-07), LYPD6B (-3,85; 5,00E-09), LZTS3 (-3,17; 6,00E-15), MAMSTR (-3,48; 9,90E-14), MAN1C1 (-4,3; 5,85E-10), MANSC1 (-2,87; 1,52E-07), MAP2K6 (-4,6; 7,00E-09), METTL7A (-3,38; 1,00E-06), MGARP (-2,92; 6,59E-04), MGAT3 (-3,52; 2,30E-05), MID1IP1 (-3,8; 1,75E-19), MITF (-3,08; 3,04E-19), MKX (-5,4; 6,29E-16), MORN4 (-4,91; 4,80E-23), MROH8 (-3,16; 6,00E-06), MRVI1 (-3,4; 2,30E-05), MVK (-2,85; 4,34E-13), MXD3 (-3,1; 6,00E-12), MYCL (-3,07; 4,70E-08), MYEF2 (-4,1; 4,00E-12), NFE2 (-6,12; 5,00E-12), NFIA (-2,86; 3,02E-16), NR1D1 (-3,67; 5,00E-29), NR4A1 (-6,87; 9,85E-31), NSUN7 (-3,43; 1,00E-06), NYAP1 (-2,95; 3,36E-03), NYNRIN (-3,48; 4,60E-26), OCLN (-3,22; 4,20E-14), OPRL1 (-3,14; 4,16E-04), OSR2 (-3,98; 6,00E-12), PARD6B (-2,99; 1,45E-13), PDP2 (-3,4; 6,00E-27), PGPEP1 (-4,18; 1,00E-24), PHOSPHO2 (-2,84; 1,60E-05), PIK3C2B (-6,04; 1,20E-29), PKP2 (-5,51; 3,66E-10), PLEKHA6 (-3,71; 3,20E-08), PLEKHH2 (-2,85; 7,80E-20), PPAPDC3 (-2,85; 8,04E-04), PPARGC1A (-6,88; 7,11E-13), PPM1E (-3,83; 3,00E-09), PRICKLE1 (-6,92; 5,00E-33), PSAT1 (-3,76; 4,50E-08), PTCH1 (-2,92; 4,00E-18), PTK2B (-4,06; 4,00E-12), PTPN13 (-3,27; 3,03E-19), PURG (-4,16; 2,50E-08), RAB11FIP1 (-3,64; 1,10E-20), RAB40B (-3,02; 9,00E-09), RASSF2 (-3,06; 7,90E-05), RASSF7 (-3,29; 5,09E-10), REEP1 (-3,15; 3,49E-10), RFXAP (-3,61; 8,10E-20), RGS9 (-2,85; 2,09E-04), RHOB (-3,44; 2,00E-18), RHPN1 (-3,3; 1,71E-04), RIMS3 (-4,13; 2,40E-08), RORA (-3,49; 1,98E-07), RTN4R (-4,36; 2,80E-05), S1PR1 (-3,02; 1,00E-05), SALL2 (-3; 8,50E-29), SAMD12 (-3,08; 4,00E-15), SDPR (-5,39; 4,00E-12), SESN2 (-2,99; 2,00E-06), SESN3 (-3,6; 4,10E-17), SGCG (-3,52; 4,10E-05), SH2D3C (-3,96; 2,00E-06), SIAH3 (-4,18; 3,42E-07), SLC16A14 (-5,16; 1,30E-16), SLC22A15 (-3,32; 6,70E-05), SLC25A42 (-3,28; 5,55E-19), SLC2A12 (-2,84; 6,00E-05), SLC38A4 (-2,9; 3,00E-09), SLC40A1 (-3,15; 1,56E-10), SLC46A3 (-3,37; 3,40E-29), SLC7A11 (-4,6; 6,00E-09), SLC7A8 (-4,43; 1,34E-07), SLCO4C1 (-3,08; 4,04E-04), SLITRK3 (-4,15; 1,80E-05), SLITRK5 (-4,46; 2,79E-16), SMAD9 (-3,31; 1,56E-16), SMO (-2,84; 5,08E-10), SPEG (-3,25; 9,60E-35), SPRY1 (-2,93; 1,51E-04), SSBP2 (-3,99; 2,60E-17), STEAP2 (-3,05; 2,00E-06), STON1 (-3,68; 1,30E-11), SYBU (-5,19; 5,94E-10), SYDE2 (-3,74; 6,04E-28), SYT1 (-3,19; 5,00E-15), TET1 (-3,26; 2,56E-19), THAP8 (-3,16; 4,10E-08), TMEM169 (-3,72; 1,00E-15), TMEM170B (-5,84; 5,70E-32), TMEM178B (-3,44; 2,70E-05), TMEM187 (-5,36; 5,13E-13), TMEM246 (-2,93; 1,51E-19), TMEM56 (-3,41; 4,11E-19), TOX (-2,84; 4,20E-05), TRIB3 (-5,81; 1,00E-12), TRIM6 (-3,18; 1,00E-12), TSC22D3 (-2,97; 1,10E-05), TTC9 (-2,86; 3,65E-04), TUB (-2,83; 6,49E-16), TYRP1 (-3,05; 4,19E-04), ULBP1 (-4,03; 5,40E-14), USP2 (-3,3; 5,50E-08), VLDLR (-5,06; 4,00E-18), YPEL1 (-4,51; 1,80E-08), YPEL3 (-3,1; 7,01E-10), ZBED3 (-3,75; 6,13E-19), ZBTB8B (-3,05; 3,72E-04), ZC3H12B (-2,87; 1,71E-10), ZC3H6 (-4,3; 3,09E-13), ZNF25 (-3,38; 1,75E-13)  **Up:** ABI3 (2,89; 3,98E-07), ABTB2 (3,1; 6,30E-17), AC090673,2 (4,16; 2,00E-06), ACAN (9,17; 8,30E-11), ACO1 (2,88; 2,00E-51), ACSL5 (4,48; 5,54E-10), ADAP1 (5,51; 9,88E-10), AIM1 (3,02; 7,34E-112), AIM2 (7,87; 7,39E-10), AKR1B1 (3,43; 5,60E-14), AL590452,1 (3,04; 2,70E-05), ALCAM (2,94; 1,25E-25), AMPD3 (4,49; 3,20E-11), AMZ1 (3,14; 1,88E-07), ANGPTL4 (4,41; 2,94E-07), ANKRD55 (2,97; 1,17E-04), ANO4 (5,46; 9,00E-28), ANTXR2 (3,18; 6,00E-12), ANXA10 (2,95; 1,59E-04), ANXA8 (5,14; 3,30E-10), ANXA8L1 (4,08; 4,00E-06), ANXA8L2 (5,16; 6,00E-12), AP000695,1 (2,88; 3,55E-04), APCDD1L (3,2; 1,00E-06), APOL1 (6,14; 9,50E-44), APOL2 (7,5; 1,00E-51), APOL3 (11,08; 1,68E-61), APOL4 (37,57; 3,43E-64), APOL6 (9,25; 6,00E-93), ARHGAP31 (2,89; 2,80E-20), ARHGEF16 (3,85; 1,04E-07), ARID5A (3,67; 1,70E-17), ASB2 (4,95; 9,00E-06), ATP2B1 (3,98; 2,90E-77), B4GALT1 (3,09; 5,00E-54), BATF2 (24,88; 1,97E-43), BATF3 (4,56; 4,24E-40), BCL2A1 (3,32; 1,33E-03), BCL3 (5,45; 2,40E-98), BDKRB1 (13,41; 1,42E-31), BDKRB2 (4,84; 2,00E-12), BEST3 (3,87; 9,55E-07), BID (4,95; 2,90E-35), BIRC3 (11,69; 4,68E-25), BMPER (3,58; 3,09E-07), BST2 (3,52; 2,00E-09), C11orf96 (3,27; 6,00E-06), C1orf147 (3,61; 4,00E-06), C1orf195 (3,04; 1,62E-04), C1QTNF1 (23,88; 1,55E-40), C1S (3,77; 4,32E-22), C20orf141 (8,34; 4,20E-13), C3 (6,45; 3,00E-39), C5orf56 (2,91; 4,00E-09), C8orf46 (3,55; 4,00E-12), C9orf47 (5,33; 7,92E-10), CAMK1G (6,07; 5,00E-09), CASP7 (3,04; 1,00E-30), CAV1 (3,38; 4,00E-36), CCDC3 (3,43; 4,90E-17), CCDC71L (3,26; 2,30E-14), CCDC85B (5,15; 3,00E-12), CCL13 (3,4; 2,00E-06), CCL2 (47,44; 5,80E-74), CCL7 (7,23; 4,00E-11), CCL8 (3,27; 1,35E-04), CCND1 (5,33; 9,55E-10), CCRN4L (3,23; 6,90E-29), CD274 (7,78; 1,72E-19), CD44 (4,08; 2,57E-13), CD47 (3,2; 2,47E-37), CD7 (4,55; 1,30E-07), CD70 (6,9; 1,37E-16), CD74 (5,12; 6,50E-11), CD82 (4,41; 4,01E-10), CD83 (8,19; 4,20E-29), CDCP1 (5,46; 1,80E-14), CDK6 (3,37; 6,10E-23), CFB (6; 1,20E-17), CFLAR (3,5; 1,00E-27), CHST15 (7,46; 8,30E-29), CIITA (48,11; 4,00E-59), CLDN1 (6,77; 1,00E-15), CLEC2B (3,24; 2,50E-14), CLEC2D (3,74; 3,70E-11), CLIC2 (6,24; 1,30E-20), CMPK2 (4,81; 2,30E-08), COL17A1 (4,86; 6,00E-09), COL27A1 (7,12; 1,71E-22), COL5A3 (3,27; 1,80E-05), COL7A1 (4,14; 1,00E-09), CRISPLD2 (3,24; 3,00E-09), CSF1 (10,71; 1,30E-125), CSF2 (3,79; 4,10E-05), CTC-479C5,12 (5,53; 2,00E-15), CTD-2547L24,3 (3,46; 4,50E-05), CTSS (19,91; 2,00E-42), CUBN (3,66; 9,10E-11), CX3CL1 (3,88; 1,26E-04), CXCL1 (19,08; 1,20E-29), CXCL10 (42,42; 1,60E-40), CXCL11 (24,25; 2,50E-29), CXCL2 (8,08; 2,00E-36), CXCL3 (9,05; 3,77E-22), CXCL5 (5; 6,42E-10), CXCL9 (7,45; 4,70E-20), DDX58 (8,23; 4,00E-63), DEC1 (4,35; 1,84E-07), DGKI (3,5; 7,00E-06), DNAH17 (3,05; 1,00E-08), DNAJA4 (3,05; 2,88E-04), DNPEP (2,89; 1,00E-36), DOCK10 (3,53; 4,00E-33), DRAM1 (7,47; 2,90E-53), DTX3L (5,23; 1,36E-79), EBI3 (3,52; 2,00E-12), EDARADD (2,96; 7,00E-09), EDNRA (2,92; 5,00E-06), EFHD2 (4,48; 5,80E-41), EFNA1 (5,86; 2,08E-10), EGFR (3,64; 5,60E-35), EHD1 (5,05; 6,00E-24), ELOVL7 (4,02; 6,20E-32), EML1 (3,43; 1,00E-12), ENC1 (3,1; 3,09E-10), EPSTI1 (6,7; 2,20E-41), ETS1 (3,91; 2,70E-29), ETS2 (3,56; 1,00E-36), ETV7 (16,64; 5,00E-54), EVA1A (4,94; 3,61E-19), F3 (3,28; 2,00E-06), FAM101A (10,24; 3,90E-29), FAM115C (5,22; 2,30E-14), FAM177B (4,15; 3,10E-05), FAM180A (4,66; 1,50E-08), FAM19A3 (4,63; 9,96E-07), FAM20C (3,51; 1,80E-17), FAM43A (4,77; 4,00E-09), FBXO32 (3,65; 6,82E-13), FBXO6 (3,52; 1,50E-05), FCRL6 (3,2; 1,80E-05), FDCSP (3,3; 3,91E-07), FLT1 (5,1; 2,25E-07), FOSL1 (4,97; 6,00E-12), FOXC2 (6,49; 1,00E-24), FST (7,96; 1,46E-37), G0S2 (11,29; 1,25E-25), GBP1 (39,05; 6,10E-119), GBP2 (47,13; 4,16E-106), GBP3 (12,36; 1,05E-73), GBP4 (56,29; 1,94E-61), GBP5 (35,74; 8,70E-50), GCH1 (6,28; 5,00E-41), GFPT2 (7,36; 8,00E-18), GGT5 (3,59; 9,00E-06), GIMAP2 (3,12; 9,40E-05), GPR115 (3,92; 1,00E-06), GPR158 (3,48; 6,00E-12), GPR56 (5,47; 1,00E-12), GPR68 (9,15; 4,00E-15), GPRC5B (7,14; 1,00E-30), GPX3 (3,28; 6,44E-19), GREM1 (3,74; 3,50E-11), GSAP (3,49; 1,00E-16), HAPLN3 (11,01; 4,00E-51), HAS2 (3,77; 9,30E-07), HDAC9 (4,62; 1,00E-38), HECW2 (4,18; 2,70E-26), HELZ2 (4,16; 5,20E-32), HIC1 (3,84; 3,68E-13), HIVEP1 (3,51; 2,00E-45), HIVEP2 (4,1; 4,85E-79), HK2 (3,04; 3,78E-19), HLA-E (3,39; 7,70E-29), HLA-F (3,64; 1,70E-14), HMGA2 (4,3; 3,00E-09), HRH1 (3,21; 3,00E-27), HS3ST3B1 (5,46; 6,00E-24), HSPH1 (2,98; 2,00E-12), ICAM1 (22,78; 2,10E-53), ICOSLG (7,64; 8,20E-20), IDO1 (42,45; 3,00E-45), IER3 (3,71; 3,00E-21), IFI35 (3,52; 2,00E-48), IFI44L (6,37; 1,50E-16), IFIH1 (4,47; 2,00E-27), IFIT3 (11,99; 4,00E-75), IFIT5 (4,51; 6,70E-68), IFN+TNFAR2 (2,97; 2,00E-30), IFN+TNFGR2 (3,28; 9,80E-26), IKBKE (4,45; 4,94E-28), IL12A (3,19; 9,08E-10), IL15 (3,64; 2,00E-12), IL15RA (14,66; 1,00E-45), IL18BP (6,22; 7,00E-57), IL1A (3,55; 6,41E-07), IL1B (22,1; 1,00E-29), IL32 (24,61; 9,00E-60), IL33 (3,13; 1,75E-03), IL4R (4,74; 3,90E-32), IL6 (12,26; 7,10E-22), IL7 (3,99; 6,00E-06), IL7R (6,1; 9,10E-23), IL8 (29,61; 9,70E-38), INHBA (17,51; 7,90E-29), IRAK2 (15,95; 2,00E-47), IRF1 (22,14; 3,55E-91), IRF2 (4,56; 1,00E-57), IRF9 (3,43; 2,86E-10), ISG20 (4,39; 1,56E-10), ITGB6 (2,92; 4,11E-04), ITK (16,62; 2,00E-15), ITPRIPL2 (3,2; 1,32E-25), JAK2 (3; 1,70E-23), KANK1 (3,04; 4,90E-08), KCNJ15 (7,67; 4,14E-13), KCTD12 (2,87; 1,42E-07), KDR (5,24; 1,20E-08), KIAA0247 (3,64; 6,92E-31), KIAA1199 (2,9; 3,00E-06), KIAA1217 (3,99; 1,00E-57), KIAA1644 (8,98; 3,00E-30), KLF17 (3,58; 1,80E-05), KYNU (3,44; 2,00E-05), LACTB (2,97; 3,00E-21), LAMB3 (11,31; 1,37E-31), LAMC2 (6,54; 3,30E-25), LAP3 (6,08; 4,50E-95), LGALS9 (3,08; 8,80E-05), LHFPL2 (3,3; 6,24E-64), LIF (6,49; 3,20E-26), LIMD2 (2,89; 1,00E-09), LIPG (4,19; 2,00E-06), LOX (3; 4,52E-22), LPXN (3,51; 3,00E-09), LRRC32 (5,73; 1,49E-19), LRRN3 (4,84; 7,40E-08), LRRTM2 (4,24; 5,00E-09), MAP2K3 (5,64; 3,82E-16), MAP3K5 (2,95; 8,53E-37), MDGA1 (3,06; 3,54E-13), MEFV (6,91; 4,00E-09), MEOX1 (4,59; 7,28E-07), METTL1 (2,88; 1,95E-16), MFHAS1 (3,05; 5,23E-58), MFSD2A (3,35; 4,00E-15), MGLL (3,49; 5,00E-15), MLKL (6,57; 1,71E-22), MRPS24 (5,29; 3,00E-12), MSC (11,11; 2,20E-47), MSX1 (3,64; 1,90E-17), MT2A (4,87; 3,10E-14), MTSS1 (6,65; 5,00E-45), MUC12 (2,91; 2,39E-03), MX1 (8,45; 3,00E-24), MX2 (4,42; 2,50E-08), MYEOV (3,83; 6,76E-07), MYO10 (5,06; 2,00E-17), MYPN (5,21; 3,50E-11), NAMPT (3,27; 5,90E-35), NAV2 (4,87; 6,00E-18), NAV3 (2,97; 1,60E-14), NEDD9 (4,71; 3,42E-34), NFATC2 (6,56; 7,40E-11), NFE2L3 (3,96; 2,15E-16), NFKB1 (7,81; 5,00E-117), NFKB2 (5,45; 8,50E-47), NFKBIA (11,79; 4,00E-75), NFKBIE (5,56; 1,00E-24), NGF (3,09; 1,90E-08), NHSL2 (3,42; 8,40E-08), NINJ1 (4,41; 2,01E-34), NKX3-1 (9,95; 1,00E-59), NLRC5 (5,83; 1,30E-122), NLRP3 (4,54; 6,00E-07), NMI (5,1; 6,82E-67), NPTX1 (5,77; 6,00E-09), NPY4R (3,99; 1,40E-05), NRG1 (4,54; 7,80E-22), NRP1 (3,36; 2,00E-36), NRP2 (8,88; 2,00E-57), NUAK2 (4,85; 5,35E-28), NUBP1 (3,01; 3,38E-10), OAS1 (3,86; 2,50E-07), OAS2 (9,3; 1,20E-22), OAS3 (5,39; 8,40E-41), OASL (3,41; 1,10E-08), ODF3B (3,65; 4,00E-06), OGFR (3,41; 1,70E-26), OLR1 (3,92; 3,40E-07), OTUD4 (3,19; 2,02E-46), PANX1 (4,57; 2,40E-50), PAPPA (3,2; 8,00E-09), PAQR9 (3,98; 1,40E-05), PARP12 (3,98; 1,40E-56), PARP14 (11,62; 1,19E-91), PARP9 (3,28; 3,00E-27), PAX8 (7,73; 7,60E-20), PCDH7 (6,5; 6,50E-14), PDCD1LG2 (3,87; 1,97E-10), PDGFA (4,53; 1,00E-21), PDLIM4 (4,97; 1,00E-33), PHACTR1 (8,38; 5,00E-32), PHLDA1 (3,33; 1,00E-12), PIK3CD (4,37; 8,20E-38), PLAU (3,36; 1,00E-10), PLAUR (4,2; 4,00E-15), PLSCR1 (3,2; 4,93E-46), PML (5,38; 4,30E-92), POU2F2 (5,31; 1,89E-16), PPAP2B (6,91; 6,50E-44), PPIF (5,65; 9,85E-31), PPP1R18 (2,99; 1,50E-32), PPP3CC (2,99; 4,99E-28), PPP4R4 (4,04; 2,20E-05), PRDM1 (3,15; 2,86E-13), PRDM8 (3,98; 5,50E-31), PSMB10 (4,17; 1,07E-16), PSMB8 (3,11; 3,45E-19), PSMB9 (4,58; 2,09E-31), PSME2 (2,9; 2,00E-72), PTAFR (2,94; 1,00E-05), PTHLH (3,11; 8,00E-06), PTPRE (3,74; 1,50E-11), PTX3 (10,41; 2,00E-27), RAB27B (4,74; 4,00E-18), RALA (3,12; 1,80E-35), RARRES1 (3,27; 4,90E-07), RARRES3 (7,16; 2,20E-38), RBM47 (3,21; 5,88E-13), RBMS1 (3,17; 5,00E-39), RDH8 (3,07; 1,11E-03), RELB (9,41; 1,50E-44), RELN (3,68; 6,10E-05), RFX8 (3,22; 3,30E-05), RGS20 (2,88; 2,42E-13), RHBDF2 (4,05; 4,80E-17), RIPK2 (9,93; 2,00E-140), RNF19B (6,89; 7,64E-49), ROBO4 (2,99; 3,00E-06), ROS1 (4,97; 1,90E-08), RP11-1055B8,7 (13,16; 2,60E-32), RP11-352D3,2 (11,08; 1,20E-11), RP11-366L20,2 (4,73; 1,10E-08), RP11-468E2,4 (3,03; 3,00E-06), RRAD (3; 3,78E-04), RTP4 (5,08; 3,90E-08), S100A3 (4,12; 3,70E-11), S1PR3 (6,7; 9,10E-41), SAMD4A (3,55; 5,00E-21), SAMD9 (3,44; 7,89E-19), SAMD9L (10,01; 6,60E-49), SBNO2 (3,21; 6,20E-32), SCARF1 (3,03; 1,00E-05), SCG5 (2,86; 4,50E-04), SDC4 (3,77; 8,90E-26), SECTM1 (4; 1,69E-10), SELPLG (4,44; 6,00E-09), SEMA3C (3,24; 3,47E-28), SEMA4D (8,11; 3,30E-20), SEMA7A (2,99; 6,00E-06), SERPINA1 (5,98; 1,16E-25), SERPINB2 (26,65; 4,12E-37), SERPINB7 (3,31; 1,00E-06), SERPINB8 (5,91; 3,00E-39), SERPINE1 (2,92; 7,30E-05), SERPINE2 (5,75; 1,27E-22), SH2B3 (2,9; 1,83E-16), SHB (3,49; 5,80E-20), SHC4 (2,94; 2,44E-04), SLA (3,56; 2,00E-05), SLAMF8 (6,98; 3,00E-15), SLC15A3 (5,98; 4,70E-50), SLC22A23 (3,75; 1,31E-28), SLC25A22 (3,14; 7,00E-15), SLC25A37 (4,97; 1,24E-31), SLC28A3 (3,23; 6,30E-05), SLC2A6 (9,19; 1,39E-43), SLC35E4 (3,17; 9,60E-11), SLC39A14 (6,27; 3,50E-92), SLC41A2 (3,49; 2,00E-27), SLC7A2 (3,06; 9,00E-15), SLIT2 (3,41; 8,00E-12), SMAD3 (3,18; 7,98E-61), SMAD7 (2,96; 1,91E-07), SMOX (3,94; 3,85E-16), SMTNL1 (5,63; 7,60E-11), SNAI2 (3,07; 1,50E-11), SOCS1 (10,64; 2,00E-27), SOCS3 (5,58; 2,00E-39), SOD2 (19,71; 1,81E-43), SP100 (3,52; 1,69E-58), SP110 (4,34; 5,60E-44), SPAG17 (3,01; 2,70E-05), SPHK1 (6,79; 8,09E-16), SPOCD1 (2,97; 1,00E-06), SPRY4 (4,51; 1,00E-15), SPSB1 (4,3; 7,00E-33), SQSTM1 (2,89; 5,00E-12), SRGN (3,34; 2,00E-30), SSTR2 (6,49; 6,37E-13), ST3GAL4 (3,4; 6,00E-24), STARD8 (3,73; 1,82E-19), STAT1 (6,22; 2,60E-131), STAT5A (3,42; 6,70E-14), STC1 (5,43; 1,42E-13), SVIL (3,06; 6,51E-34), TAF4B (3,09; 5,30E-14), TAP1 (20,35; 1,00E-129), TAP2 (6,6; 7,00E-72), TAPBP (3,07; 5,11E-46), TBX2 (5,25; 3,00E-21), TBX21 (3,97; 1,00E-06), TBX3 (3,74; 9,40E-17), TEX29 (3,69; 1,00E-05), TFPI2 (3,23; 2,00E-06), TGM2 (5,4; 6,00E-12), TIFA (3,49; 2,27E-34), TIMP3 (3,34; 2,00E-36), TJP2 (3,32; 9,00E-54), TLR2 (2,96; 4,54E-04), TLR4 (4,38; 4,00E-27), TM4SF1 (3,52; 2,00E-17), TMEM132A (3,31; 1,39E-13), TMEM158 (10,29; 2,00E-18), TMEM171 (3,42; 6,63E-22), TMEM229B (3,68; 2,00E-06), TMEM239 (5,55; 7,00E-09), TMEM51 (5,07; 9,30E-47), TMEM52B (5,63; 6,39E-16), TNC (7,32; 1,50E-17), TNF (5,03; 1,00E-06), TNFAIP2 (71,59; 5,00E-138), TNFAIP3 (34,64; 6,10E-47), TNFAIP8 (4,74; 4,57E-40), TNFRSF11B (3,3; 3,40E-11), TNFRSF14 (3,85; 5,00E-09), TNFRSF1B (3,85; 1,00E-26), TNFRSF8 (3,34; 5,34E-07), TNFRSF9 (8,43; 2,20E-23), TNFSF10 (4,26; 1,30E-08), TNFSF13B (3,67; 1,30E-05), TNFSF14 (2,84; 2,09E-03), TNFSF4 (4,37; 4,00E-15), TNIP1 (9,41; 1,00E-66), TNIP3 (8,9; 4,15E-13), TRAF1 (23,48; 1,00E-36), TRIB1 (4,97; 1,20E-29), TRIB2 (3,59; 9,90E-14), TRIM16 (3,52; 4,00E-18), TRIM21 (4,37; 6,50E-35), TRIM25 (7,43; 3,30E-83), TRIM47 (5,38; 1,70E-29), TRIM69 (3,02; 4,60E-23), TRPA1 (3,87; 9,00E-09), TSKU (3,11; 5,00E-18), TYMP (4,34; 7,00E-15), UBD (5,16; 1,40E-08), UBE2L6 (3,94; 3,00E-54), UCN2 (3,95; 3,09E-07), USP18 (4,65; 1,70E-08), USP43 (4,08; 2,10E-14), VCAM1 (11,29; 2,32E-28), VEGFC (6,1; 1,58E-58), VEPH1 (2,95; 1,20E-08), VNN1 (4,45; 2,92E-07), WARS (4,24; 4,90E-26), WT1 (4,74; 7,00E-15), WTAP (3,5; 3,60E-47), XAF1 (6,68; 4,00E-42), XIRP1 (27,5; 1,40E-32), ZC3H12A (5,3; 2,01E-13), ZCCHC5 (3,06; 1,00E-05), ZNF267 (4,84; 1,90E-38), ZNF469 (7,59; 1,00E-17), ZNF697 (4,31; 1,00E-30), ZNFX1 (3,7; 2,00E-45), ZSWIM4 (3,49; 1,40E-20) |
|  | lincRNA | **Down:** AC006273,5 (-2,95; 2,60E-05), AC007405,6 (-3,05; 1,63E-04), AC096772,6 (-2,86; 1,00E-12), CTC-459F4,1 (-2,85; 1,63E-04), LINC00669 (-3,01; 1,20E-11), RP11-160O5,1 (-3,95; 7,00E-09), RP11-276H19,1 (-3,61; 2,70E-05), RP11-284F21,9 (-3,26; 1,30E-05), RP11-495P10,5 (-4,4; 1,30E-05), RP11-495P10,8 (-6,14; 1,80E-08), RP11-61A14,3 (-4,06; 9,00E-24), RP11-690G19,3 (-3,73; 3,66E-16), RP11-91J19,4 (-3,81; 2,87E-16), RP1-193H18,2 (-5,73; 9,67E-10), RPS10P7 (-3,29; 6,60E-05)  **Up:** AC019117,2 (3,23; 4,13E-04), CITF22-49D8,1 (5,92; 1,00E-06), CTC-296K1,4 (3,56; 3,00E-05), CTD-2023M8,1 (3,55; 3,06E-04), LINC00152 (2,99; 7,10E-19), LINC00511 (3,14; 6,00E-12), LINC00702 (4,54; 4,00E-11), LINC00842 (6,01; 5,13E-22), LINC00862 (2,93; 1,66E-04), LINC00973 (3,44; 1,50E-05), LINC01010 (2,94; 2,84E-04), MIR146A (3,61; 4,20E-08), MIR155HG (12,1; 1,20E-35), MIR29A (3,76; 1,10E-11), RP11-1002K11,1 (4,87; 3,16E-19), RP11-150O12,1 (3,34; 2,28E-07), RP11-154H12,2 (8,15; 1,30E-11), RP11-154H23,3 (3,39; 7,55E-07), RP11-22L13,1 (2,96; 2,33E-04), RP11-230G5,2 (3,31; 2,51E-07), RP11-245M24,1 (3,05; 1,09E-04), RP11-338C15,3 (3,11; 4,97E-04), RP11-359E10,1 (5,35; 7,00E-15), RP11-437J19,1 (2,84; 9,00E-06), RP11-495P10,1 (3,44; 1,50E-05), RP11-4C20,3 (3,36; 6,60E-05), RP11-680H20,2 (3,18; 2,20E-04), RP11-79H23,3 (21,82; 1,10E-17), RP11-861A13,4 (7,63; 2,08E-10), RP11-863P13,3 (3,62; 1,98E-04), RP11-91K9,1 (10,78; 1,03E-13), RP3-467K16,4 (3,36; 7,10E-05), RP4-555D20,2 (4,88; 1,60E-11), RP4-564F22,5 (2,95; 1,58E-04), RP4-794H19,4 (3,08; 1,30E-05), RP6-99M1,2 (2,88; 3,88E-07), TAPSAR1 (3,84; 3,70E-20), TEX41 (3,33; 2,50E-05) |
|  | miRNA | **Up:** AC019117,2 (3,23; 4,13E-04) |
|  | SnoRNA | **Up:** SNORA51 (3,67; 1,40E-05), SNORD14E (3,29; 2,60E-05), SNORD93 (3,38; 3,00E-06) |
|  | snRNA | **Up:** RNU7-124P (2,83; 6,67E-04) |
|  | pseudogene | **Down:** AC079781,5 (-4,54; 1,00E-06), ADAM1B (-3,22; 1,60E-05), ASNSP1 (-3,81; 3,80E-05), ECEL1P2 (-3,32; 4,63E-04), LRRC37A6P (-3,98; 1,60E-08), MORF4L1P4 (-3,24; 4,60E-04), RP11-396K3,1 (-2,99; 6,00E-06), RP11-495P10,9 (-5,06; 4,30E-08), RP11-694I15,7 (-2,97; 5,10E-05), SLC9A7P1 (-3,88; 1,00E-06)  **Up:** AC006028,11 (3,28; 4,90E-05), AC007362,1 (10,05; 3,00E-51), AC067945,3 (2,9; 4,45E-04), CMAHP (5,5; 2,97E-19), GBP1P1 (28,68; 1,20E-38), HIN1L (2,97; 7,20E-08), HNRNPA1P33 (3,97; 4,00E-06), IMPDH1P10 (3,23; 1,30E-04), KRT121P (3,58; 1,70E-05), LGALS17A (3,11; 1,00E-05), MT2P1 (5,26; 1,50E-08), MTCYBP3 (3,43; 3,10E-05), MTND1P11 (2,88; 4,77E-04), PTGES3P1 (14,72; 8,74E-31), RP11-322D14,1 (2,92; 3,29E-04), RP11-383F6,1 (3,15; 3,40E-05), RP11-486B10,4 (3,3; 3,10E-05), RP11-638I2,10 (3,62; 2,00E-06), RP11-670E13,3 (2,95; 5,94E-04), RP11-680H20,1 (4,1; 1,50E-05), RP11-730A19,9 (3,15; 1,00E-06), RP11-737O24,3 (2,95; 3,39E-04), RP11-75L1,2 (3,5; 8,00E-06), RP3-437C15,1 (2,88; 2,90E-04), RP4-663N10,2 (3,04; 4,93E-04), RPL12P18 (4,27; 2,00E-06), RPSAP52 (4; 8,90E-07), SVILP1 (3; 1,80E-05) |
|  | antisense | **Down:** HOTAIR (-3,04; 1,67E-04), PLCE1-AS1 (-3,97; 5,00E-05), RP11-125B21,2 (-3,16; 6,77E-04), RP11-390P2,4 (-3,8; 3,00E-06), RP1-39G22,7 (-3,21; 9,50E-11), RP1-93I3,1 (-2,93; 8,00E-06), RP3-508I15,9 (-3,38; 1,20E-11), RP5-1157M23,2 (-3,62; 1,20E-05), TAPT1-AS1 (-3,76; 9,60E-14), UGDH-AS1 (-3,21; 2,90E-07), ZBED3-AS1 (-4,15; 4,30E-08)  **Up:** AC002454,1 (2,9; 1,35E-03), AC004837,5 (2,96; 7,56E-04), AC006159,5 (3,03; 3,40E-05), AC006160,5 (8,43; 4,56E-16), AC007099,1 (3,01; 4,53E-04), AC007362,3 (8,02; 3,00E-15), AC007750,5 (3,65; 1,87E-07), AC009133,12 (2,91; 1,80E-04), AC009950,2 (3,34; 5,80E-11), AC020571,3 (4,7; 5,60E-08), AC116366,6 (3,56; 5,00E-09), C1RL-AS1 (3,26; 2,90E-20), CTD-2031P19,3 (7,75; 1,98E-16), CTD-2240E14,4 (3,88; 7,00E-06), CTD-2260A17,1 (3,38; 1,01E-10), CTD-2260A17,3 (2,96; 2,01E-04), CTD-2552K11,2 (2,84; 1,03E-04), INHBA-AS1 (5,21; 1,85E-07), LINC00525 (4,27; 1,32E-07), LINC01137 (3,06; 2,20E-05), RP11-134L10,1 (3,01; 1,01E-04), RP11-142M10,2 (3,41; 4,00E-06), RP11-165P7,1 (3,53; 7,00E-06), RP11-24J23,2 (2,85; 3,30E-03), RP11-313E19,2 (4,92; 9,00E-09), RP11-327F22,2 (4,94; 1,18E-07), RP11-356I2,4 (10,39; 1,60E-17), RP11-404P21,3 (3,51; 4,60E-05), RP11-443A13,5 (3,71; 6,00E-06), RP11-44K6,4 (3,44; 8,51E-07), RP11-473M20,7 (3,57; 2,00E-21), RP11-519G16,3 (3,12; 6,24E-04), RP11-58K22,4 (4,08; 2,00E-06), RP11-630D6,5 (3,97; 5,90E-05), RP11-638I2,9 (3,65; 7,00E-06), RP11-875O11,1 (4,1; 1,01E-07), RP11-981P6,1 (2,91; 8,90E-05), RP1-50J22,4 (3,92; 3,00E-06), RP1-68D18,4 (4,3; 7,00E-09), RP3-416H24,1 (3,43; 3,00E-05), RP4-779E11,3 (4,16; 2,20E-05), RP5-1142A6,2 (3,49; 3,00E-09), RP5-1142A6,8 (3,2; 1,60E-05), STARD4-AS1 (3,17; 1,40E-08) |
|  | processed_transcript | **Down:** LINC00263 (-3,54; 9,00E-09), LINC00899 (-3,71; 8,00E-06), RP11-356J5,12 (-3,85; 3,50E-08)  **Up:** APCDD1L-AS1 (3,54; 1,40E-05), PITRM1-AS1 (2,85; 3,00E-06) |
|  | sense_overlapping | **Down:** RP11-119F7,5 (-2,85; 3,50E-08), RP11-462G12,2 (-3,25; 4,00E-06) |
|  | sense_intronic | **Up:** RP11-22H5,2 (4,07; 2,00E-06), RP11-404P21,5 (3,64; 2,70E-05), RP1-68D18,2 (3,32; 5,20E-05) |
|  | Mt-RNA | **Up:** MT-RNR2 (3,57; 1,30E-08) |
|  | misc_RNA | **Up:** RN7SL834P (3,2; 9,60E-05) |
|  | rRNA | **Up:** RNA18S5 (3,33; 2,20E-08) |
|  |  |  |
| **T=12h**  **847 DEGs** | protein_coding | **Down:** SEPT3 (-3,65; 2,99E-07), AARS (-3,14; 2,59E-12), ABCA3 (-3,54; 1,80E-06), ABCA7 (-2,98; 2,39E-07), ABCA8 (-3,04; 1,40E-04), AC117395,1 (-2,93; 8,58E-07), ACADL (-4,69; 7,18E-09), ACBD4 (-2,90; 1,57E-06), ACBD7 (-3,46; 1,38E-10), ADAM22 (-3,81; 3,54E-24), ADAM33 (-3,85; 1,25E-14), ADAMTS13 (-2,86; 2,88E-04), ADAMTS15 (-3,32; 1,06E-08), ADM2 (-6,17; 5,39E-11), ADORA1 (-2,86; 4,99E-06), AIF1L (-3,40; 2,52E-08), AIG1 (-3,27; 1,28E-24), AKAP6 (-3,91; 9,57E-07), AKR1C2 (-3,67; 1,01E-05), AKR1C3 (-3,44; 3,36E-08), ALDH1L2 (-4,19; 1,04E-08), ALDH3B1 (-3,13; 1,57E-11), ALDH4A1 (-3,01; 4,23E-10), ALDH5A1 (-3,08; 9,53E-12), AMIGO1 (-3,42; 5,22E-08), ANK2 (-3,50; 2,35E-08), ANXA9 (-3,86; 2,15E-06), ARHGAP9 (-2,97; 6,51E-05), ARMC4 (-3,72; 8,87E-10), ASIC1 (-3,41; 5,60E-13), ASNS (-3,84; 2,50E-06), ATP6V0E2 (-2,84; 4,68E-08), ATP6V1G2 (-3,09; 3,67E-05), BMP4 (-3,08; 2,75E-10), C10orf10 (-3,24; 9,50E-06), C11orf87 (-7,32; 2,78E-14), C17orf103 (-2,93; 3,34E-10), C1orf115 (-3,19; 1,53E-12), C1orf228 (-3,61; 1,22E-05), C1QL4 (-3,55; 8,82E-11), C5 (-3,97; 3,43E-14), C9orf9 (-3,33; 5,97E-11), CACNG4 (-3,12; 1,37E-07), CBS (-5,47; 5,21E-11), CD200 (-3,50; 8,02E-08), CD248 (-3,81; 5,48E-18), CECR1 (-2,87; 2,18E-04), CELSR2 (-4,36; 1,03E-24), CHAC1 (-5,44; 6,75E-09), CHRM4 (-3,03; 2,04E-05), CLMN (-3,27; 1,25E-16), CMTM4 (-3,47; 1,42E-56), CNTN5 (-2,90; 2,92E-04), COL11A1 (-3,00; 3,06E-08), COL4A4 (-2,85; 2,51E-06), CORO6 (-2,93; 2,73E-06), CRYAB (-3,44; 1,60E-04), CSRNP3 (-3,39; 2,85E-10), CTF1 (-2,96; 1,53E-10), CTH (-3,20; 2,74E-06), CXADR (-4,40; 6,70E-16), CYS1 (-5,08; 1,28E-11), DAAM2 (-3,43; 9,44E-13), DBP (-6,41; 1,50E-25), DENND2A (-3,86; 9,00E-24), DHRS3 (-4,35; 4,55E-08), DIP2C (-3,11; 9,32E-26), DMGDH (-4,94; 1,00E-10), DUSP1 (-4,35; 6,77E-13), ECM2 (-5,14; 3,54E-10), EDA2R (-2,98; 4,09E-11), EFHD1 (-3,48; 2,81E-07), EGR1 (-6,25; 4,46E-16), EGR3 (-2,94; 5,86E-07), EPS8L2 (-3,20; 5,29E-12), ERBB3 (-5,18; 3,01E-16), EXPH5 (-3,17; 7,51E-12), F2RL2 (-2,99; 3,61E-18), FAM124A (-3,64; 3,61E-07), FAM13C (-2,84; 4,16E-04), FAM172A (-3,27; 4,26E-27), FAM198B (-3,81; 2,82E-19), FAM213A (-3,32; 3,03E-05), FAM229B (-2,97; 1,16E-10), FAM57B (-2,97; 1,37E-04), FAT3 (-3,69; 3,31E-15), FAXDC2 (-4,04; 2,08E-12), FHL1 (-2,94; 3,95E-10), FKBP1B (-3,07; 2,12E-07), FLRT1 (-3,49; 6,06E-05), FOS (-18,45; 1,58E-28), FOSB (-8,85; 6,54E-22), FRY (-3,20; 1,24E-14), GDF15 (-6,52; 3,25E-13), GDPD5 (-2,91; 7,76E-09), GFRA1 (-2,85; 2,18E-06), GLRB (-4,28; 4,60E-19), GLT8D2 (-2,83; 1,15E-06), GPR155 (-2,84; 1,25E-11), GPT2 (-3,04; 2,03E-08), GRAMD2 (-3,06; 7,23E-04), GSTA4 (-3,00; 1,41E-10), GUCY1A2 (-3,12; 1,02E-07), GYG2 (-4,66; 7,99E-16), HES6 (-2,84; 4,85E-04), HHLA3 (-2,90; 3,70E-08), HOXB9 (-2,92; 3,18E-12), HPSE (-3,16; 1,04E-14), ID1 (-4,42; 5,56E-12), IL20RB (-5,71; 2,38E-11), IL21R (-2,95; 1,24E-04), IMPA2 (-2,95; 4,03E-10), INADL (-3,77; 2,90E-14), INHBE (-9,61; 1,26E-16), INPP5D (-3,45; 6,78E-06), INPP5J (-3,26; 3,05E-08), ITGB4 (-3,42; 4,85E-06), JDP2 (-3,16; 1,37E-14), KAL1 (-3,06; 1,75E-14), KCNAB2 (-2,87; 1,22E-09), KCNH3 (-3,34; 8,34E-08), KCNH7 (-6,91; 5,82E-13), KCTD16 (-3,75; 2,29E-06), KCTD19 (-3,63; 1,65E-06), KIAA1161 (-3,40; 4,20E-21), KIAA1211 (-3,03; 1,71E-06), KIAA1683 (-3,43; 6,14E-08), KIT (-6,18; 4,48E-17), KLF2 (-6,13; 4,59E-23), KLHL24 (-3,37; 2,22E-13), KLHL4 (-2,89; 2,50E-10), LMOD1 (-5,16; 6,67E-14), LOH12CR2 (-2,93; 6,30E-04), LRRC20 (-3,67; 6,22E-19), LRRC7 (-3,27; 3,95E-10), LRRN4CL (-3,16; 5,41E-06), LYPD6B (-3,21; 2,58E-07), LZTS3 (-3,03; 6,09E-14), MAMSTR (-3,09; 4,06E-12), MAN1C1 (-4,82; 1,19E-11), MANSC1 (-3,52; 1,28E-10), MAP2K6 (-5,37; 5,84E-11), MAPK10 (-3,13; 7,80E-06), MCAM (-3,00; 4,78E-25), MDH1B (-2,95; 3,49E-04), METTL7A (-4,68; 1,56E-10), METTL7B (-3,45; 3,95E-06), MGARP (-3,65; 1,10E-05), MID1IP1 (-3,09; 3,35E-14), MKX (-3,66; 3,13E-10), MMP11 (-3,23; 1,71E-05), MOAP1 (-2,92; 1,27E-11), MORN4 (-4,26; 5,42E-20), MOV10L1 (-2,83; 5,16E-04), MPP7 (-3,68; 5,05E-18), MRVI1 (-2,87; 1,76E-04), MXD3 (-3,57; 4,33E-15), MYEF2 (-2,91; 7,94E-08), MYO16 (-3,59; 3,04E-06), MYO18A (-3,35; 1,55E-17), NBEAL2 (-3,33; 1,27E-21), NCAM1 (-3,16; 1,72E-20), NDNF (-3,25; 2,34E-06), NDUFA4L2 (-3,09; 6,18E-05), NFE2 (-3,87; 5,92E-07), NFIA (-3,14; 2,32E-19), NICN1 (-2,87; 3,73E-07), NLGN1 (-3,06; 2,42E-11), NMBR (-3,01; 6,62E-04), NR4A1 (-8,90; 1,12E-39), NR4A2 (-3,02; 3,36E-06), NRBP2 (-3,55; 2,30E-15), NRCAM (-4,51; 2,00E-07), NT5M (-3,09; 2,97E-07), NUPR1 (-4,34; 1,35E-07), OBSL1 (-2,94; 5,68E-16), OCLN (-4,25; 2,15E-21), OPRL1 (-2,89; 5,01E-04), OSR2 (-3,56; 1,30E-10), P2RX6 (-3,41; 9,72E-10), PAPPA2 (-3,37; 3,15E-06), PARD3B (-4,13; 2,51E-16), PARD6A (-3,57; 1,02E-05), PCK2 (-3,84; 8,67E-07), PCSK9 (-3,71; 2,62E-08), PDE1A (-6,56; 2,85E-11), PDE1C (-3,85; 8,85E-15), PDZD7 (-5,84; 1,53E-11), PER3 (-2,83; 1,39E-14), PGPEP1 (-3,39; 1,50E-18), PHF7 (-3,29; 7,07E-13), PHOSPHO2 (-2,83; 1,08E-05), PIANP (-3,03; 2,12E-14), PIGZ (-2,84; 2,57E-05), PIK3C2B (-4,08; 5,05E-19), PKP2 (-5,76; 9,18E-11), PLAC8 (-6,13; 3,44E-17), PLEKHA6 (-4,87; 7,91E-12), PLXNB3 (-3,00; 2,66E-09), PPARGC1A (-5,59; 7,27E-11), PPFIA4 (-3,08; 3,20E-06), PPM1E (-5,16; 1,12E-13), PPP1R9A (-3,03; 6,47E-05), PRDM6 (-3,04; 4,97E-08), PRELP (-2,88; 3,42E-04), PRICKLE1 (-5,35; 1,81E-26), PRKAA2 (-2,88; 1,41E-10), PRRT4 (-3,24; 9,65E-05), PSAT1 (-5,95; 1,43E-14), PTK2B (-4,01; 4,26E-12), PTPN22 (-3,98; 8,02E-11), PTPRQ (-3,68; 4,27E-07), QPRT (-3,70; 1,18E-09), RAB11FIP1 (-3,54; 5,61E-20), RAB33A (-3,27; 7,36E-05), RALGPS1 (-2,87; 2,17E-09), RASSF2 (-3,29; 1,42E-05), RCAN2 (-3,30; 1,07E-10), REEP1 (-3,43; 5,74E-12), RGS22 (-2,91; 2,44E-04), RGS9 (-3,78; 7,23E-07), RHOB (-3,04; 6,21E-15), RIMS3 (-6,63; 5,98E-15), RNF157 (-3,14; 1,94E-13), RNFT2 (-3,97; 1,22E-26), RP11-553A10,1 (-3,38; 1,28E-05), RPS6KA2 (-2,88; 9,67E-14), RTN4R (-3,00; 2,57E-03), SCN2A (-2,86; 2,43E-06), SDPR (-5,09; 2,31E-11), SEL1L3 (-4,30; 5,87E-13), SEMA4G (-3,60; 1,82E-10), SEMA6B (-3,65; 1,97E-08), SESN3 (-3,12; 5,57E-14), SGCG (-3,19; 1,73E-04), SH3BGR (-3,08; 1,87E-04), SLC16A10 (-2,87; 1,37E-08), SLC16A14 (-7,94; 2,43E-26), SLC16A5 (-3,00; 2,90E-14), SLC1A4 (-3,66; 8,05E-09), SLC22A15 (-4,57; 1,67E-07), SLC29A2 (-3,27; 6,11E-08), SLC38A4 (-4,32; 7,05E-17), SLC40A1 (-3,66; 2,27E-13), SLC48A1 (-3,02; 1,27E-07), SLC7A11 (-6,75; 2,48E-14), SLC7A4 (-4,41; 4,51E-08), SLCO4C1 (-4,46; 1,48E-07), SLITRK3 (-4,70; 1,37E-06), SLITRK5 (-5,27; 3,24E-20), SMAD9 (-3,65; 1,53E-19), SORL1 (-3,08; 1,74E-12), SPEG (-3,05; 1,80E-31), SPRY1 (-2,92; 1,37E-04), SSBP2 (-4,47; 2,93E-20), STXBP6 (-4,42; 2,48E-15), SYBU (-4,10; 9,32E-08), SYPL2 (-3,00; 6,77E-16), SYTL5 (-2,84; 6,92E-05), TCEAL7 (-3,29; 5,68E-14), TENM2 (-3,50; 1,03E-08), TET1 (-3,16; 2,28E-18), THRB (-3,23; 1,27E-14), TLL1 (-4,83; 2,05E-08), TMEM170B (-5,26; 3,45E-29), TMEM178B (-3,08; 9,35E-05), TMEM187 (-3,09; 5,31E-07), TMEM56 (-4,27; 1,28E-26), TNFRSF10D (-3,05; 4,38E-17), TOX2 (-3,14; 9,84E-16), TP53INP1 (-2,90; 5,93E-13), TPD52L1 (-3,69; 5,82E-06), TRIB3 (-6,12; 2,15E-13), TTLL1 (-3,15; 1,28E-08), TUB (-3,39; 9,44E-22), UCP2 (-2,93; 1,83E-06), ULBP1 (-4,20; 7,28E-15), VLDLR (-4,13; 3,98E-14), VWA5A (-3,03; 8,25E-05), WDR31 (-3,77; 5,26E-21), YPEL1 (-4,79; 2,37E-09), ZBED3 (-3,12; 3,12E-14), ZC3H6 (-3,15; 1,85E-08), ZMAT3 (-4,02; 6,69E-31), ZMYND12 (-3,23; 6,00E-06), ZNF185 (-3,32; 2,50E-08), ZNF385D (-3,53; 7,98E-12), ZNF395 (-3,46; 6,30E-10), ZNF516 (-2,85; 1,07E-11)  **Up:** AC090673,2 (3,27; 7,12E-05), ACAN (8,24; 3,77E-10), ACO1 (3,32; 2,23E-66), ACSL5 (9,04; 1,94E-20), ADAP1 (6,54; 1,68E-11), AIM2 (16,73; 4,69E-14), AKR1B1 (3,86; 1,34E-16), AMPD3 (5,43; 4,82E-14), ANGPTL4 (3,90; 2,41E-06), ANKRD55 (3,00; 8,97E-05), ANO4 (9,08; 5,62E-47), ANTXR2 (3,37; 3,77E-13), ANXA8 (5,67; 2,13E-11), ANXA8L1 (3,83; 9,18E-06), ANXA8L2 (6,22; 1,00E-14), APCDD1L (3,70; 3,27E-08), APOL1 (7,90; 6,04E-57), APOL2 (5,38; 4,63E-36), APOL3 (14,44; 5,14E-76), APOL4 (55,81; 3,67E-78), APOL6 (9,23; 1,09E-92), ARHGAP42 (3,61; 6,54E-22), ARID5A (4,13; 9,23E-21), ART3 (4,05; 5,73E-05), BATF2 (31,98; 7,20E-50), BATF3 (3,77; 1,15E-30), BCL3 (3,01; 2,83E-41), BDKRB1 (10,60; 3,08E-26), BDKRB2 (3,88; 1,72E-09), BEST3 (3,87; 8,18E-07), BID (4,69; 6,25E-33), BIRC3 (10,84; 1,68E-23), BMPER (3,01; 1,19E-05), BMPR1B (2,85; 4,23E-05), BST2 (8,72; 4,21E-27), BTN3A3 (2,90; 6,22E-16), C1orf147 (3,46; 8,30E-06), C1orf204 (2,90; 1,75E-03), C1QTNF1 (17,47; 2,96E-33), C1R (3,03; 1,18E-14), C1S (5,99; 7,41E-40), C20orf141 (6,09; 3,84E-10), C3 (6,94; 2,27E-42), C5orf56 (3,29; 2,38E-11), C9orf47 (4,02; 3,62E-07), CADM1 (3,00; 3,63E-09), CAMK1G (4,75; 2,53E-07), CCDC3 (6,41; 4,44E-38), CCL2 (33,06; 3,35E-61), CCL5 (6,10; 5,08E-09), CCL7 (5,38; 1,10E-08), CCL8 (3,55; 6,13E-05), CCND1 (4,75; 1,25E-08), CD274 (7,96; 8,02E-20), CD44 (4,13; 1,62E-13), CD47 (3,44; 5,52E-42), CD7 (4,21; 5,21E-07), CD70 (3,53; 1,33E-07), CD74 (26,41; 1,09E-35), CD82 (4,68; 7,03E-11), CD83 (5,52; 2,45E-19), CDCP1 (5,92; 9,47E-16), CETP (3,19; 2,55E-05), CFB (3,83; 2,94E-10), CGB7 (2,85; 1,26E-05), CHRNA1 (4,50; 3,63E-08), CHRNB2 (3,06; 1,24E-04), CHST11 (3,72; 4,30E-09), CHST15 (5,16; 2,83E-19), CHSY3 (2,96; 3,71E-08), CIITA (46,12; 5,36E-58), CLDN1 (3,09; 4,48E-06), CLIC2 (10,92; 6,42E-35), CMPK2 (11,42; 1,17E-15), COL17A1 (4,33; 6,88E-08), COL27A1 (6,70; 4,31E-21), COL7A1 (4,14; 1,03E-09), CR1L (2,94; 6,90E-04), CSF1 (6,29; 1,96E-75), CTC-479C5,12 (5,90; 1,42E-16), CTD-2547L24,3 (3,64; 2,82E-05), CTSS (43,71; 3,21E-66), CUBN (3,12; 1,66E-08), CX3CL1 (3,87; 1,27E-04), CXCL1 (10,27; 5,74E-20), CXCL10 (48,10; 2,59E-42), CXCL11 (38,65; 2,22E-35), CXCL12 (2,97; 1,23E-08), CXCL2 (3,77; 6,69E-15), CXCL3 (4,04; 1,93E-09), CXCL5 (6,46; 6,63E-13), CXCL9 (17,86; 5,91E-29), DACT1 (3,23; 6,67E-10), DDX58 (8,31; 8,96E-64), DDX60 (4,32; 9,11E-54), DDX60L (3,13; 1,19E-29), DEC1 (4,77; 5,12E-08), DGKG (3,69; 7,18E-05), DGKI (4,11; 3,35E-07), DLL1 (3,50; 5,87E-05), DNER (3,11; 6,07E-07), DNPEP (3,19; 4,29E-44), DOCK10 (3,70; 1,78E-35), DPYSL3 (3,16; 1,48E-30), DRAM1 (5,42; 1,60E-37), DTX3L (5,53; 5,11E-85), EBI3 (3,27; 3,59E-11), EFHD2 (3,16; 3,78E-24), EPSTI1 (9,27; 5,57E-57), ERAP1 (3,65; 9,46E-66), ERAP2 (3,59; 2,65E-36), ESM1 (3,17; 2,42E-04), ETS2 (2,99; 3,48E-27), ETV7 (13,84; 2,62E-47), FAM101A (5,12; 1,06E-14), FAM115C (4,31; 2,07E-11), FAM177B (4,02; 4,32E-05), FAM180A (3,42; 7,04E-06), FAM19A3 (4,71; 7,85E-07), FAM26F (3,64; 4,05E-06), FBXO32 (3,75; 2,22E-13), FBXO6 (4,76; 7,27E-08), FCRL6 (4,58; 9,06E-07), FDCSP (5,85; 4,37E-10), FGF22 (2,99; 3,14E-04), FLT1 (3,21; 1,98E-04), FOSL1 (3,50; 1,34E-07), FOXC2 (3,21; 5,88E-10), FST (4,06; 2,66E-17), G0S2 (5,25; 1,75E-12), GBP1 (35,10; 4,59E-112), GBP2 (55,31; 7,96E-115), GBP3 (10,12; 2,29E-62), GBP4 (112,77; 1,27E-80), GBP5 (77,23; 4,23E-70), GCH1 (3,87; 2,26E-22), GFPT2 (5,35; 7,87E-13), GGT5 (3,77; 3,46E-06), GIMAP2 (3,40; 2,15E-05), GOLM1 (3,30; 4,47E-33), GPR115 (5,78; 4,64E-10), GPR158 (3,45; 6,75E-12), GPR68 (9,98; 4,48E-16), GPRC5B (5,25; 7,81E-22), GREM1 (3,84; 1,37E-11), GSAP (3,79; 5,39E-19), HAPLN3 (12,96; 1,82E-58), HAS2 (5,50; 1,92E-10), HDAC9 (3,90; 1,30E-30), HECW2 (4,51; 5,24E-29), HELZ2 (3,23; 1,01E-21), HERC6 (3,93; 1,38E-29), HIC1 (2,93; 1,20E-08), HLA-E (3,23; 1,84E-26), HLA-F (4,73; 5,47E-21), HMGA1 (3,25; 5,51E-08), HMGA2 (4,74; 1,89E-10), HORMAD1 (4,02; 2,02E-05), HS3ST3A1 (4,03; 2,43E-08), HS3ST3B1 (5,15; 2,67E-22), HSD11B1 (3,08; 1,01E-05), ICAM1 (11,64; 3,60E-33), ICOSLG (2,83; 7,82E-06), IDO1 (59,80; 4,50E-52), IFI27 (3,43; 6,01E-06), IFI30 (3,05; 9,07E-04), IFI35 (4,92; 6,43E-79), IFI44 (3,93; 2,88E-18), IFI44L (15,86; 1,36E-35), IFIH1 (4,93; 3,94E-31), IFIT2 (4,70; 1,50E-19), IFIT3 (15,31; 9,32E-91), IFIT5 (4,03; 3,57E-58), IKBKE (4,53; 9,51E-29), IL11 (3,35; 8,63E-05), IL12RB1 (3,62; 2,57E-05), IL15 (3,37; 3,71E-11), IL15RA (15,44; 2,62E-47), IL18BP (10,68; 3,64E-96), IL1B (10,71; 1,36E-18), IL31RA (2,94; 3,87E-22), IL32 (43,20; 5,18E-82), IL33 (4,70; 9,29E-05), IL34 (2,91; 4,30E-04), IL4I1 (3,85; 2,08E-05), IL4R (3,47; 1,35E-20), IL6 (7,14; 4,70E-14), IL7 (3,76; 1,42E-05), IL7R (6,12; 9,70E-23), IL8 (13,24; 4,83E-25), INHBA (12,26; 1,53E-22), IRAK2 (9,37; 4,17E-31), IRF1 (16,19; 1,36E-73), IRF2 (3,18; 1,77E-33), IRF9 (3,21; 2,18E-09), ISG20 (6,31; 6,45E-16), ITGA2 (4,29; 1,94E-08), ITK (24,26; 9,45E-18), JAK2 (3,37; 1,39E-28), KCNA7 (2,87; 1,13E-05), KCNJ15 (5,75; 1,10E-10), KCNQ5 (3,18; 1,37E-06), KDR (5,73; 1,89E-09), KIAA1199 (2,95; 1,68E-06), KIAA1644 (6,78; 5,54E-23), KLF17 (3,01; 1,99E-04), KYNU (3,31; 2,79E-05), LACTB (3,60; 2,00E-29), LAMB3 (9,82; 5,49E-28), LAMC2 (4,23; 4,96E-15), LAP3 (9,81; 7,28E-153), LGALS9 (4,45; 1,69E-07), LIMD2 (3,15; 2,92E-11), LIPG (5,80; 1,12E-07), LPXN (5,40; 4,41E-16), LRRC15 (3,17; 4,59E-05), LRRN3 (5,34; 1,10E-08), LRRTM2 (4,82; 1,37E-10), LYN (4,53; 6,52E-24), LYPD5 (3,63; 4,91E-08), MDGA1 (3,32; 4,21E-15), MEFV (7,04; 3,13E-09), MEOX1 (3,70; 1,17E-05), METTL1 (2,89; 1,55E-16), MGLL (4,02; 1,98E-18), MLKL (8,27; 3,51E-28), MMP1 (3,25; 1,15E-06), MRPS24 (3,77; 3,51E-08), MSC (8,62; 7,16E-38), MT1X (3,61; 7,09E-18), MT2A (6,06; 3,98E-18), MTSS1 (4,45; 6,73E-28), MX1 (11,22; 8,96E-31), MX2 (8,67; 1,03E-15), MYEOV (3,67; 1,47E-06), MYO10 (4,85; 1,67E-16), MYPN (4,85; 2,50E-10), NAMPT (3,17; 3,41E-33), NAV2 (3,70; 1,95E-12), NEDD9 (3,69; 3,10E-24), NFATC2 (2,90; 2,68E-04), NFE2L3 (4,16; 1,65E-17), NFKB1 (4,35; 1,12E-59), NFKB2 (3,99; 3,78E-31), NFKBIA (7,42; 1,91E-49), NFKBIE (3,32; 2,73E-12), NKX3-1 (4,04; 3,92E-22), NLRC5 (5,46; 1,01E-113), NLRP3 (4,84; 2,35E-07), NMI (5,42; 1,28E-72), NPAS2 (2,83; 5,43E-08), NPTX1 (5,77; 6,10E-09), NREP (3,26; 2,88E-13), NRG1 (3,31; 7,57E-14), NRP1 (2,89; 8,21E-28), NRP2 (7,12; 2,45E-46), NT5E (2,91; 1,29E-07), NTM (4,35; 4,32E-07), NTN1 (3,96; 7,04E-08), NUAK2 (3,23; 1,26E-15), NUB1 (2,93; 8,48E-81), OAF (3,01; 1,77E-13), OAS1 (8,91; 2,57E-17), OAS2 (23,13; 1,90E-43), OAS3 (7,07; 6,35E-55), OASL (10,04; 3,31E-29), ODF3B (5,26; 1,92E-09), OSGIN1 (2,85; 2,47E-08), OTOGL (3,42; 2,46E-05), OTUD4 (2,83; 2,01E-37), PARP12 (4,06; 2,80E-58), PARP14 (10,45; 9,58E-84), PARP9 (3,24; 1,44E-26), PAX8 (4,55; 2,55E-11), PCDH7 (3,88; 8,34E-08), PDCD1LG2 (5,48; 4,48E-16), PDLIM4 (3,37; 2,22E-19), PFKFB4 (3,32; 4,11E-12), PHACTR1 (8,26; 1,37E-31), PHF11 (3,66; 8,72E-26), PHLDA1 (3,08; 4,31E-11), PID1 (3,08; 1,37E-21), PIK3CD (3,15; 6,53E-23), PLA2G4A (2,87; 3,43E-07), PLAUR (3,05; 2,32E-09), PLSCR1 (3,31; 8,56E-49), PMAIP1 (3,00; 4,58E-27), PML (4,32; 1,89E-69), PODXL (3,47; 4,11E-09), POU2F2 (4,40; 4,54E-13), PPA1 (2,92; 3,18E-19), PPAP2B (3,66; 7,66E-20), PPIF (4,10; 1,80E-20), PPP4R4 (3,03; 6,23E-04), PRDM1 (3,77; 1,35E-17), PRDM8 (3,13; 3,18E-21), PSMA2 (2,99; 7,67E-13), PSMA6 (3,23; 9,99E-11), PSMB10 (5,51; 9,87E-24), PSMB8 (3,63; 7,16E-25), PSMB9 (5,55; 5,26E-40), PSME2 (3,89; 9,12E-120), PTPRE (2,93; 6,36E-08), PTPRK (3,51; 2,16E-31), PTX3 (3,02; 9,62E-07), RAB27B (3,33; 5,07E-11), RALA (3,00; 5,36E-33), RARRES1 (2,85; 1,01E-05), RARRES3 (11,90; 2,99E-61), RELB (5,87; 9,01E-28), RHBDF2 (4,13; 1,74E-17), RIPK2 (7,00; 4,23E-101), RNF19B (5,08; 1,25E-34), ROBO4 (3,85; 3,01E-09), ROS1 (3,69; 4,77E-06), RP11-1055B8,7 (5,61; 6,50E-15), RP11-352D3,2 (12,29; 3,42E-12), RP11-366L20,2 (4,18; 1,43E-07), RP11-468E2,4 (3,22; 5,02E-07), RP11-766F14,2 (3,43; 7,95E-08), RSAD2 (3,74; 2,48E-05), RTP4 (6,00; 1,62E-09), S100A3 (3,38; 1,68E-08), S1PR3 (4,70; 5,92E-27), SAMD9 (3,35; 5,34E-18), SAMD9L (9,53; 9,21E-47), SECTM1 (4,46; 4,64E-12), SELPLG (4,45; 4,57E-09), SEMA3C (3,56; 1,09E-32), SEMA4D (13,33; 2,60E-30), SEMA7A (2,93; 7,90E-06), SERPINA1 (7,22; 2,52E-31), SERPINB2 (22,37; 5,77E-34), SERPINB7 (3,27; 1,64E-06), SERPINB8 (4,26; 4,36E-26), SERPINE1 (3,09; 2,46E-05), SERPINE2 (5,68; 3,19E-22), SLA (3,29; 5,24E-05), SLAMF8 (12,16; 5,46E-20), SLC15A3 (7,88; 5,09E-67), SLC1A3 (3,44; 3,61E-13), SLC25A22 (2,87; 1,03E-12), SLC25A37 (3,39; 1,69E-18), SLC28A3 (4,14; 1,15E-06), SLC2A6 (7,90; 9,38E-38), SLC39A14 (4,36; 3,54E-59), SLC6A6 (2,97; 4,66E-12), SLIT2 (2,94; 2,57E-09), SMAD7 (3,07; 5,96E-08), SMTNL1 (6,83; 4,67E-13), SOCS1 (9,27; 2,48E-24), SOCS3 (3,90; 1,16E-24), SOD2 (13,85; 6,26E-34), SP100 (4,09; 1,80E-73), SP110 (3,99; 4,61E-39), SPHK1 (3,84; 2,91E-08), SPRY4 (3,32; 4,15E-10), SPSB1 (4,00; 1,01E-29), SRGN (3,61; 3,50E-34), SSTR2 (6,75; 2,03E-13), STARD8 (3,02; 7,29E-14), STAT1 (8,86; 1,67E-187), STC1 (4,77; 1,09E-11), TAF4B (2,90; 1,51E-12), TAP1 (18,32; 1,68E-120), TAP2 (8,83; 7,82E-96), TAPBP (3,56; 4,77E-59), TAPBPL (2,99; 1,43E-17), TBX2 (4,88; 1,97E-19), TBX21 (4,57; 2,85E-07), TBX3 (3,38; 2,80E-14), TEX29 (4,25; 9,06E-07), TFPI2 (3,90; 2,90E-08), TGM2 (7,38; 2,14E-16), TLDC2 (3,64; 1,03E-09), TLR4 (4,10; 1,24E-24), TMEM132A (3,60; 1,91E-15), TMEM133 (3,58; 6,71E-19), TMEM158 (7,46; 2,90E-14), TMEM171 (3,11; 7,91E-19), TMEM229B (3,53; 3,23E-06), TMEM239 (3,83; 2,98E-06), TMEM51 (4,25; 3,84E-37), TMEM52B (3,12; 2,18E-07), TNC (9,74; 1,49E-22), TNFAIP2 (15,49; 2,38E-57), TNFAIP3 (18,28; 1,55E-32), TNFAIP6 (3,20; 3,43E-08), TNFAIP8 (2,96; 1,37E-19), TNFRSF14 (5,72; 1,13E-14), TNFRSF1B (3,45; 1,58E-22), TNFRSF8 (3,80; 1,90E-08), TNFRSF9 (5,16; 3,74E-14), TNFSF10 (7,09; 7,98E-15), TNFSF13B (7,63; 1,68E-11), TNIP1 (5,99; 2,07E-42), TNIP3 (6,60; 5,95E-11), TP63 (3,25; 3,23E-05), TRAF1 (17,85; 7,51E-31), TRIB1 (4,31; 1,39E-24), TRIB2 (3,42; 9,93E-13), TRIM21 (4,52; 1,46E-36), TRIM22 (4,17; 7,99E-16), TRIM25 (3,60; 7,66E-34), TRIM69 (2,94; 6,63E-22), TRPA1 (3,94; 5,31E-09), TYMP (4,29; 9,87E-15), UBD (12,32; 2,77E-12), UBE2L6 (5,38; 6,95E-82), UCN2 (5,24; 4,79E-10), UNC5C (2,94; 6,19E-04), USP18 (5,70; 1,46E-10), VCAM1 (7,31; 2,64E-19), VDR (2,88; 7,09E-15), VEGFC (6,18; 1,92E-59), VEPH1 (6,17; 2,59E-23), VIM (3,29; 2,66E-23), VNN1 (5,94; 1,89E-09), WARS (6,20; 2,89E-41), WT1 (3,26; 6,53E-09), XAF1 (6,39; 4,36E-40), XDH (2,91; 1,58E-04), XIRP1 (23,80; 1,61E-30), ZC3H12A (4,00; 1,61E-09), ZNF267 (3,47; 6,44E-24), ZNF469 (3,78; 3,63E-08), ZNF697 (3,62; 8,62E-24), ZNFX1 (3,16; 3,84E-35), ZSWIM4 (2,85; 1,61E-14) |
|  | lincRNA | **Down:** AC007405,6 (-2,96; 1,88E-04), AC009502,4 (-2,89; 3,11E-04), CITF22-92A6,1 (-4,05; 3,25E-07), PWAR6 (-3,13; 1,30E-04), RP11-160O5,1 (-3,74; 1,16E-08), RP11-276H19,1 (-3,43; 4,19E-05), RP11-284F21,9 (-4,11; 5,98E-08), RP11-495P10,5 (-4,75; 2,51E-06), RP11-495P10,8 (-4,26; 5,01E-06), RP11-499E18,1 (-2,85; 2,60E-04), RP11-508N22,12 (-3,45; 2,95E-05), RP11-723O4,9 (-2,94; 6,44E-05), RP11-91J19,4 (-3,99; 1,10E-17), RP1-193H18,2 (-5,05; 4,68E-09), RP5-902P8,10 (-3,13; 3,38E-04)  **Up:** AC003092,1 (2,97; 9,90E-05), AC019117,2 (3,11; 5,39E-04), AC131025,8 (3,01; 1,01E-06), AP001422,3 (3,42; 5,44E-06), CITF22-49D8,1 (3,96; 4,98E-05), CTD-2023M8,1 (3,76; 1,80E-04), LINC00312 (2,97; 1,50E-06), LINC00702 (3,28; 3,42E-07), LINC00842 (4,35; 6,08E-15), LINC00856 (2,86; 1,69E-03), LINC00941 (3,36; 5,73E-12), MIR146A (2,93; 5,78E-07), MIR155HG (8,26; 1,10E-25), RP11-1002K11,1 (3,46; 4,99E-12), RP11-154H12,2 (6,60; 2,07E-10), RP11-154H23,3 (3,96; 1,52E-08), RP11-359E10,1 (4,51; 3,86E-12), RP11-79H23,3 (11,53; 1,05E-13), RP11-861A13,4 (5,73; 4,02E-09), RP11-91K9,1 (6,75; 6,09E-11), RP4-555D20,2 (3,07; 3,07E-06), RP4-794H19,4 (3,44; 1,17E-06), TAPSAR1 (3,53; 6,96E-18) |
|  | miRNA | **Up:** MIR4482-1 (3,74; 3,73E-08) |
|  | SnoRNA | **Up:** SNORA51 (3,30; 5,03E-05), SNORD14E (2,98; 1,11E-04), SNORD6 (2,87; 6,90E-04), SNORD93 (3,88; 1,32E-07) |
|  | snRNA | **Down:** LINC00263 (-3,54; 009E-09), LINC00899 (-3,71; 008E-06), RP11-356J5,12 (-3,85; 035E-09)  **Up:** APCDD1L-AS1 (3,54; 014E-06), PITRM1-AS1 (2,85; 003E-06) |
|  | pseudogene | **Down:** AC079781,5 (-3,17; 6,39E-04), ASNSP1 (-4,78; 2,57E-07), CD24P4 (-2,91; 7,98E-04), ECEL1P2 (-4,47; 2,57E-06), LRRC37A16P (-3,03; 1,58E-25), LRRC37A6P (-2,99; 4,61E-06), MORF4L1P4 (-3,09; 4,11E-04), RP11-495P10,9 (-5,52; 2,94E-09), SLC9A7P1 (-4,99; 2,77E-09)  **Up:** AC007362,1 (8,36; 2,48E-43), CMAHP (8,93; 1,80E-31), GAPDHP14 (3,40; 1,27E-04), GBP1P1 (47,66; 2,64E-49), HSPA8P11 (3,88; 5,33E-05), KRT121P (3,81; 5,97E-06), LGALS17A (5,78; 1,42E-08), MT2P1 (4,64; 1,23E-07), MTND1P11 (3,25; 8,16E-05), MTND5P14 (3,15; 1,91E-05), OR2I1P (3,48; 2,00E-07), PTGES3P1 (17,60; 1,07E-34), RP11-383F6,1 (4,47; 3,41E-08), RP11-638I2,10 (4,97; 2,60E-09), RP11-680H20,1 (4,46; 5,40E-06), RPSAP52 (3,58; 5,69E-06) |
|  | antisense | **Down:** AC007246,3 (-3,11; 4,42E-09), CTD-2541M15,1 (-2,89; 6,65E-07), PLCE1-AS1 (-6,73; 1,56E-10), RP11-125B21,2 (-4,82; 2,07E-07), RP11-284F21,10 (-3,57; 1,03E-10), RP11-284F21,7 (-2,84; 2,13E-06), RP11-326C3,11 (-3,94; 1,03E-06), RP11-390P2,4 (-4,88; 5,49E-09), RP11-3L8,3 (-4,55; 3,99E-07), RP11-558F24,4 (-3,70; 1,30E-07), RP1-93I3,1 (-2,96; 4,00E-06), TAPT1-AS1 (-3,16; 7,33E-11), ZBED3-AS1 (-3,20; 5,87E-06)  **Up:** AC006160,5 (9,74; 4,90E-18), AC007362,3 (5,43; 1,15E-10), AC007750,5 (3,80; 5,12E-08), AC009133,12 (3,04; 7,52E-05), AC009950,2 (3,50; 5,62E-12), AC020571,3 (5,01; 1,58E-08), AC116366,6 (4,43; 3,22E-12), C1RL-AS1 (2,99; 1,78E-17), CTB-60B18,12 (3,04; 4,76E-05), CTD-2031P19,3 (5,54; 6,75E-12), CTD-2260A17,1 (3,12; 1,44E-09), INHBA-AS1 (2,95; 4,37E-04), LINC00525 (3,73; 1,70E-06), RP11-134L10,1 (3,37; 1,43E-05), RP11-165P7,1 (2,97; 1,06E-04), RP11-313E19,2 (4,52; 4,80E-08), RP11-327F22,2 (5,00; 9,04E-08), RP11-356I2,4 (6,92; 1,62E-12), RP11-404P21,3 (3,36; 7,24E-05), RP11-443A13,5 (2,90; 2,53E-04), RP11-44K6,4 (5,08; 1,46E-08), RP11-473M20,7 (4,16; 9,29E-27), RP11-512N21,3 (3,18; 6,46E-04), RP11-519G16,3 (3,31; 3,19E-04), RP11-58K22,4 (3,61; 9,06E-06), RP11-630D6,5 (5,47; 2,39E-06), RP11-638I2,9 (4,54; 1,18E-07), RP11-875O11,1 (2,99; 4,24E-05), RP11-89H19,1 (3,16; 3,66E-05), RP1-50J22,4 (4,16; 1,30E-06), RP1-68D18,4 (3,19; 5,32E-06), STARD4-AS1 (3,77; 3,67E-11), ZRANB2-AS1 (2,86; 1,30E-04) |
|  | processed_transcript | **Down:** LINC00263 (-4,05; 1,07E-10), LINC00899 (-3,04; 1,22E-04), SLC38A3 (-2,84; 7,03E-05), XKR5 (-3,85; 3,69E-09)  **Up:** APCDD1L-AS1 (3,17; 7,30E-05), GRIP2 (3,48; 2,83E-05), SENP3-EIF4A1 (3,03; 2,36E-04) |
|  | sense_overlapping | **Down:** GS1-358P8,4 (-3,07; 7,40E-05), RP11-462G12,1 (-3,88; 2,37E-08), RP11-462G12,2 (-2,94; 2,16E-05)  **Up:** RP11-552M11,4 (2,91; 1,36E-04) |
|  | sense_intronic | **Up:** AP001619,2 (3,20; 3,26E-04), RP11-44K6,2 (3,25; 9,41E-06) |
|  | misc_RNA | **Up:** RN7SL834P (3,84; 5,19E-06), Y_RNA (3,12; 1,48E-05) |
|  |  |  |
| **T=24h**  **588 DEGs** | protein_coding | **Down:** ABCA3 (-4,78; 1,77E-09), ACADL (-3,95; 9,49E-07), ACBD7 (-2,88; 1,28E-07), ADAMTS15 (-3,50; 4,41E-09), ADM2 (-3,22; 2,75E-04), AKAP6 (-3,31; 4,84E-05), AMIGO1 (-2,93; 5,14E-06), ARG2 (-3,75; 3,78E-08), ASNS (-2,84; 1,57E-03), C11orf87 (-5,52; 3,52E-10), CBS (-2,87; 3,36E-04), CCDC113 (-3,13; 6,80E-07), CHAC1 (-2,83; 3,39E-03), CLSTN2 (-6,05; 9,70E-17), CNTN5 (-3,42; 2,09E-05), COL21A1 (-3,70; 1,65E-05), CPA3 (-2,88; 2,78E-04), CRYAB (-4,07; 8,19E-06), CSTA (-3,32; 1,28E-06), CXADR (-2,86; 4,31E-08), CYFIP2 (-3,02; 2,04E-06), DAAM2 (-4,02; 1,40E-15), DMGDH (-3,28; 4,31E-06), DUSP1 (-4,32; 1,66E-12), ECM2 (-2,84; 4,51E-04), EFHD1 (-4,48; 4,90E-10), EGR1 (-5,26; 6,57E-13), EGR3 (-3,01; 6,17E-07), ERBB3 (-3,42; 4,31E-09), FAM131C (-3,34; 1,09E-04), FAT3 (-4,94; 3,27E-22), FBLN7 (-2,86; 8,05E-14), FOS (-19,09; 3,40E-29), FOSB (-15,51; 1,81E-35), GFRA1 (-4,65; 7,23E-13), GLRB (-3,18; 4,58E-12), GYG2 (-3,30; 1,40E-09), IGFBP5 (-2,94; 3,95E-07), IL20RB (-2,97; 2,75E-04), INHBE (-4,57; 4,79E-06), INPP5D (-4,61; 3,22E-08), ITGB4 (-3,41; 8,56E-06), KCNH7 (-4,08; 6,39E-06), KCNMB4 (-3,05; 1,32E-10), KIT (-3,87; 1,91E-09), KLF2 (-3,29; 5,72E-10), LMOD1 (-3,06; 1,06E-06), MKX (-3,73; 4,32E-10), MORN4 (-2,98; 1,98E-11), MPP7 (-2,98; 1,37E-12), MTURN (-3,05; 6,11E-24), MYCL (-2,86; 3,78E-07), MYL1 (-3,24; 2,25E-05), MYLK3 (-4,28; 6,34E-06), NLGN1 (-3,67; 1,18E-14), NR4A1 (-6,25; 6,59E-28), NRCAM (-6,33; 4,92E-11), NT5M (-2,85; 4,62E-06), OCLN (-3,02; 1,91E-12), OSR2 (-3,10; 3,02E-08), PAPPA2 (-3,00; 5,92E-05), PDE1A (-4,83; 2,08E-07), PDE1C (-2,99; 1,08E-09), PKP2 (-4,63; 7,02E-08), PODN (-2,92; 1,33E-10), PPARGC1A (-3,39; 2,14E-05), PPM1E (-3,63; 2,07E-08), PPP1R9A (-2,99; 1,55E-04), PRELP (-2,90; 4,80E-04), PRICKLE1 (-2,87; 8,89E-11), PSAT1 (-3,49; 5,08E-07), PSG5 (-2,97; 8,06E-05), PTPRQ (-3,35; 6,38E-06), RAB11FIP1 (-2,84; 1,38E-13), RBP7 (-3,12; 3,05E-04), RCAN2 (-2,94; 1,54E-08), REEP1 (-2,85; 1,36E-08), RIMS3 (-3,56; 1,48E-06), ROR1 (-2,85; 8,06E-12), SDPR (-3,34; 3,76E-06), SESN3 (-3,00; 9,53E-13), SH3BGR (-2,85; 1,07E-03), SLC16A10 (-3,21; 8,80E-10), SLC16A14 (-7,24; 9,23E-24), SLC1A2 (-3,10; 5,68E-08), SLC40A1 (-3,02; 1,34E-09), SLC7A11 (-3,26; 2,97E-05), SLITRK5 (-3,26; 1,84E-10), SMAD9 (-3,18; 2,06E-15), SMPDL3B (-3,20; 1,15E-04), ST6GALNAC2 (-2,85; 1,04E-06), STXBP6 (-2,93; 3,41E-08), TLL1 (-3,65; 2,01E-05), TM7SF2 (-3,03; 2,27E-05), TMEM170B (-3,87; 1,94E-19), TMEM56 (-2,93; 1,44E-14), TMSB15A (-2,86; 3,95E-07), TNNT2 (-3,06; 2,48E-05), TPD52L1 (-5,62; 7,25E-10), TRIB3 (-3,72; 7,83E-07), VLDLR (-3,23; 1,51E-09), ZC3H6 (-2,93; 2,98E-07), ZMAT3 (-3,38; 1,44E-23), ZNF385D (-3,45; 5,92E-11)  **Up:** ACAN (4,95; 5,67E-07), ACKR4 (2,89; 3,90E-05), ACO1 (2,83; 8,86E-50), ACSL5 (10,72; 2,45E-23), ADAP1 (6,89; 8,56E-12), AIM2 (30,09; 1,87E-17), AKR1B1 (5,68; 5,51E-27), AMPD3 (4,75; 9,42E-12), AMZ1 (3,29; 8,57E-08), ANGPT1 (3,09; 2,03E-07), ANGPTL4 (5,01; 4,77E-08), ANO4 (9,67; 9,12E-50), ANPEP (3,20; 4,96E-18), ANXA8 (3,08; 4,00E-05), ANXA8L2 (3,72; 9,24E-08), APCDD1L (2,99; 8,32E-06), APOL1 (14,26; 2,87E-94), APOL2 (5,23; 8,71E-35), APOL3 (16,77; 8,98E-85), APOL4 (64,49; 1,42E-83), APOL6 (6,77; 1,47E-68), ARID5A (3,13; 2,52E-13), BATF2 (37,78; 1,62E-54), BATF3 (3,23; 9,65E-24), BDKRB1 (11,60; 4,50E-28), BDKRB2 (5,34; 9,03E-14), BEST3 (3,92; 1,16E-06), BID (2,90; 1,55E-15), BIRC3 (8,44; 6,78E-19), BST2 (31,44; 1,39E-67), BTC (3,14; 9,52E-04), BTN3A1 (2,91; 3,40E-18), BTN3A2 (3,59; 2,43E-42), BTN3A3 (3,96; 4,03E-26), C1orf106 (2,87; 4,48E-04), C1orf147 (3,27; 3,85E-05), C1orf195 (2,83; 5,46E-04), C1QTNF1 (13,30; 1,98E-27), C1R (4,05; 8,34E-23), C1S (8,40; 1,67E-56), C2 (3,14; 6,73E-06), C20orf141 (4,28; 6,12E-07), C3 (10,81; 3,85E-64), C3AR1 (3,87; 1,38E-05), C5orf56 (2,92; 5,18E-09), C8orf34 (3,86; 5,13E-05), CACNA1I (3,05; 1,77E-03), CADPS2 (2,89; 1,44E-03), CASP4 (3,09; 2,37E-18), CCDC3 (4,13; 3,89E-22), CCL2 (28,48; 2,29E-56), CCL5 (11,26; 1,03E-12), CCL7 (8,05; 8,38E-12), CCL8 (5,14; 3,06E-06), CCND1 (3,46; 1,19E-05), CCRL2 (3,56; 1,32E-04), CD274 (7,21; 6,98E-18), CD7 (3,76; 7,00E-06), CD74 (64,52; 4,09E-54), CD82 (4,58; 2,39E-10), CD83 (4,52; 5,77E-15), CDCP1 (6,09; 5,43E-16), CEACAM1 (4,34; 8,98E-08), CFB (4,19; 2,82E-11), CFH (4,19; 4,86E-25), CH25H (3,75; 6,58E-05), CHRNA1 (14,78; 1,17E-14), CHST11 (3,88; 2,35E-09), CHST15 (4,56; 2,53E-16), CIITA (43,74; 1,36E-56), CLIC2 (14,83; 2,61E-44), CMPK2 (34,69; 3,49E-27), COL27A1 (3,22; 3,57E-08), COL7A1 (4,83; 1,88E-11), CPT1A (4,68; 2,63E-12), CSF1 (5,74; 4,43E-68), CTC-479C5,12 (4,42; 1,41E-11), CTSS (52,36; 1,89E-72), CX3CL1 (2,93; 2,90E-03), CXCL1 (14,14; 1,42E-24), CXCL10 (44,85; 2,90E-41), CXCL11 (32,45; 5,90E-33), CXCL12 (3,04; 1,09E-08), CXCL2 (3,44; 1,06E-12), CXCL3 (4,71; 3,87E-11), CXCL5 (5,44; 1,33E-10), CXCL6 (3,52; 1,03E-04), CXCL9 (20,17; 3,53E-30), DDX58 (10,59; 2,46E-79), DDX60 (6,91; 3,28E-94), DDX60L (3,86; 1,92E-41), DEC1 (3,49; 5,06E-06), DES (3,79; 4,30E-05), DEXI (2,85; 1,45E-03), DGKG (4,13; 3,24E-05), DHX58 (3,57; 1,83E-08), DLL1 (3,50; 9,42E-05), DNER (3,38; 1,25E-07), DNPEP (3,05; 1,79E-40), DPP4 (2,85; 5,45E-16), DRAM1 (4,66; 5,28E-31), DTX3L (4,95; 1,52E-74), EBI3 (4,56; 9,94E-18), EDARADD (2,86; 4,03E-08), EPSTI1 (10,38; 6,46E-63), ERAP1 (4,02; 5,51E-76), ERAP2 (4,08; 5,17E-44), ETV7 (14,96; 3,93E-50), F2RL3 (4,04; 2,19E-05), FAM101A (3,53; 7,66E-09), FAM107A (3,35; 2,87E-04), FAM115C (3,72; 4,26E-09), FAM167B (2,88; 8,55E-04), FAM26F (5,27; 4,04E-09), FBXO6 (6,16; 8,10E-10), FCGR2A (3,32; 1,53E-05), FCRL6 (4,70; 1,24E-06), FDCSP (9,40; 2,74E-12), FST (3,29; 2,24E-12), G0S2 (6,40; 3,57E-15), GBP1 (32,53; 2,40E-107), GBP2 (47,49; 1,63E-106), GBP3 (8,28; 4,57E-52), GBP4 (98,92; 6,61E-77), GBP5 (186,63; 5,36E-97), GCH1 (3,39; 4,87E-18), GFPT2 (4,19; 2,29E-09), GGT5 (4,21; 8,39E-07), GIMAP2 (4,12; 1,41E-06), GOLM1 (3,07; 3,58E-29), GPR115 (4,18; 6,13E-07), GPR39 (3,00; 7,25E-10), GPR68 (7,24; 1,89E-12), GPRC5B (3,70; 1,58E-13), GREM1 (3,64; 1,78E-10), GSAP (4,06; 9,68E-21), HAPLN3 (10,97; 4,26E-51), HAS2 (5,61; 2,11E-10), HDAC9 (3,77; 6,80E-29), HELZ2 (4,10; 3,25E-31), HERC5 (3,92; 5,88E-19), HERC6 (6,79; 3,19E-58), HLA-B (3,56; 4,95E-40), HLA-DOB (2,87; 1,37E-03), HLA-DPA1 (3,08; 4,07E-04), HLA-DRA (3,21; 8,28E-05), HLA-F (7,55; 2,20E-35), HMGA2 (3,37; 1,71E-06), HORMAD1 (3,13; 2,17E-04), HS3ST3B1 (3,23; 2,10E-11), HSD11B1 (6,22; 7,66E-09), HTR7 (2,89; 2,06E-05), ICAM1 (10,37; 4,36E-30), IDO1 (103,67; 4,35E-64), IFI27 (10,90; 1,40E-18), IFI35 (7,52; 7,69E-127), IFI44 (7,76; 1,28E-40), IFI44L (38,01; 1,57E-60), IFI6 (4,70; 1,34E-30), IFIH1 (7,85; 4,76E-52), IFIT1 (4,30; 2,88E-14), IFIT2 (8,00; 5,33E-35), IFIT3 (17,72; 8,14E-101), IFIT5 (4,22; 3,22E-62), IFITM1 (3,18; 2,55E-04), IKBKE (4,83; 4,78E-31), IL11 (5,10; 2,20E-07), IL12A (3,52; 3,35E-11), IL12RB1 (5,06; 2,30E-06), IL15 (3,03; 4,05E-09), IL15RA (14,85; 5,21E-46), IL18BP (11,18; 4,86E-100), IL1B (13,60; 7,93E-22), IL24 (3,35; 1,55E-04), IL32 (46,96; 1,16E-85), IL4I1 (4,61; 3,41E-06), IL4R (3,22; 4,48E-18), IL6 (7,48; 2,15E-14), IL7R (5,48; 6,24E-20), IL8 (11,95; 1,84E-23), INHBA (8,33; 1,94E-16), IRAK2 (6,43; 1,65E-21), IRF1 (12,92; 3,47E-62), IRF9 (3,86; 4,86E-12), IRG1 (3,07; 6,41E-06), ISG15 (8,19; 2,12E-33), ISG20 (9,22; 1,60E-22), ITGA2 (5,77; 1,88E-11), ITK (26,64; 3,60E-18), JAK2 (2,89; 1,09E-21), KCNJ15 (5,94; 1,09E-10), KIAA1217 (2,92; 1,69E-34), KIAA1644 (3,21; 1,04E-08), KIAA1755 (3,63; 1,63E-05), KLF17 (2,88; 5,78E-04), KLHDC7B (2,92; 2,02E-04), KYNU (5,50; 3,21E-07), LAMB3 (8,37; 3,59E-24), LAP3 (11,73; 5,06E-178), LGALS9 (6,66; 1,31E-10), LIMD2 (2,85; 3,26E-09), LIPG (3,04; 7,09E-05), LPXN (4,98; 2,13E-14), LRRC15 (2,96; 2,25E-04), LRRN3 (3,97; 4,13E-06), LRRTM2 (3,76; 1,54E-07), LYPD5 (3,24; 1,38E-06), MEFV (8,22; 7,98E-10), MFSD2A (2,88; 1,88E-11), MLKL (8,88; 5,83E-30), MMP1 (4,15; 1,73E-07), MMP13 (4,56; 5,08E-08), MMP2 (3,11; 2,71E-17), MMP9 (3,05; 1,59E-04), MSC (5,52; 9,23E-24), MT1E (2,96; 1,53E-08), MT1X (3,72; 2,28E-18), MT2A (6,53; 2,06E-19), MVP (2,91; 8,26E-31), MX1 (27,09; 1,33E-56), MX2 (24,15; 1,63E-30), MYEOV (3,60; 3,71E-06), MYPN (4,08; 3,88E-08), NES (2,92; 1,08E-06), NFE2L3 (4,56; 1,44E-19), NFKB1 (3,14; 7,53E-36), NFKB2 (3,16; 1,92E-21), NFKBIA (4,41; 5,77E-27), NLRC5 (5,74; 2,52E-120), NLRP3 (4,68; 6,54E-07), NMI (5,49; 1,51E-73), NRP2 (4,94; 1,37E-30), NT5E (3,08; 4,05E-08), NTM (4,60; 2,75E-07), NTN1 (3,29; 7,09E-06), NTRK3 (3,02; 4,88E-04), OAF (3,43; 1,83E-16), OAS1 (31,18; 2,96E-39), OAS2 (58,81; 2,23E-71), OAS3 (11,27; 2,25E-84), OASL (17,91; 2,60E-45), ODF3B (7,32; 1,22E-12), OSGIN1 (3,94; 1,73E-13), OTOGL (3,91; 1,16E-05), PANX2 (3,28; 1,61E-04), PARP12 (4,06; 2,27E-58), PARP14 (10,18; 5,39E-82), PARP9 (3,83; 1,52E-34), PAX5 (3,32; 5,67E-05), PAX8 (7,24; 1,96E-18), PDCD1LG2 (6,75; 7,23E-20), PDE2A (3,42; 2,01E-04), PFKFB4 (3,14; 8,58E-11), PHACTR1 (5,45; 3,11E-20), PHF11 (3,60; 6,13E-25), PHLDA1 (3,14; 3,16E-11), PIK3AP1 (3,94; 6,73E-05), PLA2G4A (3,20; 2,53E-08), PLAUR (3,32; 1,69E-10), PLEKHN1 (2,95; 5,26E-04), PLSCR1 (3,93; 6,72E-64), PML (3,93; 7,38E-61), POU2F2 (5,91; 2,97E-18), PPP4R4 (3,21; 5,10E-04), PRDM1 (3,13; 7,23E-13), PRDM8 (3,13; 5,94E-21), PRRG4 (2,96; 1,43E-07), PSMB10 (6,16; 9,10E-27), PSMB8 (4,24; 5,28E-31), PSMB9 (7,92; 1,38E-58), PSME2 (4,67; 9,61E-155), PTGES (2,93; 1,25E-04), PTPRN (3,38; 1,64E-06), RAB27B (4,85; 2,47E-18), RARRES3 (17,67; 1,90E-82), RELB (4,34; 5,58E-19), RFX8 (4,59; 7,05E-08), RHBDF2 (4,30; 3,18E-18), RIPK2 (4,50; 2,31E-60), RNF19B (3,25; 4,38E-18), ROBO4 (5,01; 1,44E-12), ROS1 (3,77; 5,70E-06), RP11-352D3,2 (6,43; 2,00E-08), RP11-366L20,2 (4,33; 1,26E-07), RP11-468E2,4 (2,94; 8,11E-06), RP11-766F14,2 (4,43; 1,05E-10), RSAD2 (10,61; 3,01E-12), RTP4 (10,33; 2,49E-14), S1PR3 (3,10; 2,48E-14), SAMD9 (4,52; 9,79E-28), SAMD9L (10,82; 2,66E-52), SAMHD1 (3,56; 2,87E-54), SCG5 (2,96; 4,23E-04), SCN3A (6,41; 1,83E-07), SECTM1 (5,32; 1,28E-14), SELPLG (5,40; 5,92E-11), SEMA3C (3,36; 1,21E-29), SEMA4D (10,03; 4,22E-24), SEMA7A (3,36; 6,40E-07), SERPINA1 (7,12; 1,00E-30), SERPINB2 (19,57; 1,15E-31), SERPINB8 (3,15; 2,53E-16), SERPINE2 (3,17; 6,61E-10), SERPING1 (3,53; 3,15E-21), SLAMF8 (15,57; 3,99E-22), SLC15A3 (9,78; 6,82E-82), SLC28A3 (4,16; 1,88E-06), SLC2A5 (3,70; 7,05E-06), SLC2A6 (6,22; 1,67E-29), SLC39A14 (2,93; 2,37E-31), SLC6A6 (2,91; 2,41E-11), SLIT2 (3,46; 7,75E-12), SMTNL1 (7,19; 2,16E-13), SOCS1 (9,04; 1,15E-23), SOD2 (8,33; 3,11E-22), SP100 (3,67; 1,54E-62), SP110 (4,44; 2,85E-45), SPRY4 (4,11; 1,74E-13), SRGN (2,87; 4,98E-23), SSTR2 (3,93; 3,55E-07), STAT1 (9,86; 1,41E-206), STAT2 (2,89; 7,23E-24), STC1 (4,15; 1,34E-09), STEAP1 (3,17; 3,88E-04), TAP1 (15,70; 3,41E-108), TAP2 (5,40; 2,11E-57), TAPBP (3,96; 2,05E-69), TAPBPL (4,18; 1,26E-29), TBX2 (3,74; 2,81E-13), TDO2 (3,56; 1,04E-04), TEX29 (3,81; 9,10E-06), TFPI2 (4,68; 4,46E-10), TGM2 (9,86; 6,43E-21), TLDC2 (4,69; 2,87E-13), TLR4 (3,89; 1,11E-22), TMEM119 (3,21; 5,54E-07), TMEM132A (4,11; 1,70E-18), TMEM158 (6,52; 2,24E-12), TMEM171 (4,14; 2,89E-29), TMEM229B (4,38; 7,13E-08), TMEM239 (3,62; 1,23E-05), TMEM51 (3,09; 2,55E-22), TMTC1 (2,84; 9,79E-21), TNC (9,05; 4,77E-21), TNFAIP2 (9,25; 9,46E-38), TNFAIP3 (13,90; 5,08E-27), TNFAIP6 (2,87; 1,27E-06), TNFRSF14 (7,07; 5,31E-18), TNFRSF1B (3,29; 1,21E-20), TNFRSF8 (5,60; 3,59E-13), TNFSF10 (10,82; 4,36E-21), TNFSF13B (9,59; 1,92E-13), TNFSF15 (4,44; 5,84E-06), TNIP1 (4,40; 5,80E-29), TNIP3 (7,65; 9,95E-12), TP63 (3,49; 2,70E-05), TRAF1 (7,25; 3,67E-15), TRANK1 (3,27; 3,24E-12), TRIB2 (3,22; 2,49E-11), TRIM16 (2,96; 2,54E-13), TRIM16L (2,87; 6,00E-11), TRIM21 (4,08; 1,23E-31), TRIM22 (4,54; 1,87E-17), TRIM25 (2,94; 6,83E-24), TRIM69 (3,86; 3,37E-34), TRPA1 (3,25; 1,24E-06), TSKU (3,03; 5,30E-17), TTLL6 (3,46; 1,92E-04), TTYH1 (2,86; 2,16E-04), TYMP (5,87; 2,87E-21), UBD (8,26; 2,60E-10), UBE2L6 (7,12; 1,21E-111), UCN2 (4,79; 7,51E-09), USP18 (19,33; 1,64E-26), VCAM1 (5,99; 1,47E-15), VEGFC (4,40; 3,59E-39), VEPH1 (4,81; 2,71E-17), VNN1 (4,88; 1,05E-07), WARS (7,81; 2,00E-52), WT1 (2,90; 4,39E-07), XAF1 (9,65; 4,34E-60), XIRP1 (14,21; 8,76E-24), ZC3H12A (4,37; 2,05E-10), ZNFX1 (3,01; 4,74E-32) |
|  | lincRNA | **Down:** AC007405,6 (-2,92; 4,35E-04), RP11-495P10,8 (-3,86; 5,78E-05), SFTA1P (-5,53; 2,11E-10), snoU13 (-2,87; 3,40E-05)  **Up:** AC003092,1 (4,25; 3,46E-06), AC131025,8 (3,15; 5,87E-07), AP001422,3 (3,07; 2,47E-05), CTD-2521M24,9 (3,09; 5,88E-06), LINC00619 (2,86; 4,02E-08), LINC00856 (3,84; 2,00E-04), LINC00941 (2,87; 5,35E-09), LINC00942 (3,81; 1,04E-06), LINC00973 (2,89; 3,44E-04), LUCAT1 (3,45; 4,58E-05), MIR146A (3,73; 4,77E-08), MIR155HG (4,59; 2,16E-13), MIR29A (2,94; 9,67E-08), RP11-148B18,3 (2,92; 1,18E-03), RP11-154H12,2 (8,11; 2,67E-11), RP11-154H23,3 (2,88; 3,82E-05), RP11-245M24,1 (4,01; 2,12E-06), RP11-333O1,1 (3,16; 3,14E-04), RP11-383J24,1 (2,98; 2,71E-04), RP11-400K9,4 (3,00; 2,51E-10), RP11-527H14,3 (2,91; 1,69E-05), RP11-532F6,3 (2,85; 6,57E-04), RP11-79H23,3 (9,17; 3,96E-12), RP11-865I6,2 (2,90; 5,32E-05), RP11-91K9,1 (13,01; 1,46E-14), RP13-297E16,4 (3,11; 4,95E-04), RP4-794H19,4 (3,78; 2,61E-07), TAPSAR1 (3,24; 3,22E-15) |
|  | SnoRNA | **Up:** SNORA51 (3,43; 5,26E-05) |
|  | pseudogene | **Down:** ASNSP1 (-3,34; 4,70E-04), ECEL1P2 (-4,19; 1,99E-05), RP1-199J3,5 (-3,20; 1,96E-04)  **Up:** AC006028,11 (3,47; 3,98E-05), AC007362,1 (4,29; 2,79E-20), AC067945,3 (3,11; 3,43E-04), CMAHP (4,85; 2,32E-16), GBP1P1 (79,34; 2,68E-61), HLA-H (3,56; 1,43E-21), HSPA8P11 (3,40; 3,04E-04), KRT121P (2,86; 5,44E-04), LGALS17A (12,20; 6,39E-12), MT2P1 (6,60; 3,58E-10), MTCYBP3 (2,97; 3,33E-04), OR2I1P (3,88; 1,51E-07), PGAM1P7 (3,07; 3,19E-04), PTGES3P1 (12,51; 3,53E-27), RP11-274E7,2 (3,13; 3,05E-04), RP11-322D14,1 (3,23; 1,24E-04), RP11-383F6,1 (4,12; 3,52E-07), RP11-638I2,10 (4,74; 1,36E-08), RPL21P121 (2,87; 9,14E-04) |
|  | antisense | **Down:** PLCE1-AS1 (-5,80; 1,71E-08), RP11-125B21,2 (-4,19; 8,11E-06), RP11-390P2,4 (-3,05; 2,39E-04), RP11-3L8,3 (-3,71; 5,65E-05), RP11-558F24,4 (-5,58; 8,22E-12), ZBED3-AS1 (-3,02; 3,83E-05)  **Up:** AC006160,5 (6,29; 3,75E-12), AC007362,3 (3,51; 3,90E-06), AC008063,2 (3,75; 1,43E-04), AC009950,2 (3,66; 1,78E-12), AC020571,3 (4,71; 9,09E-08), AC116366,6 (3,32; 6,34E-08), AF127577,11 (2,94; 1,39E-03), C1RL-AS1 (2,95; 7,39E-17), CTD-2031P19,3 (4,62; 1,96E-09), CTD-2240E14,4 (3,16; 1,90E-04), CTD-2260A17,1 (3,63; 9,20E-12), CTD-2587H24,5 (3,41; 4,97E-08), INHBA-AS1 (3,62; 5,57E-05), RP11-134L10,1 (3,66; 5,70E-06), RP11-142M10,2 (3,04; 5,32E-05), RP11-165P7,1 (2,86; 3,26E-04), RP11-288L9,4 (4,23; 1,76E-05), RP11-313E19,2 (3,20; 5,29E-05), RP11-327F22,2 (3,12; 2,54E-04), RP11-356I2,4 (4,61; 4,26E-08), RP11-44K6,4 (5,76; 6,96E-09), RP11-519G16,3 (3,50; 2,74E-04), RP11-58K22,4 (3,18; 7,96E-05), RP11-630D6,5 (2,88; 1,61E-03), RP11-638I2,9 (5,31; 9,15E-09), RP1-50J22,4 (4,55; 7,48E-07), RP3-508I15,19 (3,11; 5,85E-04), ZMIZ1-AS1 (2,88; 5,88E-05) |
|  | processed_transcript | **Up:** APCDD1L-AS1 (3,51; 2,58E-05), GRIP2 (5,44; 9,92E-07) |
|  | sense_intronic | **Down:** MIR600HG (-3,47; 3,55E-09)  **Up:** RP11-404P21,5 (3,43; 8,28E-05), RP11-44K6,2 (2,92; 3,67E-05) |
|  | Mt-RNA | **Up:** MT-RNR1 (2,91; 1,25E-05), MT-RNR2 (3,02; 1,78E-06) |
|  | misc_RNA | **Up:** RN7SL834P (3,17; 1,58E-04) |
|  | rRNA | **Up:** RNA18S5 (4,85; 1,30E-13), RNA28S5 (3,74; 9,29E-10) |
|  | Ig-V gene | **Up:** IGKV1OR2-108 (2,87; 7,74E-04) |
|  |  |  |
| **T=48h**  **234 DEGs** | protein_coding | **Down:** ACBD7 (-3,04; 3,25E-08), AQP3 (-2,89; 4,69E-09), ATP1B2 (-3,02; 2,11E-08), CLSTN2 (-4,32; 4,45E-11), CRYAB (-2,94; 3,60E-03), CYP27C1 (-3,29; 1,09E-07), DUSP1 (-3,37; 1,75E-08), EGR1 (-5,22; 1,46E-12), EGR3 (-4,28; 1,57E-11), FGFR3 (-3,25; 6,97E-15), FOS (-16,00; 4,28E-25), FOSB (-13,64; 7,57E-32), GFRA1 (-2,94; 2,62E-06), GPR155 (-2,85; 3,30E-11), ID1 (-3,72; 3,96E-09), IL3RA (-2,86; 1,60E-04), INPP5D (-3,70; 4,45E-06), KCNMB4 (-2,95; 6,69E-10), METTL7A (-3,19; 8,77E-06), MTURN (-2,85; 1,74E-21), MXD3 (-3,68; 2,65E-15), MYLK3 (-3,92; 2,92E-05), NR4A1 (-9,65; 3,21E-42), NR4A2 (-4,33; 6,69E-10), NR4A3 (-4,49; 4,26E-14), OLFM2 (-2,84; 1,51E-04), PLEKHB1 (-3,25; 6,61E-06), RAB3A (-3,03; 5,67E-06), RLTPR (-2,87; 1,71E-05), SEPT3 (-2,85; 1,19E-04), SESN3 (-3,63; 3,58E-17), TNFRSF10D (-4,43; 2,03E-29), ZMAT3 (-3,81; 3,29E-28)  **Up:** ACSL5 (7,37; 1,39E-16), ADAMTS6 (2,99; 5,89E-21), ADAP1 (2,95; 2,84E-04), AIM2 (13,48; 2,51E-12), ANGPT1 (3,29; 4,95E-08), ANO4 (5,96; 2,29E-30), APOL1 (7,43; 4,35E-53), APOL3 (5,97; 1,50E-33), APOL4 (22,47; 1,11E-47), APOL6 (4,86; 2,17E-46), B2M (3,07; 9,48E-36), BATF2 (15,46; 6,58E-32), BDKRB1 (4,77; 1,70E-11), BDKRB2 (2,87; 1,13E-05), BST2 (22,91; 2,16E-55), C1QTNF1 (3,33; 2,70E-06), C1R (3,97; 6,28E-22), C1S (7,36; 3,61E-49), C3 (7,56; 6,72E-46), C8orf34 (3,36; 3,19E-04), CCL2 (7,86; 1,76E-21), CCL5 (5,27; 1,06E-07), CCRL2 (2,88; 1,04E-03), CD274 (3,02; 7,69E-06), CD74 (46,17; 1,89E-46), CDCP1 (4,66; 1,61E-11), CETP (2,93; 2,31E-04), CH25H (3,21; 6,05E-04), CHRNA1 (6,05; 2,63E-09), CHST15 (2,91; 4,62E-08), CIITA (15,46; 3,42E-31), CLIC2 (6,47; 4,50E-21), CMPK2 (10,79; 1,16E-14), CTSS (20,02; 3,44E-42), CXCL1 (8,98; 1,17E-17), CXCL10 (5,77; 5,53E-14), CXCL11 (5,99; 8,52E-13), CXCL2 (2,88; 3,07E-09), CXCL3 (3,45; 3,71E-07), CXCL5 (3,96; 4,19E-07), CXCL9 (4,66; 5,90E-15), DDX58 (4,70; 1,47E-33), DDX60 (5,86; 3,43E-78), DDX60L (3,02; 1,95E-27), DPP4 (2,88; 3,79E-16), DTX3L (4,04; 5,09E-56), EPSTI1 (6,15; 1,91E-37), ERAP1 (3,12; 3,67E-50), ERAP2 (2,93; 2,04E-25), ETV7 (4,25; 4,89E-14), EVI2B (3,80; 1,00E-09), FAIM3 (3,22; 1,90E-09), FAM167A (3,76; 6,32E-19), FAM20A (2,93; 5,68E-08), FBXO6 (3,22; 1,22E-04), FDCSP (3,75; 2,03E-07), FST (2,91; 7,81E-10), G0S2 (4,97; 2,87E-11), GATA3 (3,04; 4,57E-12), GBP1 (11,94; 6,00E-54), GBP2 (16,02; 9,36E-55), GBP3 (3,98; 7,87E-22), GBP4 (26,17; 1,82E-42), GBP5 (66,41; 2,17E-65), GREM1 (3,48; 1,29E-09), HAPLN3 (2,88; 1,09E-09), HAS2 (6,99; 1,12E-12), HERC6 (4,11; 2,66E-31), HLA-A (2,93; 3,74E-27), HLA-B (4,08; 9,70E-49), HLA-C (3,48; 4,50E-39), HLA-DRA (3,22; 1,11E-04), HLA-F (7,35; 2,64E-34), HMGA2 (3,01; 2,55E-05), HSD11B1 (3,30; 1,14E-05), ICAM1 (3,02; 8,46E-07), IDO1 (28,73; 2,00E-37), IFI27 (12,51; 2,11E-20), IFI35 (5,18; 2,67E-83), IFI44 (3,48; 9,23E-15), IFI44L (15,05; 4,79E-34), IFI6 (4,40; 7,90E-28), IFIH1 (3,59; 1,37E-19), IFIT2 (3,94; 5,50E-15), IFIT3 (8,51; 3,45E-55), IFIT5 (3,26; 1,99E-41), IL15RA (7,87; 9,98E-27), IL18BP (5,98; 4,67E-54), IL1B (5,44; 6,25E-10), IL24 (4,61; 2,08E-06), IL32 (12,14; 3,01E-36), IL6 (3,90; 6,63E-07), IL7R (4,41; 5,94E-15), IL8 (5,90; 7,08E-14), INHBA (3,65; 1,57E-06), IRF1 (4,62; 8,18E-22), ISG15 (4,76; 3,82E-18), ITGA2 (2,92; 1,19E-04), ITK (8,99; 4,16E-11), KCNJ15 (3,16; 8,84E-06), KLHDC7B (4,42; 1,80E-07), KRT34 (5,31; 1,25E-07), LAMB3 (3,61; 7,66E-09), LAP3 (5,17; 3,38E-78), LGALS9 (4,05; 2,29E-06), LURAP1L (2,87; 6,82E-05), MLKL (3,33; 4,58E-09), MX1 (12,63; 1,66E-33), MX2 (13,24; 6,05E-21), MYEOV (4,61; 3,25E-08), NGEF (2,85; 2,22E-04), NLRC5 (4,02; 1,41E-75), NLRP10 (7,05; 6,11E-08), NMI (3,86; 1,06E-45), OAS1 (11,22; 2,51E-20), OAS2 (22,38; 2,59E-42), OAS3 (4,99; 9,00E-37), OASL (8,95; 4,41E-26), ODF3B (5,47; 2,31E-09), OSGIN1 (4,03; 8,57E-14), PARP14 (4,42; 3,51E-33), PARP9 (2,91; 1,20E-21), PAX8 (4,01; 3,71E-09), PDCD1LG2 (5,46; 1,60E-15), POU2F2 (3,21; 6,20E-08), PRKCH (3,91; 7,61E-07), PSMB10 (3,76; 5,90E-14), PSMB8 (3,03; 5,85E-18), PSMB9 (6,16; 9,15E-45), PSME2 (3,39; 5,83E-96), PTPRC (3,05; 3,63E-03), PTPRN (3,49; 1,28E-06), PTPRR (2,96; 4,84E-05), PTX3 (3,38; 1,48E-07), RAB27B (4,22; 5,24E-15), RARRES3 (9,89; 6,07E-52), RFX8 (3,45; 2,17E-05), ROBO4 (3,15; 1,68E-06), RP11-352D3,2 (3,71; 1,50E-05), RSAD2 (8,92; 1,14E-10), RTP4 (3,91; 8,39E-06), SAMD3 (2,86; 2,90E-03), SAMD9L (4,08; 6,99E-18), SCN3A (4,92; 4,73E-06), SELPLG (2,95; 7,51E-05), SEMA3C (2,95; 2,13E-23), SERPINA1 (3,08; 5,71E-10), SERPINB2 (8,43; 1,33E-18), SERPING1 (3,60; 1,26E-21), SLAMF8 (4,43; 6,69E-11), SLC15A3 (4,29; 6,89E-33), SLC28A3 (2,90; 5,48E-04), SMTNL1 (4,24; 1,49E-07), SOCS1 (3,06; 3,16E-06), SOD2 (2,88; 1,03E-05), STAT1 (6,79; 4,62E-144), TAP1 (6,29; 1,08E-47), TAP2 (3,10; 2,01E-25), TAPBPL (4,00; 1,00E-27), TGM2 (4,75; 7,07E-10), TMEM158 (4,80; 7,84E-09), TMEM171 (3,11; 2,69E-18), TNC (3,76; 8,45E-08), TNFAIP3 (5,04; 1,22E-10), TNFRSF14 (3,22; 1,21E-06), TNFRSF8 (2,85; 3,63E-05), TNFSF15 (2,91; 1,33E-03), TNIP3 (3,22; 7,64E-06), TRIM69 (3,15; 2,26E-24), TYMP (3,54; 9,43E-11), UBE2L6 (5,40; 9,10E-82), USP18 (6,78; 6,06E-12), VEGFC (3,28; 7,84E-25), VEPH1 (2,99; 2,11E-08), WARS (6,66; 2,87E-44), XAF1 (5,74; 2,89E-35), XIRP1 (4,25; 4,01E-10) |
|  | lincRNA | **Down:** RP11-480I12,7 (-2,83; 1,91E-04)  **Up:** AC003092,1 (2,85; 3,07E-04), AC093642,4 (3,57; 9,96E-03), CTD-2521M24,9 (3,31; 1,90E-06), LINC00619 (3,96; 1,95E-13), LINC00942 (2,86; 2,49E-04), RP11-154H12,2 (3,84; 4,63E-07), RP11-245M24,1 (3,40; 4,15E-05), RP11-79H23,3 (3,10; 4,76E-06), RP11-91K9,1 (5,07; 7,27E-09), RP3-471M13,2 (2,91; 4,06E-03) |
|  | pseudogene | **Down:** CTA-963H5,5 (-3,07; 4,70E-04), ECEL1P2 (-3,23; 1,08E-03), RP11-396K3,1 (-2,97; 9,80E-06)  **Up:** GBP1P1 (36,24; 4,46E-43), HLA-H (3,20; 6,40E-18), LGALS17A (4,63; 3,79E-07), PTGES3P1 (3,12; 6,64E-06), RP11-638I2,10 (3,38; 1,84E-05) |
|  | antisense | **Down:** PLCE1-AS1 (-2,90; 6,72E-03), RP11-531F16,4 (-2,85; 3,38E-05)  **Up:** CTA-384D8,31 (3,12; 3,73E-03), ZMIZ1-AS1 (3,05; 2,74E-05) |
|  | sense_overlapping | **Down:** RP11-119F7,5 (-2,94; 1,93E-08) |

(Fold change; Adjusted p-value)
