# Supplementary material for: Co-Exposure of Cardiomyocytes to IFN-γ and TNF-α Induces Mitochondrial Dysfunction and Nitro-Oxidative Stress: Implications for the Pathogenesis of Chronic Chagas Disease Cardiomyopathy
Source: Front Immunol. 2021 Nov 11;12:755862. doi: 10.3389/fimmu.2021.755862 (PMC8632642; doi:10.3389/fimmu.2021.755862)
Supplement: Supplementary file 8 [file Table_3.docx]

**Supplementary Table 3:** Number of DEGs for each main pathway at each time point

| **KEGG pathways** | **T=1h** | **T=6h** | **T=12h** | **T=24h** | **T=48h** |
| --- | --- | --- | --- | --- | --- |
| Cytokine-cytokine receptor interaction hsa04060 | 6 | 49 | 45 | 37 | 21 |
| Metabolic pathways hsa01100 | 5 | 43 | 59 | 36 | 7 |
| NOD-like receptor signaling pathway hsa04621 | 10 | 31 | 29 | 28 | 18 |
| TNF signaling pathway hsa04668 | 10 | 34 | 28 | 25 | 14 |
| PI3K-Akt signaling pathway hsa04151 | 3 | 30 | 25 | 18 | 10 |
| NF-kappa B signaling pathway hsa04064 | 6 | 25 | 21 | 20 | 8 |
| Chemokine signaling pathway hsa04062 | 6 | 21 | 21 | 20 | 11 |
| IL-17 signaling pathway hsa04657 | 7 | 18 | 18 | 19 | 12 |
| Jak-STAT signaling pathway hsa04630 | 6 | 24 | 19 | 16 | 7 |
| MAPK signaling pathway hsa04010 | 5 | 23 | 19 | 14 | 8 |
| Toll-like receptor signaling pathway hsa04620 | 3 | 18 | 14 | 12 | 8 |
| Calcium signaling pathway hsa04020 | 5 | 18 | 14 | 10 | 5 |
| Chagas disease hsa05142 | 3 | 17 | 13 | 11 | 7 |
| Th17 cell differentiation hsa04659 | 1 | 17 | 14 | 12 | 6 |
| Th1 and Th2 cell differentiation hsa04658 | 1 | 14 | 12 | 12 | 4 |
| TGF-beta signaling pathway hsa04350 | 2 | 10 | 9 | 5 | 4 |
| African trypanosomiasis hsa05143 | 2 | 8 | 6 | 7 | 5 |
| Arrhythmogenic right ventricular cardiomyopathy hsa05412 | 1 | 3 | 5 | 4 | 1 |
| Wnt signaling pathway hsa04310 | 4 | 7 | 6 | 4 |  |
| Hypertrophic cardiomyopathy hsa05410 |  | 4 | 6 | 5 | 2 |
| Dilated cardiomyopathy hsa05414 |  | 3 | 4 | 4 | 1 |
|  |  |  |  |  |  |
| **IPA ingenuity pathways** | **T=1h** | **T=6h** | **T=12h** | **T=24h** | **T=48h** |
| Interferon Signaling | 6 | 13 | 11 | 16 | 11 |
| HMGB1 Signaling | 6 | 32 | 24 | 17 | 7 |
| IL-17 Signaling | 4 | 29 | 22 | 20 | 10 |
| TREM1 Signaling | 3 | 19 | 15 | 15 | 9 |
| Hepatic Fibrosis Signaling | 13 | 38 | 19 | 22 | 10 |
| IL-6 Signaling | 3 | 21 | 14 | 11 | 5 |
| Th1 and Th2 Activation | 4 | 24 | 20 | 18 | 12 |
| Role of JAK1 and JAK3 in γc Cytokine Signaling Pathway | 3 | 15 | 14 | 7 | 4 |
| Role of PKR in Interferon Induction and Antiviral Response | 4 | 19 | 15 | 14 | 7 |
| Role of MAPK Signaling in Inhibiting the Pathogenesis of Influenza | 3 | 15 | 14 | 11 | 6 |
| Th1 | 4 | 18 | 18 | 16 | 11 |
| TNFR2 Signaling | 2 | 10 | 9 | 8 | 2 |
| IL-17A Signaling in Fibroblasts | 2 | 9 | 10 | 9 | 4 |
| iNOS Signaling | 2 | 11 | 11 | 9 | 3 |
| HOTAIR regulatory | 7 | 11 | 12 | 10 |  |
| PI3K/AKT Signaling |  | 16 | 20 | 13 | 5 |
| NF-κB Signaling |  | 25 | 20 | 11 |  |
| IL-15 Production | 3 | 17 | 9 | 13 | 5 |
| Cardiac Hypertrophy Signaling (Enhanced) | 7 | 37 | 33 | 25 | 10 |
| Production of Nitric Oxide and Reactive Oxygen Species in Macrophages | 6 | 20 | 16 | 12 | 3 |
| Differential Regulation of Cytokine Production in Intestinal Epithelial Cells by IL-17A and IL-17F | 2 | 7 | 4 | 4 | 3 |
| Role of JAK2 in Hormone-like Cytokine Signaling Pathway | 3 | 7 | 4 | 3 | 2 |
| Role of JAK family kinases in IL-6-type Cytokine Signaling Pathway | 2 | 6 | 6 | 4 | 3 |
| IL-8 Signaling | 7 | 17 | 17 | 12 | 7 |
| IL-12 Signaling and Production in Macrophages | 3 | 13 | 12 | 10 | 6 |
| IL-7 Signaling | 3 | 9 | 8 | 5 | 3 |
| Differential Regulation of Cytokine Production in Macrophages and T Helper Cells by IL-17A and IL-17F | 2 | 7 | 4 | 4 | 4 |
