# Supplementary material for: Co-Exposure of Cardiomyocytes to IFN-γ and TNF-α Induces Mitochondrial Dysfunction and Nitro-Oxidative Stress: Implications for the Pathogenesis of Chronic Chagas Disease Cardiomyopathy
Source: Front Immunol. 2021 Nov 11;12:755862. doi: 10.3389/fimmu.2021.755862 (PMC8632642; doi:10.3389/fimmu.2021.755862)
Supplement: Supplementary file 9 [file Table_4.docx]

**Supplementary Table 4 :** List of the DEGs shared between the various experiments.

| **Name** | **IFN/TNF stimulation** | **IFN**  **stimulation** | **TNF**  **stimulation** |
| --- | --- | --- | --- |
| **AARD** |  |  | DEG |
| **AARS** | DEG | DEG | DEG |
| **ABCA1** |  |  | DEG |
| **ABCA3** | DEG | DEG | DEG |
| **ABCA7** | DEG |  |  |
| **ABCA8** | DEG |  | DEG |
| **ABCG4** | DEG |  |  |
| **ABI3** | DEG |  |  |
| **ABL2** | DEG | DEG | DEG |
| **ABTB2** | DEG |  | DEG |
| **AC002454,1** | DEG |  |  |
| **AC003092,1** | DEG |  |  |
| **AC004837,5** | DEG |  |  |
| **AC005682,6** | DEG | DEG |  |
| **AC006028,11** | DEG |  | DEG |
| **AC006159,5** | DEG | DEG | DEG |
| **AC006160,5** | DEG | DEG | DEG |
| **AC006273,5** | DEG |  | DEG |
| **AC006460,2** | DEG |  |  |
| **AC007050,17** |  | DEG | DEG |
| **AC007099,1** | DEG |  | DEG |
| **AC007246,3** | DEG |  |  |
| **AC007362,1** | DEG |  | DEG |
| **AC007362,3** | DEG | DEG | DEG |
| **AC007405,6** | DEG | DEG | DEG |
| **AC007421,1** |  |  | DEG |
| **AC007750,5** | DEG | DEG | DEG |
| **AC008063,2** | DEG |  |  |
| **AC009133,12** | DEG |  |  |
| **AC009502,4** | DEG |  |  |
| **AC009950,2** | DEG | DEG |  |
| **AC009951,1** |  |  | DEG |
| **AC010492,2** | DEG |  |  |
| **AC011242,6** |  |  | DEG |
| **AC012360,6** | DEG | DEG |  |
| **AC015849,13** |  |  | DEG |
| **AC015849,19** |  | DEG |  |
| **AC017002,2** | DEG | DEG | DEG |
| **AC019117,2** | DEG |  |  |
| **AC020571,3** | DEG | DEG | DEG |
| **AC064852,5** |  |  | DEG |
| **AC067945,3** | DEG |  |  |
| **AC068491,3** | DEG | DEG | DEG |
| **AC073130,1** |  | DEG | DEG |
| **AC074338,4** |  |  | DEG |
| **AC079781,5** | DEG | DEG | DEG |
| **AC083884,8** |  |  | DEG |
| **AC090673,2** | DEG | DEG | DEG |
| **AC092168,2** |  |  | DEG |
| **AC093642,4** | DEG |  |  |
| **AC096772,6** | DEG |  |  |
| **AC104654,2** |  |  | DEG |
| **AC105206,1** |  |  | DEG |
| **AC106786,1** |  | DEG |  |
| **AC106801,1** |  |  | DEG |
| **AC112721,1** |  | DEG | DEG |
| **AC116366,6** | DEG | DEG | DEG |
| **AC117395,1** | DEG |  |  |
| **AC131025,8** | DEG | DEG | DEG |
| **AC133644,3** | DEG | DEG | DEG |
| **ACACB** | DEG |  |  |
| **ACADL** | DEG | DEG | DEG |
| **ACAN** | DEG | DEG |  |
| **ACBD4** | DEG |  | DEG |
| **ACBD7** | DEG |  | DEG |
| **ACKR4** | DEG |  |  |
| **ACO1** | DEG |  |  |
| **ACPT** | DEG |  | DEG |
| **ACSL5** | DEG | DEG | DEG |
| **ACSS2** |  |  | DEG |
| **ACSS3** |  | DEG | DEG |
| **ACTA2** |  |  | DEG |
| **ADAM12** |  |  | DEG |
| **ADAM1B** | DEG |  |  |
| **ADAM22** | DEG |  |  |
| **ADAM33** | DEG |  | DEG |
| **ADAMTS1** | DEG | DEG | DEG |
| **ADAMTS13** | DEG |  | DEG |
| **ADAMTS15** | DEG |  | DEG |
| **ADAMTS6** | DEG |  |  |
| **ADAMTSL4** |  |  | DEG |
| **ADAP1** | DEG | DEG |  |
| **ADM** |  |  | DEG |
| **ADM2** | DEG | DEG | DEG |
| **ADORA1** | DEG |  | DEG |
| **ADRB2** | DEG | DEG | DEG |
| **ADTRP** |  |  | DEG |
| **AF124730,4** |  | DEG |  |
| **AF127577,11** | DEG |  |  |
| **AF129075,5** |  | DEG |  |
| **AIF1L** | DEG | DEG |  |
| **AIG1** | DEG |  |  |
| **AIM1** | DEG |  |  |
| **AIM2** | DEG | DEG |  |
| **AJUBA** | DEG |  | DEG |
| **AK7** |  | DEG |  |
| **AKAP6** | DEG | DEG | DEG |
| **AKNA** | DEG | DEG | DEG |
| **AKR1B1** | DEG |  | DEG |
| **AKR1C2** | DEG |  | DEG |
| **AKR1C3** | DEG | DEG | DEG |
| **AL137026,1** |  |  | DEG |
| **AL158147,2** |  |  | DEG |
| **AL353583,1** | DEG | DEG | DEG |
| **AL590452,1** | DEG |  | DEG |
| **AL603965,1** |  |  | DEG |
| **ALCAM** | DEG |  | DEG |
| **ALDH1L2** | DEG | DEG | DEG |
| **ALDH3B1** | DEG |  | DEG |
| **ALDH4A1** | DEG |  |  |
| **ALDH5A1** | DEG |  |  |
| **AMIGO1** | DEG | DEG | DEG |
| **AMIGO2** |  |  | DEG |
| **AMOT** |  | DEG |  |
| **AMPD3** | DEG |  | DEG |
| **AMZ1** | DEG |  | DEG |
| **ANG** |  |  | DEG |
| **ANGPT1** | DEG | DEG |  |
| **ANGPTL4** | DEG | DEG | DEG |
| **ANK2** | DEG | DEG | DEG |
| **ANKEF1** |  |  | DEG |
| **ANKRD2** |  | DEG |  |
| **ANKRD34A** | DEG |  | DEG |
| **ANKRD55** | DEG |  |  |
| **ANO4** | DEG | DEG |  |
| **ANPEP** | DEG |  |  |
| **ANTXR2** | DEG |  | DEG |
| **ANXA10** | DEG | DEG | DEG |
| **ANXA8** | DEG |  | DEG |
| **ANXA8L1** | DEG |  | DEG |
| **ANXA8L2** | DEG |  | DEG |
| **ANXA9** | DEG |  | DEG |
| **AP000240,9** | DEG | DEG | DEG |
| **AP000593,6** | DEG |  |  |
| **AP000695,1** | DEG | DEG | DEG |
| **AP000695,4** |  |  | DEG |
| **AP000695,6** |  |  | DEG |
| **AP001422,3** | DEG |  | DEG |
| **AP001619,2** | DEG |  |  |
| **AP001631,10** |  |  | DEG |
| **APBA1** | DEG |  |  |
| **APCDD1** |  |  | DEG |
| **APCDD1L** | DEG | DEG | DEG |
| **APCDD1L-AS1** | DEG | DEG | DEG |
| **APOL1** | DEG | DEG |  |
| **APOL2** | DEG | DEG |  |
| **APOL3** | DEG | DEG |  |
| **APOL4** | DEG | DEG |  |
| **APOL6** | DEG | DEG |  |
| **AQP3** | DEG |  |  |
| **ARG2** | DEG |  |  |
| **ARHGAP22** |  |  | DEG |
| **ARHGAP31** | DEG |  | DEG |
| **ARHGAP42** | DEG |  |  |
| **ARHGAP9** | DEG | DEG | DEG |
| **ARHGDIB** |  |  | DEG |
| **ARHGEF16** | DEG |  | DEG |
| **ARID5A** | DEG |  |  |
| **ARMC4** | DEG |  |  |
| **ART3** | DEG | DEG |  |
| **ARVCF** | DEG |  | DEG |
| **ASB2** | DEG |  |  |
| **ASB5** |  | DEG |  |
| **ASIC1** | DEG |  | DEG |
| **ASNS** | DEG | DEG | DEG |
| **ASNSP1** | DEG | DEG | DEG |
| **ATF3** | DEG | DEG | DEG |
| **ATP1B2** | DEG | DEG |  |
| **ATP2A3** |  |  | DEG |
| **ATP2B1** | DEG |  | DEG |
| **ATP6V0E2** | DEG |  | DEG |
| **ATP6V1G2** | DEG |  | DEG |
| **ATRNL1** |  | DEG |  |
| **B2M** | DEG | DEG |  |
| **B3GNT5** |  |  | DEG |
| **B3GNT7** |  | DEG |  |
| **B4GALT1** | DEG |  | DEG |
| **BACH1-IT3** |  |  | DEG |
| **BATF2** | DEG | DEG |  |
| **BATF3** | DEG | DEG |  |
| **BCL2A1** | DEG |  |  |
| **BCL3** | DEG | DEG | DEG |
| **BCL6** | DEG | DEG |  |
| **BDKRB1** | DEG | DEG | DEG |
| **BDKRB2** | DEG |  | DEG |
| **BEST1** |  | DEG |  |
| **BEST3** | DEG | DEG |  |
| **BHLHE40** | DEG |  |  |
| **BHLHE41** |  |  | DEG |
| **BHMT** |  | DEG |  |
| **BID** | DEG |  | DEG |
| **BIRC2** |  |  | DEG |
| **BIRC3** | DEG | DEG | DEG |
| **BLZF2P** | DEG | DEG | DEG |
| **BMP4** | DEG |  | DEG |
| **BMPER** | DEG |  | DEG |
| **BMPR1B** | DEG | DEG | DEG |
| **BST2** | DEG | DEG |  |
| **BTC** | DEG |  |  |
| **BTG2** |  | DEG |  |
| **BTN3A1** | DEG | DEG |  |
| **BTN3A2** | DEG | DEG |  |
| **BTN3A3** | DEG | DEG |  |
| **C10orf10** | DEG |  |  |
| **C10orf54** |  |  | DEG |
| **C10orf85** |  | DEG | DEG |
| **C11orf87** | DEG | DEG | DEG |
| **C11orf96** | DEG | DEG | DEG |
| **C12orf39** |  | DEG | DEG |
| **C15orf65** | DEG | DEG | DEG |
| **C17orf103** | DEG | DEG |  |
| **C19orf38** |  |  | DEG |
| **C1orf106** | DEG |  | DEG |
| **C1orf110** |  |  | DEG |
| **C1orf115** | DEG | DEG | DEG |
| **C1orf134** | DEG |  |  |
| **C1orf147** | DEG |  | DEG |
| **C1orf195** | DEG |  | DEG |
| **C1orf204** | DEG |  |  |
| **C1orf228** | DEG |  |  |
| **C1orf54** |  |  | DEG |
| **C1QL4** | DEG | DEG | DEG |
| **C1QTNF1** | DEG |  | DEG |
| **C1QTNF1-AS1** |  |  | DEG |
| **C1R** | DEG | DEG |  |
| **C1RL-AS1** | DEG |  |  |
| **C1S** | DEG | DEG |  |
| **C2** | DEG |  |  |
| **C20orf141** | DEG | DEG |  |
| **C2CD2** |  |  | DEG |
| **C3** | DEG |  | DEG |
| **C3AR1** | DEG |  |  |
| **C3orf58** |  | DEG |  |
| **C4B** |  |  | DEG |
| **C5** | DEG | DEG | DEG |
| **C5orf56** | DEG | DEG |  |
| **C8orf34** | DEG | DEG |  |
| **C8orf46** | DEG | DEG | DEG |
| **C9orf131** | DEG | DEG | DEG |
| **C9orf47** | DEG |  | DEG |
| **C9orf9** | DEG | DEG |  |
| **CACNA1I** | DEG |  |  |
| **CACNG4** | DEG |  |  |
| **CADM1** | DEG |  |  |
| **CADPS2** | DEG | DEG |  |
| **CALCRL** | DEG |  | DEG |
| **CAMK1G** | DEG |  | DEG |
| **CAPN12** | DEG |  | DEG |
| **CARF** | DEG |  |  |
| **CASC10** |  |  | DEG |
| **CASC7** |  |  | DEG |
| **CASP4** | DEG |  |  |
| **CASP7** | DEG |  |  |
| **CAV1** | DEG |  | DEG |
| **CBS** | DEG | DEG | DEG |
| **CBX7** | DEG |  | DEG |
| **CCDC102B** |  |  | DEG |
| **CCDC113** | DEG | DEG |  |
| **CCDC158** |  |  | DEG |
| **CCDC3** | DEG |  |  |
| **CCDC64** | DEG |  |  |
| **CCDC64B** | DEG | DEG | DEG |
| **CCDC71L** | DEG |  |  |
| **CCDC85B** | DEG | DEG | DEG |
| **CCL13** | DEG |  |  |
| **CCL2** | DEG | DEG | DEG |
| **CCL5** | DEG |  |  |
| **CCL7** | DEG | DEG | DEG |
| **CCL8** | DEG |  |  |
| **CCND1** | DEG | DEG | DEG |
| **CCNL1** | DEG | DEG |  |
| **CCR1** |  | DEG |  |
| **CCRL2** | DEG |  |  |
| **CCRN4L** | DEG | DEG |  |
| **CD200** | DEG | DEG | DEG |
| **CD248** | DEG |  | DEG |
| **CD24P4** | DEG | DEG | DEG |
| **CD274** | DEG | DEG |  |
| **CD44** | DEG |  | DEG |
| **CD47** | DEG |  |  |
| **CD7** | DEG | DEG |  |
| **CD70** | DEG |  | DEG |
| **CD74** | DEG | DEG |  |
| **CD82** | DEG |  | DEG |
| **CD83** | DEG |  | DEG |
| **CDCP1** | DEG | DEG | DEG |
| **CDH1** |  |  | DEG |
| **CDK6** | DEG |  | DEG |
| **CDKL2** |  | DEG | DEG |
| **CEACAM1** | DEG | DEG |  |
| **CEBPD** |  |  | DEG |
| **CECR1** | DEG | DEG |  |
| **CELSR2** | DEG |  | DEG |
| **CETP** | DEG | DEG |  |
| **CFB** | DEG |  |  |
| **CFH** | DEG | DEG |  |
| **CFLAR** | DEG |  |  |
| **CGB7** | DEG | DEG | DEG |
| **CH25H** | DEG |  |  |
| **CHAC1** | DEG | DEG | DEG |
| **CHMP4C** |  | DEG | DEG |
| **CHRM4** | DEG |  |  |
| **CHRM5** |  |  | DEG |
| **CHRNA1** | DEG | DEG |  |
| **CHRNB2** | DEG | DEG |  |
| **CHST11** | DEG |  | DEG |
| **CHST15** | DEG | DEG | DEG |
| **CHSY3** | DEG |  |  |
| **CIITA** | DEG | DEG |  |
| **CITED2** | DEG |  |  |
| **CITF22-49D8,1** | DEG | DEG |  |
| **CITF22-92A6,1** | DEG |  | DEG |
| **CLCA2** |  |  | DEG |
| **CLCN4** | DEG |  |  |
| **CLDN1** | DEG |  | DEG |
| **CLDN14** |  | DEG | DEG |
| **CLDN5** | DEG | DEG | DEG |
| **CLEC2B** | DEG |  |  |
| **CLEC2D** | DEG |  | DEG |
| **CLIC2** | DEG | DEG |  |
| **CLMN** | DEG |  | DEG |
| **CLSTN2** | DEG | DEG | DEG |
| **CMAHP** | DEG | DEG | DEG |
| **CMPK2** | DEG | DEG |  |
| **CMTM4** | DEG |  |  |
| **CNTN5** | DEG |  |  |
| **COL11A1** | DEG |  | DEG |
| **COL17A1** | DEG | DEG | DEG |
| **COL21A1** | DEG | DEG | DEG |
| **COL27A1** | DEG | DEG | DEG |
| **COL4A4** | DEG |  | DEG |
| **COL5A1-AS1** | DEG | DEG | DEG |
| **COL5A3** | DEG |  | DEG |
| **COL7A1** | DEG | DEG | DEG |
| **COL9A2** |  |  | DEG |
| **COLGALT2** |  | DEG |  |
| **CORO6** | DEG |  | DEG |
| **CPA3** | DEG |  |  |
| **CPA4** |  | DEG |  |
| **CPEB2** | DEG |  |  |
| **CPT1A** | DEG | DEG |  |
| **CR1L** | DEG |  |  |
| **CRB2** | DEG |  | DEG |
| **CRISPLD2** | DEG | DEG | DEG |
| **CRMP1** |  |  | DEG |
| **CRYAB** | DEG | DEG | DEG |
| **CRYL1** |  |  | DEG |
| **CSF1** | DEG | DEG | DEG |
| **CSF2** | DEG |  | DEG |
| **CSRNP1** | DEG | DEG | DEG |
| **CSRNP3** | DEG | DEG | DEG |
| **CSTA** | DEG | DEG |  |
| **CTA-384D8,31** | DEG |  |  |
| **CTA-445C9,15** |  |  | DEG |
| **CTA-963H5,5** | DEG |  |  |
| **CTB-102L5,7** |  | DEG | DEG |
| **CTB-179K24,3** |  | DEG | DEG |
| **CTB-25B13,13** |  | DEG | DEG |
| **CTB-60B18,12** | DEG | DEG | DEG |
| **CTB-60B18,18** |  | DEG | DEG |
| **CTC-244M17,1** |  |  | DEG |
| **CTC-296K1,4** | DEG | DEG | DEG |
| **CTC-308K20,1** | DEG |  | DEG |
| **CTC-459F4,1** | DEG |  |  |
| **CTC-479C5,12** | DEG | DEG |  |
| **CTD-2023M8,1** | DEG |  |  |
| **CTD-2031P19,3** | DEG | DEG | DEG |
| **CTD-2036J7,1** |  |  | DEG |
| **CTD-2105E13,13** | DEG | DEG | DEG |
| **CTD-2196E14,5** |  |  | DEG |
| **CTD-2240E14,4** | DEG | DEG |  |
| **CTD-2260A17,1** | DEG | DEG |  |
| **CTD-2260A17,3** | DEG |  |  |
| **CTD-2319I12,1** |  | DEG |  |
| **CTD-2521M24,9** | DEG | DEG |  |
| **CTD-2541M15,1** | DEG |  |  |
| **CTD-2541M15,4** |  | DEG | DEG |
| **CTD-2547L24,3** | DEG |  |  |
| **CTD-2552K11,2** | DEG |  |  |
| **CTD-2587H19,3** |  |  | DEG |
| **CTD-2587H24,5** | DEG |  |  |
| **CTD-3035D6,2** |  |  | DEG |
| **CTD-3247F14,2** | DEG | DEG | DEG |
| **CTD-3252C9,2** |  |  | DEG |
| **CTF1** | DEG |  |  |
| **CTGF** | DEG |  | DEG |
| **CTH** | DEG | DEG | DEG |
| **CTSS** | DEG | DEG | DEG |
| **CUBN** | DEG | DEG |  |
| **CX3CL1** | DEG |  |  |
| **CXADR** | DEG |  | DEG |
| **CXCL1** | DEG | DEG | DEG |
| **CXCL10** | DEG | DEG |  |
| **CXCL11** | DEG | DEG |  |
| **CXCL12** | DEG | DEG | DEG |
| **CXCL2** | DEG | DEG | DEG |
| **CXCL3** | DEG |  | DEG |
| **CXCL5** | DEG |  | DEG |
| **CXCL6** | DEG |  |  |
| **CXCL9** | DEG | DEG |  |
| **CYFIP2** | DEG |  |  |
| **CYP21A2** | DEG |  |  |
| **CYP26B1** | DEG |  |  |
| **CYP27C1** | DEG |  |  |
| **CYP2A6** | DEG |  |  |
| **CYP2S1** | DEG |  |  |
| **CYP2U1** |  |  | DEG |
| **CYS1** | DEG | DEG | DEG |
| **DAAM2** | DEG | DEG |  |
| **DAB2IP** |  |  | DEG |
| **DACT1** | DEG |  | DEG |
| **DACT3-AS1** |  | DEG |  |
| **DBNDD1** |  |  | DEG |
| **DBP** | DEG | DEG | DEG |
| **DDC8** | DEG | DEG | DEG |
| **DDIT4** | DEG | DEG |  |
| **DDX58** | DEG | DEG | DEG |
| **DDX60** | DEG | DEG |  |
| **DDX60L** | DEG | DEG |  |
| **DEC1** | DEG |  | DEG |
| **DENND2A** | DEG | DEG | DEG |
| **DES** | DEG |  |  |
| **DEXI** | DEG | DEG |  |
| **DGKG** | DEG | DEG |  |
| **DGKI** | DEG | DEG | DEG |
| **DHRS13** |  | DEG |  |
| **DHRS3** | DEG | DEG | DEG |
| **DHX58** | DEG |  |  |
| **DIP2C** | DEG |  |  |
| **DLL1** | DEG |  |  |
| **DLX2** | DEG | DEG |  |
| **DMGDH** | DEG |  | DEG |
| **DNAH17** | DEG |  | DEG |
| **DNAH5** |  |  | DEG |
| **DNAJA4** | DEG |  |  |
| **DNAJC12** |  |  | DEG |
| **DNER** | DEG |  | DEG |
| **DNM3** | DEG |  |  |
| **DNPEP** | DEG | DEG |  |
| **DOCK10** | DEG | DEG |  |
| **DPP4** | DEG |  |  |
| **DPYSL3** | DEG | DEG |  |
| **DRAM1** | DEG |  | DEG |
| **DRP2** |  |  | DEG |
| **DSP** |  | DEG |  |
| **DTX3L** | DEG | DEG |  |
| **DUSP1** | DEG | DEG | DEG |
| **DUSP10** |  |  | DEG |
| **DUSP2** |  |  | DEG |
| **DUSP5** | DEG | DEG | DEG |
| **DUSP6** |  | DEG |  |
| **DUSP8** |  | DEG |  |
| **DYRK2** |  |  | DEG |
| **E2F3-IT1** |  |  | DEG |
| **EBI3** | DEG |  |  |
| **ECEL1P2** | DEG | DEG | DEG |
| **ECM2** | DEG | DEG | DEG |
| **ECT2L** |  |  | DEG |
| **EDA2R** | DEG |  | DEG |
| **EDAR** |  |  | DEG |
| **EDARADD** | DEG |  |  |
| **EDNRA** | DEG | DEG |  |
| **EFCAB12** | DEG | DEG |  |
| **EFHD1** | DEG | DEG | DEG |
| **EFHD2** | DEG |  | DEG |
| **EFNA1** | DEG |  | DEG |
| **EGFR** | DEG |  | DEG |
| **EGFR-AS1** |  |  | DEG |
| **EGR1** | DEG | DEG | DEG |
| **EGR2** | DEG | DEG | DEG |
| **EGR3** | DEG | DEG | DEG |
| **EHD1** | DEG |  | DEG |
| **EHHADH** | DEG | DEG | DEG |
| **EIF5AP3** |  |  | DEG |
| **ELK3** |  |  | DEG |
| **ELOVL7** | DEG |  | DEG |
| **EML1** | DEG | DEG |  |
| **EMP1** |  | DEG |  |
| **ENC1** | DEG | DEG |  |
| **EPHA1** |  |  | DEG |
| **EPHB2** |  | DEG |  |
| **EPOR** | DEG |  |  |
| **EPS8L2** | DEG |  | DEG |
| **EPSTI1** | DEG | DEG |  |
| **ERAP1** | DEG | DEG |  |
| **ERAP2** | DEG | DEG |  |
| **ERBB3** | DEG | DEG | DEG |
| **ERRFI1** | DEG | DEG |  |
| **ESM1** | DEG |  | DEG |
| **ESRRG** | DEG | DEG | DEG |
| **ETF1P2** | DEG |  | DEG |
| **ETS1** | DEG |  | DEG |
| **ETS2** | DEG |  | DEG |
| **ETV7** | DEG | DEG |  |
| **EVA1A** | DEG |  | DEG |
| **EVI2B** | DEG |  |  |
| **EXPH5** | DEG | DEG | DEG |
| **F2RL1** |  |  | DEG |
| **F2RL2** | DEG |  | DEG |
| **F2RL3** | DEG |  |  |
| **F3** | DEG | DEG |  |
| **FAIM3** | DEG |  |  |
| **FAM101A** | DEG |  | DEG |
| **FAM107A** | DEG | DEG |  |
| **FAM110B** |  |  | DEG |
| **FAM115C** | DEG | DEG |  |
| **FAM117A** | DEG |  |  |
| **FAM124A** | DEG | DEG | DEG |
| **FAM131C** | DEG |  |  |
| **FAM132B** |  | DEG |  |
| **FAM13C** | DEG |  | DEG |
| **FAM167A** | DEG | DEG | DEG |
| **FAM167B** | DEG |  |  |
| **FAM172A** | DEG | DEG |  |
| **FAM177B** | DEG |  | DEG |
| **FAM180A** | DEG | DEG | DEG |
| **FAM182B** | DEG |  |  |
| **FAM196B** |  |  | DEG |
| **FAM198B** | DEG | DEG | DEG |
| **FAM19A3** | DEG |  | DEG |
| **FAM20A** | DEG | DEG |  |
| **FAM20C** | DEG |  | DEG |
| **FAM211B** |  | DEG |  |
| **FAM213A** | DEG |  | DEG |
| **FAM217A** |  | DEG |  |
| **FAM222A** | DEG |  |  |
| **FAM229B** | DEG |  |  |
| **FAM26F** | DEG | DEG |  |
| **FAM43A** | DEG |  | DEG |
| **FAM46A** | DEG | DEG |  |
| **FAM46C** | DEG | DEG | DEG |
| **FAM57B** | DEG | DEG | DEG |
| **FAM63A** | DEG |  | DEG |
| **FAM65B** |  |  | DEG |
| **FAM65C** | DEG |  |  |
| **FAM78A** |  |  | DEG |
| **FAT3** | DEG |  |  |
| **FAXDC2** | DEG | DEG | DEG |
| **FBLN7** | DEG |  |  |
| **FBXL20** | DEG |  |  |
| **FBXO32** | DEG | DEG | DEG |
| **FBXO6** | DEG | DEG |  |
| **FCGR2A** | DEG | DEG |  |
| **FCHO1** |  |  | DEG |
| **FCRL6** | DEG |  |  |
| **FDCSP** | DEG |  | DEG |
| **FGF22** | DEG | DEG | DEG |
| **FGFR3** | DEG |  |  |
| **FHDC1** |  |  | DEG |
| **FHL1** | DEG |  | DEG |
| **FILIP1** | DEG |  | DEG |
| **FKBP1B** | DEG |  |  |
| **FLRT1** | DEG | DEG | DEG |
| **FLT1** | DEG | DEG |  |
| **FOS** | DEG | DEG | DEG |
| **FOSB** | DEG | DEG | DEG |
| **FOSL1** | DEG | DEG | DEG |
| **FOXC1** | DEG |  |  |
| **FOXC2** | DEG | DEG | DEG |
| **FOXE3** |  | DEG |  |
| **FOXO6** | DEG | DEG | DEG |
| **FOXP1-IT1** |  |  | DEG |
| **FOXQ1** |  | DEG | DEG |
| **FRMD6** |  |  | DEG |
| **FRMPD4** |  |  | DEG |
| **FRY** | DEG |  |  |
| **FST** | DEG | DEG |  |
| **FTH1P4** | DEG | DEG | DEG |
| **FUT1** |  | DEG | DEG |
| **FZD8** |  |  | DEG |
| **G0S2** | DEG |  | DEG |
| **GAB2** | DEG |  | DEG |
| **GALNT3** |  |  | DEG |
| **GAPDHP14** | DEG |  |  |
| **GATA3** | DEG |  |  |
| **GBP1** | DEG | DEG |  |
| **GBP1P1** | DEG | DEG |  |
| **GBP2** | DEG | DEG |  |
| **GBP3** | DEG | DEG |  |
| **GBP4** | DEG | DEG |  |
| **GBP5** | DEG | DEG |  |
| **GCA** |  |  | DEG |
| **GCH1** | DEG |  |  |
| **GDF15** | DEG | DEG | DEG |
| **GDNF** |  |  | DEG |
| **GDPD1** | DEG |  | DEG |
| **GDPD2** |  |  | DEG |
| **GDPD5** | DEG |  | DEG |
| **GEM** | DEG | DEG |  |
| **GFAP** |  |  | DEG |
| **GFPT2** | DEG |  | DEG |
| **GFRA1** | DEG | DEG |  |
| **GGT5** | DEG |  |  |
| **GIMAP2** | DEG | DEG |  |
| **GKAP1** |  | DEG |  |
| **GLRB** | DEG | DEG | DEG |
| **GLT8D2** | DEG |  |  |
| **GMPR** |  |  | DEG |
| **GNAT2** | DEG | DEG |  |
| **GOLGA6L20** | DEG |  |  |
| **GOLM1** | DEG | DEG |  |
| **GPM6A** |  |  | DEG |
| **GPR1** |  |  | DEG |
| **GPR115** | DEG | DEG | DEG |
| **GPR155** | DEG |  | DEG |
| **GPR158** | DEG | DEG |  |
| **GPR162** | DEG |  | DEG |
| **GPR39** | DEG | DEG |  |
| **GPR56** | DEG | DEG | DEG |
| **GPR68** | DEG | DEG | DEG |
| **GPR75** |  |  | DEG |
| **GPRC5B** | DEG |  | DEG |
| **GPT2** | DEG | DEG | DEG |
| **GPX3** | DEG |  |  |
| **GRAMD1C** |  |  | DEG |
| **GRAMD2** | DEG |  | DEG |
| **GREM1** | DEG | DEG | DEG |
| **GRIK1** |  | DEG | DEG |
| **GRIP2** | DEG |  |  |
| **GS1-358P8,4** | DEG |  |  |
| **GSAP** | DEG |  |  |
| **GSG1** |  |  | DEG |
| **GSTA4** | DEG |  |  |
| **GUCY1A2** | DEG |  |  |
| **GYG2** | DEG | DEG | DEG |
| **H1FX** |  | DEG |  |
| **HAPLN3** | DEG | DEG |  |
| **HAS2** | DEG | DEG | DEG |
| **HBEGF** | DEG |  | DEG |
| **HDAC9** | DEG |  | DEG |
| **HEATR5A** |  |  | DEG |
| **HECW2** | DEG |  | DEG |
| **HELZ2** | DEG |  |  |
| **HERC5** | DEG |  |  |
| **HERC6** | DEG | DEG |  |
| **HES1** | DEG | DEG | DEG |
| **HES6** | DEG | DEG |  |
| **HHIP-AS1** |  | DEG |  |
| **HHLA3** | DEG |  | DEG |
| **HIC1** | DEG | DEG | DEG |
| **HILS1** |  |  | DEG |
| **HIN1L** | DEG |  |  |
| **HIPK2** |  |  | DEG |
| **HIVEP1** | DEG |  | DEG |
| **HIVEP2** | DEG |  | DEG |
| **HIVEP3** |  |  | DEG |
| **HK2** | DEG | DEG |  |
| **HLA-A** | DEG |  |  |
| **HLA-B** | DEG | DEG |  |
| **HLA-C** | DEG | DEG |  |
| **HLA-DMA** |  | DEG |  |
| **HLA-DMB** |  | DEG |  |
| **HLA-DOB** | DEG |  |  |
| **HLA-DPA1** | DEG | DEG |  |
| **HLA-DRA** | DEG | DEG |  |
| **HLA-E** | DEG |  |  |
| **HLA-F** | DEG | DEG |  |
| **HLA-H** | DEG |  |  |
| **HMGA1** | DEG | DEG |  |
| **HMGA2** | DEG | DEG | DEG |
| **HNMT** |  |  | DEG |
| **HNRNPA1P25** |  |  | DEG |
| **HNRNPA1P33** | DEG | DEG | DEG |
| **HNRNPA1P68** |  | DEG | DEG |
| **HNRNPA3P7** |  |  | DEG |
| **HORMAD1** | DEG |  |  |
| **HOTAIR** | DEG |  |  |
| **HOXA6** | DEG |  |  |
| **HOXB9** | DEG | DEG |  |
| **HPSE** | DEG | DEG | DEG |
| **HRH1** | DEG |  | DEG |
| **HS3ST3A1** | DEG | DEG | DEG |
| **HS3ST3B1** | DEG |  | DEG |
| **HSD11B1** | DEG |  |  |
| **HSD3BP5** |  |  | DEG |
| **HSPA12A** |  |  | DEG |
| **HSPA6** |  | DEG |  |
| **HSPA8P11** | DEG | DEG |  |
| **HSPH1** | DEG |  |  |
| **HTR7** | DEG |  |  |
| **ICAM1** | DEG | DEG | DEG |
| **ICAM5** |  | DEG |  |
| **ICOSLG** | DEG |  | DEG |
| **ID1** | DEG | DEG | DEG |
| **ID2** | DEG | DEG | DEG |
| **IDI2-AS1** | DEG |  |  |
| **IDO1** | DEG | DEG |  |
| **IER2** |  | DEG | DEG |
| **IER3** | DEG |  | DEG |
| **IER5** |  | DEG | DEG |
| **IFI27** | DEG | DEG |  |
| **IFI30** | DEG |  |  |
| **IFI35** | DEG | DEG |  |
| **IFI44** | DEG | DEG |  |
| **IFI44L** | DEG | DEG |  |
| **IFI6** | DEG | DEG |  |
| **IFIH1** | DEG | DEG |  |
| **IFIT1** | DEG |  |  |
| **IFIT2** | DEG | DEG |  |
| **IFIT3** | DEG | DEG |  |
| **IFIT5** | DEG | DEG |  |
| **IFITM1** | DEG |  |  |
| **IFN+TNFAR2** | DEG |  |  |
| **IFN+TNFGR2** | DEG |  |  |
| **IGFBP5** | DEG |  |  |
| **IGKV1OR2-108** | DEG |  | DEG |
| **IGSF6** |  |  | DEG |
| **IKBKE** | DEG |  | DEG |
| **IL11** | DEG |  |  |
| **IL12A** | DEG |  |  |
| **IL12RB1** | DEG | DEG |  |
| **IL15** | DEG | DEG |  |
| **IL15RA** | DEG | DEG | DEG |
| **IL16** | DEG | DEG | DEG |
| **IL18BP** | DEG | DEG |  |
| **IL1A** | DEG |  | DEG |
| **IL1B** | DEG |  | DEG |
| **IL20RB** | DEG | DEG | DEG |
| **IL21R** | DEG | DEG |  |
| **IL24** | DEG |  |  |
| **IL31RA** | DEG |  |  |
| **IL32** | DEG | DEG | DEG |
| **IL33** | DEG |  |  |
| **IL34** | DEG |  | DEG |
| **IL3RA** | DEG |  |  |
| **IL4I1** | DEG |  |  |
| **IL4R** | DEG | DEG | DEG |
| **IL6** | DEG |  | DEG |
| **IL7** | DEG |  |  |
| **IL7R** | DEG | DEG | DEG |
| **IL8** | DEG |  | DEG |
| **IMPA2** | DEG |  | DEG |
| **IMPDH1P10** | DEG |  |  |
| **INADL** | DEG | DEG | DEG |
| **INHBA** | DEG |  | DEG |
| **INHBA-AS1** | DEG |  | DEG |
| **INHBE** | DEG | DEG | DEG |
| **INPP5D** | DEG | DEG | DEG |
| **INPP5J** | DEG | DEG | DEG |
| **INSIG1** |  | DEG |  |
| **IP6K3** | DEG | DEG | DEG |
| **IQCJ-SCHIP1-AS1** | DEG |  |  |
| **IRAK1BP1** | DEG |  |  |
| **IRAK2** | DEG |  | DEG |
| **IRF1** | DEG | DEG |  |
| **IRF2** | DEG |  |  |
| **IRF9** | DEG | DEG |  |
| **IRG1** | DEG |  |  |
| **IRS2** | DEG | DEG | DEG |
| **ISG15** | DEG | DEG |  |
| **ISG20** | DEG |  |  |
| **ITGA2** | DEG | DEG | DEG |
| **ITGB4** | DEG | DEG | DEG |
| **ITGB6** | DEG |  | DEG |
| **ITIH5** |  | DEG |  |
| **ITK** | DEG | DEG |  |
| **ITPRIPL2** | DEG |  |  |
| **JAG1** |  |  | DEG |
| **JAG2** | DEG | DEG |  |
| **JAK2** | DEG |  |  |
| **JDP2** | DEG |  | DEG |
| **JMY** | DEG | DEG | DEG |
| **JUNB** |  | DEG |  |
| **KAL1** | DEG |  |  |
| **KANK1** | DEG |  |  |
| **KB-1732A1,1** | DEG | DEG | DEG |
| **KCNA7** | DEG | DEG | DEG |
| **KCNAB2** | DEG |  |  |
| **KCNE1** |  |  | DEG |
| **KCNE3** |  |  | DEG |
| **KCNE4** |  |  | DEG |
| **KCNG1** |  | DEG |  |
| **KCNH3** | DEG |  |  |
| **KCNH7** | DEG | DEG | DEG |
| **KCNJ15** | DEG | DEG | DEG |
| **KCNMB4** | DEG |  |  |
| **KCNN4** |  | DEG |  |
| **KCNQ5** | DEG | DEG |  |
| **KCNQ5-IT1** |  | DEG |  |
| **KCNT2** |  |  | DEG |
| **KCTD12** | DEG | DEG |  |
| **KCTD16** | DEG | DEG | DEG |
| **KCTD19** | DEG |  |  |
| **KDM6B** | DEG |  |  |
| **KDR** | DEG | DEG |  |
| **KIAA0247** | DEG |  |  |
| **KIAA0319** | DEG |  |  |
| **KIAA1161** | DEG |  |  |
| **KIAA1199** | DEG |  | DEG |
| **KIAA1211** | DEG | DEG | DEG |
| **KIAA1217** | DEG | DEG |  |
| **KIAA1467** | DEG |  | DEG |
| **KIAA1644** | DEG | DEG | DEG |
| **KIAA1683** | DEG |  | DEG |
| **KIAA1755** | DEG | DEG |  |
| **KIT** | DEG | DEG | DEG |
| **KLF10** | DEG | DEG | DEG |
| **KLF17** | DEG |  | DEG |
| **KLF2** | DEG | DEG | DEG |
| **KLF4** | DEG | DEG | DEG |
| **KLF5** | DEG | DEG |  |
| **KLF9** | DEG | DEG | DEG |
| **KLHDC7B** | DEG |  | DEG |
| **KLHL15** | DEG | DEG | DEG |
| **KLHL24** | DEG | DEG | DEG |
| **KLHL4** | DEG |  |  |
| **KLHL7-AS1** |  | DEG | DEG |
| **KRT121P** | DEG |  | DEG |
| **KRT34** | DEG |  |  |
| **KRT7** |  |  | DEG |
| **KRT8P42** | DEG |  | DEG |
| **KRTAP1-5** |  |  | DEG |
| **KRTAP2-3** |  |  | DEG |
| **KYNU** | DEG |  |  |
| **LACTB** | DEG |  |  |
| **LAG3** | DEG |  |  |
| **LAMB3** | DEG | DEG | DEG |
| **LAMC2** | DEG | DEG | DEG |
| **LAP3** | DEG | DEG |  |
| **LCA5** | DEG |  |  |
| **LGALS17A** | DEG | DEG |  |
| **LGALS9** | DEG | DEG | DEG |
| **LHFPL2** | DEG |  | DEG |
| **LIF** | DEG |  | DEG |
| **LIMD2** | DEG |  |  |
| **LIMS2** |  |  | DEG |
| **LIN7B** | DEG |  | DEG |
| **LINC00152** | DEG | DEG |  |
| **LINC00263** | DEG | DEG | DEG |
| **LINC00312** | DEG | DEG | DEG |
| **LINC00341** |  |  | DEG |
| **LINC00471** |  |  | DEG |
| **LINC00511** | DEG | DEG |  |
| **LINC00525** | DEG |  |  |
| **LINC00619** | DEG | DEG |  |
| **LINC00622** |  |  | DEG |
| **LINC00669** | DEG | DEG |  |
| **LINC00702** | DEG | DEG | DEG |
| **LINC00704** |  | DEG |  |
| **LINC00707** |  | DEG |  |
| **LINC00842** | DEG | DEG | DEG |
| **LINC00856** | DEG |  |  |
| **LINC00862** | DEG | DEG |  |
| **LINC00886** |  |  | DEG |
| **LINC00899** | DEG |  |  |
| **LINC00941** | DEG | DEG | DEG |
| **LINC00942** | DEG |  | DEG |
| **LINC00973** | DEG |  | DEG |
| **LINC01010** | DEG | DEG |  |
| **LINC01137** | DEG |  |  |
| **LINC01152** |  |  | DEG |
| **LIPG** | DEG |  |  |
| **LMCD1** |  |  | DEG |
| **LMOD1** | DEG | DEG | DEG |
| **LMOD2** | DEG |  |  |
| **LOH12CR2** | DEG |  | DEG |
| **LOX** | DEG |  |  |
| **LPHN2** |  |  | DEG |
| **LPXN** | DEG |  | DEG |
| **LRIG1** |  |  | DEG |
| **LRRC15** | DEG | DEG | DEG |
| **LRRC17** |  |  | DEG |
| **LRRC20** | DEG |  | DEG |
| **LRRC32** | DEG | DEG | DEG |
| **LRRC37A16P** | DEG |  |  |
| **LRRC37A6P** | DEG |  | DEG |
| **LRRC37A9P** |  |  | DEG |
| **LRRC4** |  |  | DEG |
| **LRRC63** | DEG | DEG |  |
| **LRRC7** | DEG |  |  |
| **LRRN3** | DEG |  | DEG |
| **LRRN4CL** | DEG |  | DEG |
| **LRRTM2** | DEG | DEG |  |
| **LSAMP** | DEG | DEG | DEG |
| **LSMEM2** | DEG |  |  |
| **LUCAT1** | DEG |  |  |
| **LURAP1L** | DEG | DEG | DEG |
| **LY6K** |  |  | DEG |
| **LYN** | DEG |  |  |
| **LYPD5** | DEG |  |  |
| **LYPD6B** | DEG |  |  |
| **LZTS1** |  |  | DEG |
| **LZTS3** | DEG | DEG | DEG |
| **MAFB** | DEG | DEG |  |
| **MAFF** | DEG | DEG | DEG |
| **MAMSTR** | DEG |  | DEG |
| **MAN1C1** | DEG | DEG | DEG |
| **MANSC1** | DEG |  |  |
| **MAOB** |  |  | DEG |
| **MAP1A** |  |  | DEG |
| **MAP2K3** | DEG | DEG | DEG |
| **MAP2K6** | DEG |  | DEG |
| **MAP3K14** |  | DEG |  |
| **MAP3K5** | DEG |  | DEG |
| **MAP3K8** | DEG | DEG | DEG |
| **MAPK10** | DEG |  |  |
| **MARCH10** |  | DEG | DEG |
| **MARCH3** |  |  | DEG |
| **MBP** |  |  | DEG |
| **MCAM** | DEG |  |  |
| **MDGA1** | DEG | DEG |  |
| **MDH1B** | DEG |  | DEG |
| **MEDAG** |  | DEG |  |
| **MEFV** | DEG |  | DEG |
| **MEOX1** | DEG |  | DEG |
| **METTL1** | DEG |  | DEG |
| **METTL7A** | DEG | DEG | DEG |
| **METTL7B** | DEG |  | DEG |
| **MFHAS1** | DEG |  |  |
| **MFSD2A** | DEG |  | DEG |
| **MGARP** | DEG | DEG | DEG |
| **MGAT3** | DEG |  | DEG |
| **MGLL** | DEG |  | DEG |
| **MID1IP1** | DEG | DEG | DEG |
| **MILR1** |  |  | DEG |
| **MIR1262** |  | DEG |  |
| **MIR145** | DEG | DEG | DEG |
| **MIR146A** | DEG |  | DEG |
| **MIR155HG** | DEG | DEG | DEG |
| **MIR17HG** | DEG | DEG |  |
| **MIR194-2** | DEG | DEG |  |
| **MIR22HG** | DEG | DEG | DEG |
| **MIR29A** | DEG | DEG | DEG |
| **MIR4482-1** | DEG | DEG | DEG |
| **MIR503HG** |  | DEG |  |
| **MIR600HG** | DEG |  |  |
| **MISP** | DEG | DEG | DEG |
| **MITF** | DEG |  | DEG |
| **MKX** | DEG | DEG | DEG |
| **MLKL** | DEG | DEG |  |
| **MMP1** | DEG |  |  |
| **MMP11** | DEG |  |  |
| **MMP13** | DEG |  |  |
| **MMP2** | DEG |  | DEG |
| **MMP25** | DEG | DEG |  |
| **MMP3** |  |  | DEG |
| **MMP9** | DEG |  | DEG |
| **MOAP1** | DEG |  |  |
| **MOCOS** |  |  | DEG |
| **MOK** |  |  | DEG |
| **MORF4L1P4** | DEG |  |  |
| **MORN4** | DEG | DEG | DEG |
| **MOV10L1** | DEG |  | DEG |
| **MPP7** | DEG |  |  |
| **MPRIP-AS1** |  |  | DEG |
| **MR1** |  |  | DEG |
| **MROH8** | DEG |  |  |
| **MRPL53** |  | DEG |  |
| **MRPS24** | DEG |  | DEG |
| **MRVI1** | DEG |  |  |
| **MSANTD1** | DEG | DEG |  |
| **MSC** | DEG |  | DEG |
| **MSI1** |  |  | DEG |
| **MSX1** | DEG |  |  |
| **MT1E** | DEG |  |  |
| **MT1X** | DEG |  | DEG |
| **MT2A** | DEG | DEG | DEG |
| **MT2P1** | DEG | DEG | DEG |
| **MTCYBP3** | DEG | DEG | DEG |
| **MTND1P11** | DEG |  |  |
| **MTND5P14** | DEG |  |  |
| **MT-RNR1** | DEG |  |  |
| **MT-RNR2** | DEG |  |  |
| **MTSS1** | DEG |  | DEG |
| **MTURN** | DEG | DEG |  |
| **MUC12** | DEG |  |  |
| **MUC17** | DEG |  |  |
| **MVK** | DEG |  | DEG |
| **MVP** | DEG |  |  |
| **MX1** | DEG | DEG |  |
| **MX2** | DEG | DEG |  |
| **MXD3** | DEG | DEG | DEG |
| **MXRA5** |  | DEG |  |
| **MYC** | DEG | DEG | DEG |
| **MYCL** | DEG |  |  |
| **MYEF2** | DEG |  |  |
| **MYEOV** | DEG | DEG | DEG |
| **MYH15** |  | DEG | DEG |
| **MYL1** | DEG |  |  |
| **MYLIP** |  | DEG |  |
| **MYLK2** | DEG | DEG | DEG |
| **MYLK3** | DEG |  | DEG |
| **MYO10** | DEG |  | DEG |
| **MYO16** | DEG |  |  |
| **MYO18A** | DEG |  | DEG |
| **MYOCD** |  |  | DEG |
| **MYPN** | DEG | DEG | DEG |
| **NABP1** | DEG | DEG |  |
| **NAMPT** | DEG |  |  |
| **NAV2** | DEG | DEG | DEG |
| **NAV3** | DEG |  | DEG |
| **NBEAL2** | DEG |  | DEG |
| **NCAM1** | DEG |  | DEG |
| **NDNF** | DEG | DEG |  |
| **NDP** |  |  | DEG |
| **NDUFA4L2** | DEG | DEG | DEG |
| **NEDD9** | DEG |  | DEG |
| **NES** | DEG |  |  |
| **NFATC2** | DEG | DEG | DEG |
| **NFE2** | DEG | DEG | DEG |
| **NFE2L3** | DEG |  |  |
| **NFIA** | DEG |  |  |
| **NFKB1** | DEG |  | DEG |
| **NFKB2** | DEG |  | DEG |
| **NFKBIA** | DEG |  | DEG |
| **NFKBIE** | DEG |  | DEG |
| **NGEF** | DEG | DEG |  |
| **NGF** | DEG |  |  |
| **NHSL2** | DEG |  | DEG |
| **NICN1** | DEG |  |  |
| **NINJ1** | DEG |  | DEG |
| **NKX3-1** | DEG | DEG | DEG |
| **NLGN1** | DEG |  | DEG |
| **NLRC5** | DEG | DEG |  |
| **NLRP10** | DEG | DEG |  |
| **NLRP3** | DEG | DEG | DEG |
| **NMBR** | DEG |  |  |
| **NMI** | DEG | DEG |  |
| **NOV** |  | DEG | DEG |
| **NPAS2** | DEG |  | DEG |
| **NPIPB11** |  |  | DEG |
| **NPR3** |  |  | DEG |
| **NPTX1** | DEG | DEG | DEG |
| **NPY4R** | DEG |  |  |
| **NR1D1** | DEG | DEG | DEG |
| **NR4A1** | DEG | DEG | DEG |
| **NR4A2** | DEG | DEG | DEG |
| **NR4A3** | DEG | DEG | DEG |
| **NRBF2P5** |  |  | DEG |
| **NRBP2** | DEG |  | DEG |
| **NRCAM** | DEG | DEG | DEG |
| **NREP** | DEG | DEG |  |
| **NRG1** | DEG |  |  |
| **NRP1** | DEG | DEG |  |
| **NRP2** | DEG |  | DEG |
| **NSUN7** | DEG | DEG | DEG |
| **NT5E** | DEG | DEG |  |
| **NT5M** | DEG |  |  |
| **NTM** | DEG |  | DEG |
| **NTN1** | DEG |  |  |
| **NTRK3** | DEG |  |  |
| **NUAK2** | DEG |  | DEG |
| **NUB1** | DEG |  |  |
| **NUBP1** | DEG |  |  |
| **NUPR1** | DEG | DEG | DEG |
| **NYAP1** | DEG | DEG |  |
| **NYNRIN** | DEG | DEG | DEG |
| **OAF** | DEG |  |  |
| **OAS1** | DEG | DEG |  |
| **OAS2** | DEG | DEG |  |
| **OAS3** | DEG | DEG |  |
| **OASL** | DEG | DEG |  |
| **OBSL1** | DEG |  |  |
| **OCLN** | DEG | DEG | DEG |
| **ODF3B** | DEG | DEG |  |
| **OGFR** | DEG |  |  |
| **OLFM2** | DEG |  |  |
| **OLR1** | DEG | DEG | DEG |
| **OPLAH** |  |  | DEG |
| **OPRL1** | DEG |  |  |
| **OR2I1P** | DEG |  |  |
| **ORAI3** |  |  | DEG |
| **OSCAR** |  |  | DEG |
| **OSGIN1** | DEG |  |  |
| **OSR2** | DEG | DEG | DEG |
| **OTOGL** | DEG | DEG |  |
| **OTUD4** | DEG |  |  |
| **OXER1** |  | DEG | DEG |
| **P2RX6** | DEG |  | DEG |
| **P2RX7** |  |  | DEG |
| **PAMR1** |  |  | DEG |
| **PANX1** | DEG | DEG | DEG |
| **PANX2** | DEG |  |  |
| **PAPPA** | DEG |  | DEG |
| **PAPPA2** | DEG | DEG | DEG |
| **PAQR5** | DEG |  | DEG |
| **PAQR9** | DEG | DEG |  |
| **PARD3B** | DEG | DEG | DEG |
| **PARD6A** | DEG | DEG | DEG |
| **PARD6B** | DEG |  |  |
| **PARP12** | DEG | DEG |  |
| **PARP14** | DEG | DEG |  |
| **PARP9** | DEG | DEG |  |
| **PAX5** | DEG |  |  |
| **PAX8** | DEG | DEG | DEG |
| **PCDH18** |  |  | DEG |
| **PCDH7** | DEG | DEG |  |
| **PCK2** | DEG | DEG | DEG |
| **PCLO** |  |  | DEG |
| **PCP4L1** |  |  | DEG |
| **PCSK9** | DEG | DEG | DEG |
| **PDCD1LG2** | DEG | DEG |  |
| **PDE1A** | DEG | DEG | DEG |
| **PDE1C** | DEG |  |  |
| **PDE2A** | DEG |  |  |
| **PDGFA** | DEG | DEG | DEG |
| **PDLIM4** | DEG |  | DEG |
| **PDP2** | DEG |  |  |
| **PDZD7** | DEG |  | DEG |
| **PER1** |  | DEG |  |
| **PER2** | DEG |  |  |
| **PER3** | DEG |  |  |
| **PEX5L** |  | DEG |  |
| **PFKFB4** | DEG |  | DEG |
| **PGAM1P7** | DEG | DEG | DEG |
| **PGPEP1** | DEG | DEG | DEG |
| **PHACTR1** | DEG | DEG | DEG |
| **PHF11** | DEG | DEG |  |
| **PHF7** | DEG |  |  |
| **PHLDA1** | DEG |  |  |
| **PHOSPHO2** | DEG |  |  |
| **PIANP** | DEG |  |  |
| **PID1** | DEG |  | DEG |
| **PIGZ** | DEG | DEG |  |
| **PIK3AP1** | DEG | DEG |  |
| **PIK3C2B** | DEG |  | DEG |
| **PIK3CD** | DEG |  | DEG |
| **PILRA** | DEG |  |  |
| **PIM1** | DEG | DEG |  |
| **PITRM1-AS1** | DEG |  |  |
| **PIWIL2** |  |  | DEG |
| **PKP2** | DEG | DEG | DEG |
| **PLA2G4A** | DEG |  |  |
| **PLAC8** | DEG |  | DEG |
| **PLAU** | DEG |  | DEG |
| **PLAUR** | DEG | DEG | DEG |
| **PLCE1-AS1** | DEG | DEG | DEG |
| **PLEKHA6** | DEG | DEG | DEG |
| **PLEKHB1** | DEG |  |  |
| **PLEKHF1** |  |  | DEG |
| **PLEKHG6** | DEG |  |  |
| **PLEKHH2** | DEG |  |  |
| **PLEKHN1** | DEG |  |  |
| **PLEKHS1** |  | DEG |  |
| **PLK2** |  |  | DEG |
| **PLSCR1** | DEG |  |  |
| **PLSCR4** |  |  | DEG |
| **PLXNB3** | DEG |  |  |
| **PMAIP1** | DEG |  |  |
| **PMEPA1** |  |  | DEG |
| **PML** | DEG | DEG |  |
| **PODN** | DEG |  |  |
| **PODXL** | DEG | DEG | DEG |
| **POU2F2** | DEG |  | DEG |
| **PPA1** | DEG |  |  |
| **PPAP2B** | DEG |  | DEG |
| **PPAPDC1A** |  | DEG |  |
| **PPAPDC3** | DEG |  |  |
| **PPARGC1A** | DEG | DEG | DEG |
| **PPFIA4** | DEG |  |  |
| **PPIAP30** |  |  | DEG |
| **PPIF** | DEG |  | DEG |
| **PPM1E** | DEG | DEG | DEG |
| **PPP1R18** | DEG |  |  |
| **PPP1R9A** | DEG |  | DEG |
| **PPP3CC** | DEG |  | DEG |
| **PPP4R4** | DEG |  | DEG |
| **PRDM1** | DEG | DEG |  |
| **PRDM6** | DEG | DEG |  |
| **PRDM8** | DEG |  | DEG |
| **PRELP** | DEG |  | DEG |
| **PRICKLE1** | DEG | DEG | DEG |
| **PRKAA2** | DEG |  |  |
| **PRKCG** | DEG | DEG |  |
| **PRKCH** | DEG |  |  |
| **PRLR** |  |  | DEG |
| **PROZ** | DEG | DEG |  |
| **PRRG4** | DEG |  |  |
| **PRRT3-AS1** |  | DEG |  |
| **PRRT4** | DEG | DEG | DEG |
| **PSAT1** | DEG | DEG | DEG |
| **PSG5** | DEG |  |  |
| **PSMA2** | DEG |  |  |
| **PSMA6** | DEG |  |  |
| **PSMB10** | DEG | DEG |  |
| **PSMB8** | DEG | DEG |  |
| **PSMB9** | DEG | DEG |  |
| **PSME2** | DEG | DEG |  |
| **PTAFR** | DEG |  |  |
| **PTCH1** | DEG |  |  |
| **PTGER4** | DEG | DEG | DEG |
| **PTGES** | DEG |  |  |
| **PTGES3P1** | DEG | DEG |  |
| **PTGIS** |  |  | DEG |
| **PTGS1** |  | DEG |  |
| **PTGS2** |  | DEG | DEG |
| **PTHLH** | DEG | DEG | DEG |
| **PTK2B** | DEG |  | DEG |
| **PTPN13** | DEG |  | DEG |
| **PTPN22** | DEG |  |  |
| **PTPRC** | DEG |  |  |
| **PTPRE** | DEG |  | DEG |
| **PTPRK** | DEG |  | DEG |
| **PTPRN** | DEG |  | DEG |
| **PTPRQ** | DEG |  | DEG |
| **PTPRR** | DEG |  | DEG |
| **PTPRU** |  |  | DEG |
| **PTX3** | DEG |  | DEG |
| **PURG** | DEG |  |  |
| **PWAR6** | DEG |  |  |
| **PYGM** | DEG | DEG | DEG |
| **QPRT** | DEG |  | DEG |
| **RAB11FIP1** | DEG |  | DEG |
| **RAB26** |  | DEG | DEG |
| **RAB27B** | DEG | DEG | DEG |
| **RAB33A** | DEG |  | DEG |
| **RAB3A** | DEG |  | DEG |
| **RAB40B** | DEG |  |  |
| **RAD17P2** |  |  | DEG |
| **RALA** | DEG |  |  |
| **RALGPS1** | DEG |  |  |
| **RANBP3L** |  | DEG |  |
| **RARRES1** | DEG |  |  |
| **RARRES3** | DEG | DEG |  |
| **RASA3** |  |  | DEG |
| **RASGRP3** |  |  | DEG |
| **RASSF2** | DEG |  |  |
| **RASSF7** | DEG |  | DEG |
| **RASSF9** |  |  | DEG |
| **RBM24** |  | DEG |  |
| **RBM47** | DEG |  |  |
| **RBMS1** | DEG |  | DEG |
| **RBP7** | DEG |  | DEG |
| **RCAN2** | DEG |  | DEG |
| **RDH8** | DEG |  | DEG |
| **REC8** | DEG |  |  |
| **REEP1** | DEG |  |  |
| **REEP2** |  |  | DEG |
| **REEP6** |  |  | DEG |
| **RELB** | DEG |  | DEG |
| **RELN** | DEG | DEG | DEG |
| **RFX8** | DEG | DEG | DEG |
| **RFXAP** | DEG |  | DEG |
| **RGCC** | DEG |  | DEG |
| **RGMB-AS1** |  |  | DEG |
| **RGS20** | DEG |  | DEG |
| **RGS22** | DEG |  |  |
| **RGS9** | DEG | DEG |  |
| **RHBDF2** | DEG |  |  |
| **RHOB** | DEG |  |  |
| **RHPN1** | DEG | DEG |  |
| **RIMS3** | DEG | DEG | DEG |
| **RIPK2** | DEG | DEG |  |
| **RIPK4** |  |  | DEG |
| **RLTPR** | DEG |  |  |
| **RMI2** |  |  | DEG |
| **RN7SKP16** |  |  | DEG |
| **RN7SL368P** |  |  | DEG |
| **RN7SL834P** | DEG | DEG |  |
| **RNA18S5** | DEG |  |  |
| **RNA28S5** | DEG |  |  |
| **RND1** | DEG | DEG |  |
| **RND3** | DEG | DEG | DEG |
| **RNF157** | DEG |  |  |
| **RNF19B** | DEG | DEG | DEG |
| **RNFT2** | DEG |  | DEG |
| **RNU6-301P** |  |  | DEG |
| **RNU6-476P** |  |  | DEG |
| **RNU6-767P** |  | DEG | DEG |
| **RNU6ATAC7P** |  |  | DEG |
| **RNU7-124P** | DEG |  |  |
| **RNU7-40P** | DEG |  |  |
| **RNVU1-6** | DEG |  |  |
| **ROBO4** | DEG |  | DEG |
| **ROR1** | DEG |  |  |
| **RORA** | DEG |  |  |
| **ROS1** | DEG |  | DEG |
| **RP11-1002K11,1** | DEG |  |  |
| **RP11-101O6,2** |  |  | DEG |
| **RP11-1024P17,1** |  |  | DEG |
| **RP11-1055B8,7** | DEG | DEG | DEG |
| **RP11-1057B6,1** |  |  | DEG |
| **RP11-1060J15,3** |  |  | DEG |
| **RP11-114F10,3** | DEG |  |  |
| **RP11-119F7,5** | DEG |  |  |
| **RP11-125B21,2** | DEG | DEG | DEG |
| **RP11-131L23,1** | DEG |  |  |
| **RP11-131L23,2** | DEG |  |  |
| **RP11-134L10,1** | DEG | DEG |  |
| **RP11-1399P15,1** | DEG |  |  |
| **RP11-142M10,2** | DEG |  | DEG |
| **RP11-148B18,3** | DEG |  |  |
| **RP11-150O12,1** | DEG | DEG | DEG |
| **RP11-154H12,2** | DEG | DEG |  |
| **RP11-154H23,3** | DEG | DEG | DEG |
| **RP11-157P1,4** |  |  | DEG |
| **RP11-160O5,1** | DEG | DEG | DEG |
| **RP11-165P7,1** | DEG |  | DEG |
| **RP11-168K11,3** |  |  | DEG |
| **RP11-16C1,3** |  |  | DEG |
| **RP11-16E23,3** | DEG |  |  |
| **RP11-16E23,4** | DEG |  |  |
| **RP11-181K3,4** |  |  | DEG |
| **RP11-184M15,1** |  | DEG | DEG |
| **RP11-18I14,10** |  |  | DEG |
| **RP11-1C8,6** |  |  | DEG |
| **RP11-210K20,2** |  |  | DEG |
| **RP11-211G3,2** |  | DEG |  |
| **RP11-212I21,4** |  |  | DEG |
| **RP11-221N13,3** |  |  | DEG |
| **RP11-227D13,2** |  |  | DEG |
| **RP11-229P13,25** |  |  | DEG |
| **RP11-22H5,2** | DEG | DEG | DEG |
| **RP11-22L13,1** | DEG | DEG |  |
| **RP11-230G5,2** | DEG |  | DEG |
| **RP11-245M24,1** | DEG | DEG | DEG |
| **RP11-24J23,2** | DEG |  | DEG |
| **RP11-274E7,2** | DEG |  |  |
| **RP11-276H19,1** | DEG | DEG | DEG |
| **RP11-284F21,10** | DEG | DEG | DEG |
| **RP11-284F21,7** | DEG |  | DEG |
| **RP11-284F21,9** | DEG | DEG | DEG |
| **RP11-286E11,1** | DEG |  | DEG |
| **RP11-288L9,4** | DEG | DEG |  |
| **RP11-28B23,1** | DEG |  |  |
| **RP11-290F5,1** | DEG |  |  |
| **RP11-290L1,4** |  |  | DEG |
| **RP11-307C12,11** |  |  | DEG |
| **RP11-313E19,2** | DEG | DEG | DEG |
| **RP11-318C2,1** |  |  | DEG |
| **RP11-320G24,1** |  | DEG |  |
| **RP11-322D14,1** | DEG | DEG |  |
| **RP11-326C3,11** | DEG |  |  |
| **RP11-326I11,3** |  | DEG |  |
| **RP11-327F22,2** | DEG | DEG |  |
| **RP11-333O1,1** | DEG |  |  |
| **RP11-336K24,12** |  | DEG |  |
| **RP11-338C15,3** | DEG |  |  |
| **RP11-340F14,5** |  |  | DEG |
| **RP11-344E13,3** |  |  | DEG |
| **RP11-352D3,2** | DEG |  | DEG |
| **RP11-353M9,1** |  |  | DEG |
| **RP11-356I2,4** | DEG |  | DEG |
| **RP11-356J5,12** | DEG | DEG | DEG |
| **RP11-357N13,2** |  |  | DEG |
| **RP11-358B23,1** |  | DEG |  |
| **RP11-359E10,1** | DEG |  | DEG |
| **RP11-359K18,4** | DEG |  | DEG |
| **RP11-366L20,2** | DEG | DEG | DEG |
| **RP11-366L20,3** |  |  | DEG |
| **RP11-366L5,1** |  |  | DEG |
| **RP11-375I20,6** | DEG | DEG | DEG |
| **RP11-382A20,1** |  |  | DEG |
| **RP11-383F6,1** | DEG | DEG |  |
| **RP11-383J24,1** | DEG | DEG | DEG |
| **RP11-390P2,4** | DEG | DEG | DEG |
| **RP11-396K3,1** | DEG |  |  |
| **RP11-3L8,3** | DEG | DEG | DEG |
| **RP11-400K9,4** | DEG | DEG |  |
| **RP11-404P21,3** | DEG |  |  |
| **RP11-404P21,5** | DEG |  |  |
| **RP11-41O4,2** |  | DEG |  |
| **RP11-420G6,4** |  |  | DEG |
| **RP11-421E14,2** | DEG |  |  |
| **RP11-429P3,5** |  | DEG | DEG |
| **RP11-437J19,1** | DEG |  |  |
| **RP11-438D8,2** |  | DEG | DEG |
| **RP11-438L19,1** |  |  | DEG |
| **RP11-443A13,5** | DEG |  | DEG |
| **RP11-445L6,3** |  |  | DEG |
| **RP11-448G15,3** | DEG | DEG | DEG |
| **RP11-44D5,1** |  |  | DEG |
| **RP11-44K6,2** | DEG |  |  |
| **RP11-44K6,4** | DEG | DEG |  |
| **RP11-462G12,1** | DEG | DEG | DEG |
| **RP11-462G12,2** | DEG | DEG | DEG |
| **RP11-462L8,1** |  |  | DEG |
| **RP11-468E2,4** | DEG | DEG |  |
| **RP11-473M20,5** | DEG | DEG | DEG |
| **RP11-473M20,7** | DEG |  |  |
| **RP11-479G22,8** |  |  | DEG |
| **RP11-47I22,2** | DEG | DEG | DEG |
| **RP11-480I12,7** | DEG |  |  |
| **RP11-483P21,3** |  |  | DEG |
| **RP11-484D2,3** |  |  | DEG |
| **RP11-486B10,4** | DEG | DEG | DEG |
| **RP11-495P10,1** | DEG |  |  |
| **RP11-495P10,5** | DEG | DEG | DEG |
| **RP11-495P10,8** | DEG | DEG | DEG |
| **RP11-495P10,9** | DEG | DEG | DEG |
| **RP11-499E18,1** | DEG |  |  |
| **RP11-4C20,3** | DEG | DEG | DEG |
| **RP11-504A18,1** |  |  | DEG |
| **RP11-506B6,6** | DEG |  |  |
| **RP11-508N22,12** | DEG |  |  |
| **RP11-512N21,3** | DEG |  |  |
| **RP11-517O13,1** |  |  | DEG |
| **RP11-519G16,3** | DEG |  |  |
| **RP11-527H14,2** |  |  | DEG |
| **RP11-527H14,3** | DEG |  |  |
| **RP11-531F16,4** | DEG |  |  |
| **RP11-532F6,3** | DEG |  |  |
| **RP11-536C5,7** |  |  | DEG |
| **RP11-540O11,6** |  |  | DEG |
| **RP11-543N12,1** |  | DEG | DEG |
| **RP11-552F3,10** |  |  | DEG |
| **RP11-552M11,4** | DEG |  | DEG |
| **RP11-553A10,1** | DEG |  | DEG |
| **RP11-557H15,3** | DEG |  |  |
| **RP11-558F24,4** | DEG | DEG | DEG |
| **RP11-582J16,4** |  |  | DEG |
| **RP11-58E21,3** |  | DEG | DEG |
| **RP11-58K22,4** | DEG |  | DEG |
| **RP11-594N15,3** |  |  | DEG |
| **RP11-619F23,2** |  | DEG | DEG |
| **RP11-61A14,3** | DEG |  |  |
| **RP11-61F12,1** |  |  | DEG |
| **RP11-620J15,2** |  |  | DEG |
| **RP11-627K11,3** |  | DEG |  |
| **RP11-630D6,5** | DEG |  |  |
| **RP11-631F7,1** |  |  | DEG |
| **RP11-635N19,1** |  | DEG |  |
| **RP11-638I2,10** | DEG | DEG |  |
| **RP11-638I2,9** | DEG | DEG |  |
| **RP11-662I13,2** |  |  | DEG |
| **RP11-670E13,3** | DEG | DEG |  |
| **RP11-680H20,1** | DEG |  | DEG |
| **RP11-680H20,2** | DEG |  | DEG |
| **RP11-688I9,4** |  |  | DEG |
| **RP11-690G19,3** | DEG |  |  |
| **RP11-694I15,7** | DEG |  |  |
| **RP11-723O4,9** | DEG |  |  |
| **RP11-723P16,3** |  |  | DEG |
| **RP11-730A19,9** | DEG |  |  |
| **RP11-737O24,3** | DEG |  |  |
| **RP11-739N20,3** | DEG |  | DEG |
| **RP11-745A24,1** | DEG | DEG | DEG |
| **RP11-757C15,4** |  |  | DEG |
| **RP11-758P17,3** | DEG | DEG | DEG |
| **RP11-75L1,2** | DEG |  | DEG |
| **RP11-760L24,1** |  |  | DEG |
| **RP11-762H8,2** |  |  | DEG |
| **RP11-766F14,2** | DEG | DEG | DEG |
| **RP11-78A19,4** | DEG |  | DEG |
| **RP11-79H23,3** | DEG | DEG | DEG |
| **RP11-807E13,2** | DEG |  |  |
| **RP11-81B10,2** |  |  | DEG |
| **RP11-861A13,4** | DEG |  | DEG |
| **RP11-863P13,3** | DEG | DEG | DEG |
| **RP11-865I6,2** | DEG |  |  |
| **RP11-873E20,1** |  |  | DEG |
| **RP11-875O11,1** | DEG |  | DEG |
| **RP11-89H19,1** | DEG |  |  |
| **RP11-90L20,2** |  | DEG | DEG |
| **RP1-191J18,66** |  | DEG |  |
| **RP11-91J19,4** | DEG | DEG |  |
| **RP11-91K9,1** | DEG | DEG | DEG |
| **RP1-193H18,2** | DEG | DEG | DEG |
| **RP11-95P2,1** |  | DEG | DEG |
| **RP11-981P6,1** | DEG |  | DEG |
| **RP11-98C1,1** |  |  | DEG |
| **RP1-199J3,5** | DEG |  |  |
| **RP13-297E16,4** | DEG |  |  |
| **RP1-32I10,10** |  |  | DEG |
| **RP13-631K18,5** |  |  | DEG |
| **RP1-39G22,7** | DEG |  |  |
| **RP1-50J22,4** | DEG | DEG |  |
| **RP1-68D18,2** | DEG |  | DEG |
| **RP1-68D18,4** | DEG | DEG | DEG |
| **RP1-74M1,3** |  | DEG |  |
| **RP1-86D1,4** |  |  | DEG |
| **RP1-86D1,5** |  |  | DEG |
| **RP1-93I3,1** | DEG |  |  |
| **RP3-391O22,1** |  |  | DEG |
| **RP3-416H24,1** | DEG |  | DEG |
| **RP3-437C15,1** | DEG |  | DEG |
| **RP3-467K16,4** | DEG |  |  |
| **RP3-471M13,2** | DEG |  |  |
| **RP3-476K8,4** |  |  | DEG |
| **RP3-508I15,19** | DEG |  |  |
| **RP3-508I15,9** | DEG |  |  |
| **RP3-512B11,3** |  |  | DEG |
| **RP4-537K23,4** |  |  | DEG |
| **RP4-539M6,20** |  |  | DEG |
| **RP4-555D20,2** | DEG | DEG | DEG |
| **RP4-564F22,5** | DEG | DEG | DEG |
| **RP4-569M23,4** |  | DEG |  |
| **RP4-657D16,3** | DEG | DEG | DEG |
| **RP4-663N10,2** | DEG |  |  |
| **RP4-779E11,3** | DEG |  | DEG |
| **RP4-794H19,1** |  | DEG |  |
| **RP4-794H19,4** | DEG | DEG |  |
| **RP5-1142A6,2** | DEG |  | DEG |
| **RP5-1142A6,8** | DEG |  | DEG |
| **RP5-1157M23,2** | DEG |  |  |
| **RP5-1198O20,4** |  |  | DEG |
| **RP5-902P8,10** | DEG |  |  |
| **RP5-916O11,3** | DEG |  |  |
| **RP6-109B7,2** |  |  | DEG |
| **RP6-99M1,2** | DEG |  | DEG |
| **RPL12P18** | DEG | DEG |  |
| **RPL21P121** | DEG |  | DEG |
| **RPL3P6** | DEG |  |  |
| **RPL7P26** |  |  | DEG |
| **RPS10P7** | DEG |  | DEG |
| **RPS20P15** |  |  | DEG |
| **RPS26P21** |  |  | DEG |
| **RPS6KA2** | DEG |  |  |
| **RPSAP52** | DEG | DEG | DEG |
| **RRAD** | DEG |  | DEG |
| **RSAD2** | DEG | DEG |  |
| **RTN4R** | DEG | DEG | DEG |
| **RTP4** | DEG | DEG |  |
| **S100A3** | DEG |  |  |
| **S1PR1** | DEG |  |  |
| **S1PR3** | DEG | DEG | DEG |
| **SALL2** | DEG |  |  |
| **SAMD12** | DEG |  |  |
| **SAMD3** | DEG |  |  |
| **SAMD4A** | DEG |  | DEG |
| **SAMD9** | DEG | DEG |  |
| **SAMD9L** | DEG | DEG |  |
| **SAMHD1** | DEG | DEG |  |
| **SBNO2** | DEG |  |  |
| **SCARF1** | DEG |  |  |
| **SCG5** | DEG |  | DEG |
| **SCHIP1** | DEG | DEG |  |
| **SCN2A** | DEG |  |  |
| **SCN3A** | DEG | DEG |  |
| **SCN9A** |  |  | DEG |
| **SCNN1A** |  |  | DEG |
| **SDC4** | DEG |  | DEG |
| **SDPR** | DEG | DEG | DEG |
| **SEC14L2** |  |  | DEG |
| **SECTM1** | DEG | DEG |  |
| **SEL1L3** | DEG | DEG | DEG |
| **SELPLG** | DEG | DEG | DEG |
| **SEMA3C** | DEG |  | DEG |
| **SEMA3E** |  |  | DEG |
| **SEMA3F** |  |  | DEG |
| **SEMA4A** | DEG |  |  |
| **SEMA4D** | DEG | DEG | DEG |
| **SEMA4G** | DEG |  | DEG |
| **SEMA6B** | DEG |  | DEG |
| **SEMA7A** | DEG | DEG | DEG |
| **SENP3-EIF4A1** | DEG |  |  |
| **SEPT3** | DEG |  |  |
| **SEPT4** |  |  | DEG |
| **SERPINA1** | DEG |  | DEG |
| **SERPINB2** | DEG | DEG | DEG |
| **SERPINB7** | DEG | DEG |  |
| **SERPINB8** | DEG | DEG | DEG |
| **SERPINE1** | DEG |  | DEG |
| **SERPINE2** | DEG |  | DEG |
| **SERPING1** | DEG | DEG |  |
| **SERTAD1** | DEG |  |  |
| **SESN2** | DEG | DEG | DEG |
| **SESN3** | DEG | DEG | DEG |
| **SFTA1P** | DEG |  | DEG |
| **SGCG** | DEG | DEG | DEG |
| **SGK1** | DEG | DEG | DEG |
| **SGK223** |  |  | DEG |
| **SH2B3** | DEG | DEG | DEG |
| **SH2D3C** | DEG | DEG | DEG |
| **SH3BGR** | DEG | DEG | DEG |
| **SHB** | DEG |  | DEG |
| **SHC4** | DEG | DEG |  |
| **SIAH3** | DEG |  | DEG |
| **SIGLEC15** |  |  | DEG |
| **SIK1** | DEG |  |  |
| **SIK3-IT1** | DEG |  |  |
| **SIM1** |  |  | DEG |
| **SLA** | DEG |  | DEG |
| **SLAMF8** | DEG | DEG |  |
| **SLC13A5** |  | DEG | DEG |
| **SLC15A3** | DEG | DEG |  |
| **SLC16A10** | DEG |  |  |
| **SLC16A12** |  | DEG | DEG |
| **SLC16A14** | DEG | DEG | DEG |
| **SLC16A5** | DEG |  | DEG |
| **SLC19A2** |  |  | DEG |
| **SLC1A2** | DEG |  |  |
| **SLC1A3** | DEG |  | DEG |
| **SLC1A4** | DEG | DEG | DEG |
| **SLC22A15** | DEG | DEG | DEG |
| **SLC22A23** | DEG |  | DEG |
| **SLC25A22** | DEG |  |  |
| **SLC25A25** | DEG | DEG | DEG |
| **SLC25A37** | DEG |  | DEG |
| **SLC25A42** | DEG |  | DEG |
| **SLC28A3** | DEG | DEG | DEG |
| **SLC29A2** | DEG |  |  |
| **SLC2A12** | DEG |  | DEG |
| **SLC2A5** | DEG |  |  |
| **SLC2A6** | DEG |  | DEG |
| **SLC35E4** | DEG | DEG | DEG |
| **SLC38A3** | DEG |  |  |
| **SLC38A4** | DEG |  | DEG |
| **SLC39A14** | DEG |  | DEG |
| **SLC3A1** |  |  | DEG |
| **SLC40A1** | DEG | DEG | DEG |
| **SLC41A2** | DEG |  | DEG |
| **SLC44A2** |  |  | DEG |
| **SLC45A1** |  |  | DEG |
| **SLC46A3** | DEG |  |  |
| **SLC48A1** | DEG |  | DEG |
| **SLC6A6** | DEG | DEG |  |
| **SLC7A11** | DEG | DEG | DEG |
| **SLC7A2** | DEG |  | DEG |
| **SLC7A4** | DEG |  | DEG |
| **SLC7A8** | DEG | DEG | DEG |
| **SLC8A2** |  | DEG |  |
| **SLC9A7P1** | DEG | DEG | DEG |
| **SLCO4C1** | DEG | DEG | DEG |
| **SLIT2** | DEG |  | DEG |
| **SLITRK3** | DEG | DEG | DEG |
| **SLITRK5** | DEG | DEG | DEG |
| **SMAD3** | DEG |  | DEG |
| **SMAD7** | DEG | DEG |  |
| **SMAD9** | DEG |  |  |
| **SMO** | DEG |  |  |
| **SMOX** | DEG |  | DEG |
| **SMPDL3B** | DEG | DEG | DEG |
| **SMTNL1** | DEG | DEG |  |
| **SNAI2** | DEG |  | DEG |
| **SNORA22** |  |  | DEG |
| **SNORA30** |  | DEG | DEG |
| **SNORA51** | DEG | DEG | DEG |
| **SNORD126** |  | DEG |  |
| **SNORD14E** | DEG | DEG | DEG |
| **SNORD6** | DEG |  |  |
| **SNORD69** |  |  | DEG |
| **SNORD93** | DEG | DEG | DEG |
| **snoU13** | DEG |  | DEG |
| **SOCS1** | DEG | DEG | DEG |
| **SOCS3** | DEG | DEG |  |
| **SOD2** | DEG |  | DEG |
| **SORL1** | DEG |  |  |
| **SP100** | DEG | DEG |  |
| **SP110** | DEG | DEG |  |
| **SPAG17** | DEG | DEG |  |
| **SPEG** | DEG |  | DEG |
| **SPHK1** | DEG | DEG | DEG |
| **SPNS1** |  |  | DEG |
| **SPOCD1** | DEG | DEG |  |
| **SPRY1** | DEG |  |  |
| **SPRY4** | DEG | DEG | DEG |
| **SPSB1** | DEG |  | DEG |
| **SQSTM1** | DEG |  | DEG |
| **SRGN** | DEG |  | DEG |
| **SSBP2** | DEG | DEG | DEG |
| **SSR4P1** |  |  | DEG |
| **SSTR2** | DEG | DEG |  |
| **ST3GAL4** | DEG |  | DEG |
| **ST6GAL2** |  |  | DEG |
| **ST6GALNAC2** | DEG |  |  |
| **ST7-OT4** | DEG |  |  |
| **ST8SIA4** |  |  | DEG |
| **STAB1** | DEG | DEG | DEG |
| **STARD4-AS1** | DEG | DEG | DEG |
| **STARD8** | DEG |  |  |
| **STAT1** | DEG | DEG |  |
| **STAT2** | DEG |  |  |
| **STAT4** |  | DEG | DEG |
| **STAT5A** | DEG |  | DEG |
| **STC1** | DEG | DEG | DEG |
| **STEAP1** | DEG |  |  |
| **STEAP2** | DEG |  |  |
| **STON1** | DEG |  | DEG |
| **STRA6** |  |  | DEG |
| **STX11** |  |  | DEG |
| **STXBP6** | DEG |  |  |
| **SULF1** |  |  | DEG |
| **SVIL** | DEG |  | DEG |
| **SVILP1** | DEG |  |  |
| **SYBU** | DEG | DEG | DEG |
| **SYDE2** | DEG |  | DEG |
| **SYNPO2** |  |  | DEG |
| **SYPL2** | DEG |  |  |
| **SYT1** | DEG |  |  |
| **SYT14** |  |  | DEG |
| **SYTL5** | DEG |  |  |
| **TACR2** | DEG | DEG |  |
| **TAF4B** | DEG |  |  |
| **TAP1** | DEG | DEG |  |
| **TAP2** | DEG | DEG |  |
| **TAPBP** | DEG |  |  |
| **TAPBPL** | DEG | DEG |  |
| **TAPSAR1** | DEG | DEG |  |
| **TAPT1-AS1** | DEG |  |  |
| **TBKBP1** |  | DEG |  |
| **TBX2** | DEG | DEG | DEG |
| **TBX21** | DEG | DEG |  |
| **TBX3** | DEG |  | DEG |
| **TCEAL7** | DEG | DEG | DEG |
| **TCF7** | DEG |  | DEG |
| **TDO2** | DEG |  | DEG |
| **TENM2** | DEG |  |  |
| **TET1** | DEG |  |  |
| **TEX29** | DEG | DEG |  |
| **TEX41** | DEG | DEG | DEG |
| **TFPI2** | DEG |  |  |
| **TGM2** | DEG | DEG |  |
| **THAP8** | DEG |  | DEG |
| **THRB** | DEG |  | DEG |
| **TIFA** | DEG |  |  |
| **TIMP3** | DEG | DEG | DEG |
| **TJP2** | DEG |  | DEG |
| **TLCD2** |  |  | DEG |
| **TLDC2** | DEG | DEG |  |
| **TLL1** | DEG |  | DEG |
| **TLR2** | DEG |  |  |
| **TLR3** |  | DEG |  |
| **TLR4** | DEG | DEG |  |
| **TM4SF1** | DEG |  | DEG |
| **TM7SF2** | DEG |  |  |
| **TMEM119** | DEG |  | DEG |
| **TMEM132A** | DEG |  | DEG |
| **TMEM133** | DEG |  |  |
| **TMEM144** |  |  | DEG |
| **TMEM158** | DEG | DEG | DEG |
| **TMEM169** | DEG |  | DEG |
| **TMEM170B** | DEG | DEG |  |
| **TMEM171** | DEG | DEG |  |
| **TMEM177** | DEG |  |  |
| **TMEM178B** | DEG | DEG | DEG |
| **TMEM187** | DEG | DEG | DEG |
| **TMEM200A** |  | DEG |  |
| **TMEM229B** | DEG | DEG |  |
| **TMEM239** | DEG | DEG |  |
| **TMEM246** | DEG |  |  |
| **TMEM51** | DEG |  | DEG |
| **TMEM52B** | DEG |  | DEG |
| **TMEM56** | DEG | DEG | DEG |
| **TMEM88** | DEG | DEG | DEG |
| **TMSB15A** | DEG |  |  |
| **TMTC1** | DEG |  |  |
| **TNC** | DEG | DEG | DEG |
| **TNF** | DEG |  | DEG |
| **TNFAIP2** | DEG |  | DEG |
| **TNFAIP3** | DEG | DEG | DEG |
| **TNFAIP6** | DEG |  |  |
| **TNFAIP8** | DEG |  | DEG |
| **TNFGR2** |  |  | DEG |
| **TNFRSF10D** | DEG | DEG |  |
| **TNFRSF11B** | DEG |  | DEG |
| **TNFRSF14** | DEG | DEG |  |
| **TNFRSF1B** | DEG | DEG |  |
| **TNFRSF8** | DEG | DEG |  |
| **TNFRSF9** | DEG |  | DEG |
| **TNFSF10** | DEG | DEG |  |
| **TNFSF13B** | DEG | DEG |  |
| **TNFSF14** | DEG |  |  |
| **TNFSF15** | DEG |  |  |
| **TNFSF4** | DEG |  | DEG |
| **TNIP1** | DEG |  | DEG |
| **TNIP3** | DEG |  | DEG |
| **TNNT2** | DEG | DEG | DEG |
| **TNXA** | DEG | DEG |  |
| **TOX** | DEG |  |  |
| **TOX2** | DEG |  | DEG |
| **TP53INP1** | DEG |  |  |
| **TP63** | DEG |  | DEG |
| **TPD52L1** | DEG | DEG | DEG |
| **TPST2** |  |  | DEG |
| **TRAC** |  |  | DEG |
| **TRAF1** | DEG | DEG | DEG |
| **TRAF3** |  |  | DEG |
| **TRANK1** | DEG |  |  |
| **TRBC2** |  | DEG |  |
| **TRIB1** | DEG | DEG | DEG |
| **TRIB2** | DEG | DEG |  |
| **TRIB3** | DEG | DEG | DEG |
| **TRIM16** | DEG |  | DEG |
| **TRIM16L** | DEG |  | DEG |
| **TRIM2** |  |  | DEG |
| **TRIM21** | DEG | DEG |  |
| **TRIM22** | DEG | DEG |  |
| **TRIM25** | DEG | DEG | DEG |
| **TRIM47** | DEG |  | DEG |
| **TRIM6** | DEG |  |  |
| **TRIM69** | DEG | DEG |  |
| **TRPA1** | DEG |  | DEG |
| **TSC22D3** | DEG |  | DEG |
| **TSKU** | DEG |  |  |
| **TSPAN15** |  |  | DEG |
| **TSPAN18** |  |  | DEG |
| **TTC12** |  |  | DEG |
| **TTC39A** |  |  | DEG |
| **TTC9** | DEG | DEG | DEG |
| **TTLL1** | DEG |  |  |
| **TTLL6** | DEG |  |  |
| **TTYH1** | DEG | DEG | DEG |
| **TUB** | DEG |  |  |
| **TVP23A** |  | DEG |  |
| **TYMP** | DEG | DEG |  |
| **TYRP1** | DEG |  | DEG |
| **UBA7** |  | DEG |  |
| **UBASH3B** |  |  | DEG |
| **UBD** | DEG |  |  |
| **UBE2FP1** | DEG |  |  |
| **UBE2L6** | DEG | DEG |  |
| **UCN2** | DEG | DEG | DEG |
| **UCP2** | DEG |  | DEG |
| **UCP3** |  |  | DEG |
| **UGDH-AS1** | DEG |  |  |
| **UHRF1BP1** |  | DEG | DEG |
| **ULBP1** | DEG | DEG | DEG |
| **UNC5C** | DEG |  | DEG |
| **USP18** | DEG | DEG |  |
| **USP2** | DEG |  |  |
| **USP43** | DEG |  |  |
| **VCAM1** | DEG |  | DEG |
| **VCAN-AS1** |  |  | DEG |
| **VDR** | DEG |  |  |
| **VEGFC** | DEG | DEG | DEG |
| **VEPH1** | DEG | DEG | DEG |
| **VIM** | DEG |  |  |
| **VIT** |  |  | DEG |
| **VLDLR** | DEG | DEG | DEG |
| **VNN1** | DEG |  | DEG |
| **VWA5A** | DEG |  |  |
| **WARS** | DEG | DEG |  |
| **WDR31** | DEG | DEG | DEG |
| **WNT4** | DEG |  |  |
| **WT1** | DEG | DEG | DEG |
| **WTAP** | DEG |  | DEG |
| **XAF1** | DEG | DEG |  |
| **XDH** | DEG | DEG | DEG |
| **XIRP1** | DEG | DEG |  |
| **XKR5** | DEG | DEG | DEG |
| **Y_RNA** | DEG | DEG | DEG |
| **YPEL1** | DEG | DEG | DEG |
| **YPEL3** | DEG | DEG |  |
| **ZBED3** | DEG |  | DEG |
| **ZBED3-AS1** | DEG |  | DEG |
| **ZBTB8B** | DEG |  |  |
| **ZC3H12A** | DEG |  | DEG |
| **ZC3H12B** | DEG |  |  |
| **ZC3H6** | DEG | DEG | DEG |
| **ZCCHC5** | DEG |  | DEG |
| **ZDHHC4P1** |  | DEG |  |
| **ZFP36** |  |  | DEG |
| **ZMAT1** |  | DEG |  |
| **ZMAT3** | DEG | DEG |  |
| **ZMIZ1** |  |  | DEG |
| **ZMIZ1-AS1** | DEG |  |  |
| **ZMYM3** |  |  | DEG |
| **ZMYND12** | DEG |  |  |
| **ZNF185** | DEG | DEG | DEG |
| **ZNF19** |  | DEG |  |
| **ZNF25** | DEG |  |  |
| **ZNF267** | DEG |  | DEG |
| **ZNF385B** |  |  | DEG |
| **ZNF385D** | DEG |  | DEG |
| **ZNF395** | DEG |  | DEG |
| **ZNF467** |  | DEG |  |
| **ZNF469** | DEG | DEG | DEG |
| **ZNF516** | DEG |  |  |
| **ZNF572** | DEG |  |  |
| **ZNF627** | DEG |  | DEG |
| **ZNF697** | DEG |  | DEG |
| **ZNFX1** | DEG | DEG |  |
| **ZRANB2-AS1** | DEG | DEG | DEG |
| **ZSWIM4** | DEG |  | DEG |
