# Supplementary material for: Co-Exposure of Cardiomyocytes to IFN-γ and TNF-α Induces Mitochondrial Dysfunction and Nitro-Oxidative Stress: Implications for the Pathogenesis of Chronic Chagas Disease Cardiomyopathy
Source: Front Immunol. 2021 Nov 11;12:755862. doi: 10.3389/fimmu.2021.755862 (PMC8632642; doi:10.3389/fimmu.2021.755862)
Supplement: Supplementary file 10 [file Table_5.docx]

**Supplementary Table 5:** Peaked differentially expressed mitochondrial genes in cytokines-stimulated AC16 cells

|  | **NS vs IFN-γ+TNF-α-stimulated AC16 (12 hours)** | |
| --- | --- | --- |
| **Gene** | **Fold change** | **Adjusted pvalue** |
| OAS2 | 23.13 | 1.9E-43 |
| TAP1 | 18.32 | 2.0E-120 |
| IFIT3 | 15.31 | 9.3E-91 |
| SOD2 | 13.85 | 6.3E-34 |
| CMPK2 | 11.42 | 1.0E-15 |
| LAP3 | 9.81 | 7.0E-153 |
| ACSL5 | 9.04 | 1.9E-20 |
| OAS1 | 8.91 | 2.6E-17 |
| TGM2 | 7.38 | 2.1E-16 |
| XAF1 | 6.39 | 4.4E-40 |
| NPTX1 | 5.77 | 6.0E-09 |
| G0S2 | 5.25 | 2.0E-12 |
| BID | 4.69 | 6.0E-33 |
| NFKB1 | 4.35 | 1.1E-59 |
| PPIF | 4.10 | 1.8E-20 |
| MRPS24 | 3.77 | 3.5E-08 |
| RSAD2 | 3.74 | 2.5E-05 |
| LACTB | 3.60 | 2.0E-29 |
| IFI27 | 3.43 | 6.0E-06 |
| SLC25A37 | 3.39 | 2.0E-18 |
| ACO1 | 3.32 | 2.0E-66 |
| KYNU | 3.31 | 2.8E-05 |
| PSMA6 | 3.23 | 1.0E-10 |
| PMAIP1 | 3.00 | 5.0E-27 |
| SLC25A22 | 2.87 | 1.0E-12 |
| MOAP1 | -2.92 | 1.3E-11 |
| HOXB9 | -2.92 | 3.0E-12 |
| UCP2 | -2.93 | 2.0E-06 |
| ALDH4A1 | -3.01 | 4.2E-10 |
| GPT2 | -3.04 | 2.0E-08 |
| ALDH5A1 | -3.08 | 1.0E-11 |
| NT5M | -3.09 | 3.0E-07 |
| MAPK10 | -3.13 | 8.0E-06 |
| C10orf10 | -3.24 | 1.0E-05 |
| FAM213A | -3.32 | 3.0E-05 |
| REEP1 | -3.43 | 6.0E-12 |
| KIAA1683 | -3.43 | 6.1E-08 |
| CRYAB | -3.44 | 1.6E-04 |
| EFHD1 | -3.48 | 2.8E-07 |
| MGARP | -3.65 | 1.1E-05 |
| MPP7 | -3.68 | 5.0E-18 |
| PCK2 | -3.84 | 8.7E-07 |
| ALDH1L2 | -4.19 | 1.0E-08 |
| ACADL | -4.69 | 7.0E-09 |
| DMGDH | -4.94 | 1.0E-10 |
| PPM1E | -5.16 | 1.1E-13 |
