# Supplementary material for: Co-Exposure of Cardiomyocytes to IFN-γ and TNF-α Induces Mitochondrial Dysfunction and Nitro-Oxidative Stress: Implications for the Pathogenesis of Chronic Chagas Disease Cardiomyopathy
Source: Front Immunol. 2021 Nov 11;12:755862. doi: 10.3389/fimmu.2021.755862 (PMC8632642; doi:10.3389/fimmu.2021.755862)
Supplement: Supplementary file 11 [file Table_6.docx]

**Supplementary Table 6:** Mitochondrial genes toxicity pathways

| **Mitochondrial genes toxicity pathways** | **-log(p-value)** | **Ratio** | **Molecules** |
| --- | --- | --- | --- |
| Decreases Transmembrane Potential of Mitochondria and Mitochondrial Membrane | 6.97 | 0.0522 | BID,IFI6,NFKB1,PMAIP1,SOD2,TGM2,UCP2 |
| Fatty Acid Metabolism | 5.99 | 0.0513 | ACADL,ACSL5,ALDH4A1,ALDH5A1,CPT1A,EHHADH |
| Aryl Hydrocarbon Receptor Signaling | 4.02 | 0.0311 | ALDH1L2,ALDH4A1,ALDH5A1,NFKB1,TGM2 |
| Mitochondrial Dysfunction | 3.9 | 0.0292 | ACO1,CPT1A,MAPK10,SOD2,UCP2 |
| Renal Necrosis/Cell Death | 3.42 | 0.0128 | BID,CASP4,CRYAB,HK2,MOAP1,NFKB1,PMAIP1,SOD2 |
| Recovery from Ischemic Acute Renal Failure (Rat) | 3.15 | 0.143 | CRYAB,SGK1 |
| LPS/IL-1 Mediated Inhibition of RXR Function | 3.11 | 0.0197 | ACSL5,ALDH1L2,ALDH4A1,ALDH5A1,CPT1A |
| Cardiac Necrosis/Cell Death | 2.69 | 0.0158 | CRYAB,HK2,MMP2,PPIF,SOD2 |
| Liver Necrosis/Cell Death | 2.6 | 0.0151 | BID,HK2,MOAP1,NFKB1,SOD2 |
| Oxidative Stress | 1.94 | 0.0351 | NFKB1,SOD2 |
| PXR/RXR Activation | 1.83 | 0.0308 | CPT1A,PCK2 |
| Xenobiotic Metabolism Signaling | 1.76 | 0.0115 | ALDH1L2,ALDH4A1,ALDH5A1,NFKB1 |
| Vasopressin-induced Genes in Inner Medullary Renal Collecting Duct Cells (Rat) | 1.65 | 0.125 | SGK1 |
| Renal Ischemic Resistance Panel (Rat) | 1.55 | 0.1 | DNM3 |
| Mechanism of Gene Regulation by Peroxisome Proliferators via PPARα | 1.52 | 0.0211 | EHHADH,NFKB1 |
| Cardiac Hypertrophy | 1.5 | 0.0096 | CPT1A,CRYAB,MMP2,NFKB1 |
| LXR/RXR Activation | 1.32 | 0.0163 | ARG2,NFKB1 |
| Increases Depolarization of Mitochondria and Mitochondrial Membrane | 1.3 | 0.0556 | TGM2 |
| Long-term Renal Injury Anti-oxidative Response Panel (Rat) | 1.3 | 0.0556 | SOD2 |
| FXR/RXR Activation | 1.3 | 0.0159 | MAPK10,PCK2 |
| Cardiac Fibrosis | 1.29 | 0.0102 | ACADL,NFKB1,SOD2 |
| Increases Liver Steatosis | 1.27 | 0.0152 | BID,G0S2 |
| Increases Heart Failure | 1.24 | 0.0476 | PPIF |
| Genes associated with Chronic Allograft Nephropathy (Human) | 1.24 | 0.0476 | MMP2 |
| Increases Liver Hyperplasia/Hyperproliferation | 1.23 | 0.0145 | NFKB1,SOD2 |
| Nongenotoxic Hepatocarcinogenicity Biomarker Panel | 1.22 | 0.0455 | TAP1 |
| Positive Acute Phase Response Proteins | 1.09 | 0.0333 | SOD2 |
| Hepatic Stellate Cell Activation | 1.02 | 0.0286 | NFKB1 |
| Hepatic Cholestasis | 0.997 | 0.0106 | MAPK10,NFKB1 |
| PPARα/RXRα Activation | 0.982 | 0.0104 | ACADL,NFKB1 |
| RAR Activation | 0.971 | 0.0102 | MAPK10,NFKB1 |
| Pro-Apoptosis | 0.948 | 0.0238 | BID |
| Increases Transmembrane Potential of Mitochondria and Mitochondrial Membrane | 0.877 | 0.02 | SOD2 |
| Liver Proliferation | 0.76 | 0.0076 | BID,NFKB1 |
| TR/RXR Activation | 0.672 | 0.0119 | UCP2 |
| Increases Liver Hepatitis | 0.637 | 0.0109 | BID |
| p53 Signaling | 0.609 | 0.0101 | PMAIP1 |
| Increases Renal Damage | 0.598 | 0.0098 | BID |
| Hepatic Fibrosis | 0.58 | 0.0057 | MMP2,NFKB1 |
| NRF2-mediated Oxidative Stress Response | 0.305 | 0.0042 | SOD2 |
| NF-κB Signaling | 0 | 0.0017 | NFKB1 |
